# Effects of negative ions on equilibrium solar plasmas in the fabric of gravito-electrostatic sheath model

**DOI:** 10.1038/s41598-024-66774-8

**Published:** 2024-07-12

**Authors:** Pankaj Sarma, Pralay Kumar Karmakar

**Affiliations:** Department of Physics, Tezpur University, Napaam, Tezpur, Assam 784028 India

**Keywords:** Astronomy and astrophysics, Astronomy and planetary science, Physics

## Abstract

The gravito-electrostatic sheath (GES) model, exploring the solar wind plasma (SWP) origin from the solar interior plasma (SIP) via the solar surface boundary (SSB), is revaluated by including realistic negative ionic species. A constructive numerical analysis of the structuring equations shows that the SIP volume shrinks with an increase in the negative ion concentration. This shrinking nature is independent of ion mass and plasma temperature. The electric potential is insensitive to the negative ion concentration, mass, and plasma temperature. The solar plasma flow dynamics is studied with the Mach number and current density profiles. The sonic transition of the SWP depends on the *T*_*i*_*/T*_*e*_-ratio. The current density responds to the negative ion density and *T*_*i*_*/T*_*e*_^−^ratio in both the SIP and SWP. A deviation from the local quasi-neutrality state is observed in the SIP. The GES model equations result in a modified GES-Bohm sheath criterion in a well justifiable and validated form. The obtained results are then compared with the various observed outcomes and previous GES-based predictions. The relevance of this multi-parametric solar plasma analysis is lastly emphasized on the basis of the current solar research progressions.

## Introduction

The entire solar plasma system consisting of the bounded Sun and its unbounded surrounding atmosphere has been a mysterious plasma laboratory for decades yet to be well understood. The most challenging aspect of such solar systems lies basically in understanding the complex nature of the solar wind flow dynamics and associated structure formation^1,2^. It is extensively found in the literature that the investigation of the normal stellar systems, like the Sun and its atmosphere, has been performed on various plausible theoretical model formalisms. It primarily includes the Parker solar wind dynamical model based on the neutral gas-based hydrodynamics^3^, Chamberlain’s exospheric model based on kinetic treatment of the solar wind particles^4^ and the gravito-electrostatic sheath (GES) model based on the application of the laboratory plasma-wall interaction mechanisms to the astrophysical spatiotemporal scales^5–11^. It is noteworthy here that the latter model has been successful enough in explaining the surface origin of the solar wind plasma (SWP) from the solar interior plasma (SIP) through the diffused solar surface boundary (SSB), from the former models^5^. Recently, a realistically modified version of the original GES model to see the solar plasma flow dynamics has been reported. It has been able to depict a fair resemblance of sensible solar properties to the recent solar observational scenarios^11^, and so forth. This is how a fair reliability and validation of the plasma-based GES model formalism in realistic thermo-statistical environments has been well bolstered.

It may be noteworthy that, one of the unaddressed major aspects in this important direction of solar astrophysics lies in the fact that the presence of diverse negative ions in the solar dynamics has always been ignored in the solar plasma theorization in the past as far as seen. The role of negative ions is well known to be unavoidable in the electromagnetic structurization of such stellar systems. It is found, in the literature^12^, that this has been one of the significant problems in stellar astrophysics in the early decades of the twentieth century to explain radiation absorption in the exterior regions of cool stellar environs, such as the Sun and its atmosphere. It has been believed that the sustained opacity in the visible frequencies of the electromagnetic spectrum is caused due to the presence of the metal atoms with an abundance ratio 1:50 with respect to hydrogen. However, in 1939, R. Wildt has reported that the H^−^ ion dominates the visible opacity in the sun-like stars with photospheric temperature less than 7000 K, with the metal:hydrogen abundance ratio 1:1000^12,13^. It is now evident that, in the cool stellar plasma systems, the photoionization of the metal atoms provides the electrons that produce the H^-^ ion^12^. With advancement in the spectrophotometric analytical techniques, it is now well established that except H^−^, several other negative atomic as well as molecular negative ions, like Cl^−^, C^−^, S^−^, OH^−^, C_2_^−^, CN^−^, SH^−^ and H_2_O^−^ have significant contributions towards the continuous opacity of the late type stellar spectra^14,15^. Nevertheless, the role of realistic negative ions, as highlighted previously, in the stellar plasma evolution, has never been explored. In fact, any investigation of the solar plasma dynamics and equilibrium features in the realistic circumstances without considering the role of negative ionic species is both improper and inadequate. Accordingly, such studies pose another venture in exploring the solar plasma model characteristics moderated in the presence of diverse negative ionic species kinetically evolving in the entire solar plasma system in a realistic sense.

As a consequence of the above motivating factual scenarios, the presented investigation characterizes the role of the negative ions in the solar plasma evolution on the original GES-fabric founded on the basic plasma-wall interaction physics^5^. It illustratively portrays the relevancy of negative ions in the equilibrium solar plasma processes and subsequent equilibrium structurization in the bi-scaled solar plasma system (SIP and SWP) coupled via the interfacial SSB. In addition, the role of the negative ions in the sheath formation processes on the astrophysical spatiotemporal scales is elaborately explored followed by a comparative reliability assessment in light of the key observed solar properties drawn from various astronomical and observational missions, such as the Parker Solar Probe, Helios, Wind, Cluster, and so forth^14–18^. A validated contrast is herewith illustratively established with the help of the various previous GES formalisms. It includes GES-1^5^ and GES-2^11^. Their relative deviations against GES-1 are accordingly designated as “GES:1–2” and “GES:1–3”. It clearly represents the relative contrast of GES-2 (recent analysis) and GES-3 (present analysis) against GES-1 respectively as in Appendix F. On realistic applications, the proposed semi-analytic study could be extensively useful in understanding the role of the naturalistic negative ions in the equilibrium structure analysis of the Sun and other sun-like stars with intermediate surface temperatures in a broader stellar astrophysical perspective. It clearly suggests an extensive scope to study stellar structure evolution and stability based on such GES-centric plasma model formalisms.

It is noteworthy to mention here that the astrophysical plasma sheath structures have been well reported elsewhere in the solar system, like on the sunlit regions of the Moon. Such plasma sheath environments are created by the interaction of highly energetic photons as well as the solar wind particles with the lunar surface boundary^19^. It is pertinent to note here that such lunar sheath structural profiles can be well modelled with the non-Maxwellian solar wind electron behaviours^20–22^. Here, in the present context as well, this investigation reveals that the supersonic solar wind particles are of such non-equilibrium thermo-statistical nature (non-Maxwellian). As a consequence, the presented GES-based solar wind model predictions on the equilibrium properties of the supersonic and hypersonic constituent solar wind particles stands well in consistency with the realistic lunar sheath scenarios as very recently reported in the literature^19^.

## Model formulation

We consider the entire solar plasma system as a complex fluid medium composed of three constitutive species, such as electrons, positive ions (protons), and negative ions (heterogeneous). The non-gravitating lighter (inertialess) electrons are described by the Maxwell–Boltzmann thermo-statistical distribution law. The gravitating heavier (inertial) positive (negative) ions are treated in the fluidic framework as per the first principles. The entire solar plasma model is assumed to be in a spherically symmetric geometry. It enables us to simplify the 3-D solar problem as a reduced 1-D one. It deals with the radial dependency of the relevant physical parameters only, because the polar and azimuthal counterparts are relaxed fully without any loss of generality^23,24^. The plasma species form an isothermal hydrostatic homogeneous equilibrium configuration throughout the entire bounded solar plasma system with the presumed global quasi-neutrality. This quasi-neutrality is well justifiable here on the grounds of asymptotically zero-value of the Debye-to-Jeans length scale ratio as already found in diverse realistic astronomical circumstances^5^.

It is clearly perceptible from the gravito-thermal coupling constants of the plasma species^5,6^ that the inertialess electrons are capable of nimbly flying away from the considered plasma volume against the self-gravitational potential barrier at the cost of their thermal (kinetic) energy alone unlike the inertial ions. In a broader sense, the constitutive positive and negative ions cannot overcome the self-gravitational barrier hindrance with their thermal energy alone. This is the key phenomenon responsible for the consequent space-charge polarization effects, leading thereby to the formation of the GES structure. It is noteworthy here that, the basic physical insights of the original GES model are well founded on the same plasma-wall interaction processes, as reported previously in analogy with the laboratory confined plasmas as well^5,6^. Thus, with all these factual reservations in our model formulation, we propose a continued exploration on the equilibrium solar plasma characteristic features in the presence of diverse negative ionic species in real astronomical circumstances for the first time.

**Figure 1:**
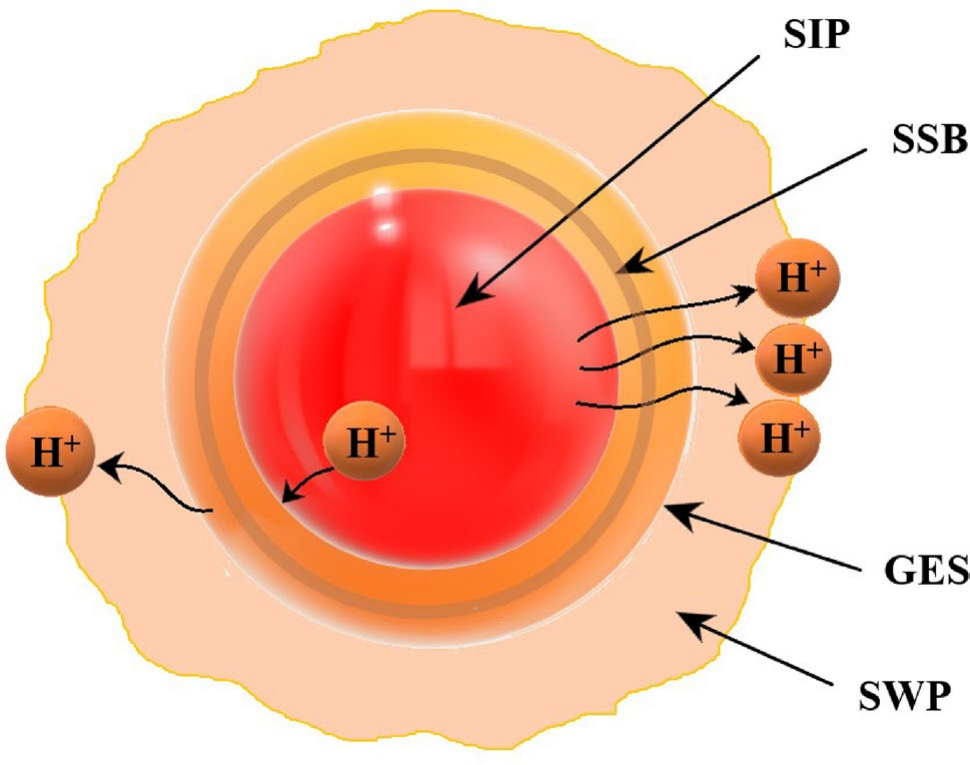
A cartoonist sketch of the solar plasma system in the GES model fabric.

As in Fig. 1, we depict the solar plasma system according to the GES model formalism for the sake of a clear visualization of the readers. Accordingly, it is divided concentrically as the SIP and SWP as already mentioned above. The solar wind particles travel through the GES with a subsonic speed via the SSB. The constitutive particles achieve supersonic or hypersonic speed beyond the SSB on the unbounded scale^5^. However, the loss of positive ions from the GES is compensated by other positive ions immediately that move with supersonic speed for well endurance of the complete GES structure. The detailed analysis of the same is illustratively performed in Appendix D.

### SIP formalism

The considered SIP system consists of inertialess electrons (Boltzmann), inertial positive ions (fluid), and inertial negative ions (fluid) coupled via the closing electro-gravitational Poisson equations representing potential evolutions at the cost of density fields. The electronic dynamics is accordingly governed by the Maxwell–Boltzmann thermo-statistical distribution law expressed in all the generic notations^5^ as

1 $$n_{e} = n_{e0} {\text{exp}}\,\left( {\frac{e\phi }{{k_{B} T_{e} }}} \right).$$

The dynamics of the constitutive positive ions is governed by the continuity equation (flux conservation), momentum equation (force balance), and isothermal equation of state (barotropic description) given respectively in the generic notations^11^ as

2 $$\partial_{t} n_{ + } + v_{ + } \left( {\partial_{r} n_{ + } } \right) + n_{ + } \left( {\partial_{r} v_{ + } } \right) + \frac{2}{r}\left( {n_{ + } v_{ + } } \right) = 0,$$

3 $$m_{ + } n_{ + } \left[ {\partial_{t} v_{ + } + v_{ + } \left( {\partial_{r} v_{ + } } \right)} \right] = - en_{ + } \left( {\partial_{r} \phi } \right) - \partial_{r} P_{T} - m_{ + } n_{ + } \left( {\partial_{r} \psi } \right),$$

4 $$P_{T + } = n_{ + } k_{B} T_{ + } .$$

The negative ion dynamics is governed by the similar equations cast respectively as

5 $$\partial_{t} n_{ - } + v_{ - } \left( {\partial_{r} n_{ - } } \right) + n_{ - } \left( {\partial_{r} v_{ - } } \right) + \frac{2}{r}\left( {n_{ - } v_{ - } } \right) = 0,$$

6 $$m_{ - } n_{ - } \left[ {\partial_{t} v_{ - } + v_{ - } \left( {\partial_{r} v_{ - } } \right)} \right] = en_{ - } \left( {\partial_{r} \phi } \right) - \partial_{r} P_{T} - m_{ - } n_{ - } \left( {\partial_{r} \psi } \right),$$

7 $$P_{T - } = n_{ - } k_{B} T_{ - } .$$

The model closure is finally obtained with the help of the electro-gravitational Poisson equations for the corresponding potential distributions given respectively in usual notations as

8 $$\partial_{r}^{2} \phi + \frac{2}{r}\left( {\partial_{r} \phi } \right) = \frac{e}{{\varepsilon_{0} }}\left( {n_{e} + n_{ - } - n_{ + } } \right),$$

9 $$\partial_{r}^{2} \psi + \frac{2}{r}\left( {\partial_{r} \psi } \right) = 4\pi \,G\,\left( {m_{ + } n_{ + } + m_{ - } n_{ - } } \right).$$

Finally, the electric current density associated with the SIP is cast in usual symbols as

10 $$j_{SIP} = n_{ + } e\left[ { - \sqrt {2r\left( {\partial_{r} \psi } \right)} - \sqrt {\left( {\frac{2e}{{m_{ + } }}} \right)r\left( {\partial_{r} \phi } \right)} } \right] + n_{ - } e\left[ { - \sqrt {2r\left( {\partial_{r} \psi } \right)} + \sqrt {\left( {\frac{2e}{{m_{ - } }}} \right)r\left( {\partial_{r} \phi } \right)} } \right] + n_{e} e\sqrt {\left( {\frac{2e}{{m_{e} }}} \right)r\left( {\partial_{r} \phi } \right)} .$$

All the customary dimensional notations with their physical significances in the above unnormalized equations are detailed in Appendix A. Now, in order to explore the equilibrium solar structure numerically according to our model formalism, Eqs. (1–10) are first expressed in time-stationary normalized form after an adopted standard astronomical normalization scheme^5^, as in Appendix B. Accordingly Eqs. (1–10) can respectively be written in the astrophysically normalized form cast as

11 $$N_{e} = {\text{exp}}\,\left( \Phi \right)$$

12 $$\frac{1}{{N_{ + } }}\left( {\partial_{\xi } N_{ + } } \right) + \frac{1}{{M_{ + } }}\left( {\partial_{\xi } M_{ + } } \right) + \frac{2}{\xi } = 0,$$

13 $$M_{ + } \left( {\partial_{\xi } M_{ + } } \right) = - \partial_{\xi } \Phi - \left( {\frac{{T_{ + } }}{{T_{e} }}} \right)\frac{1}{{N_{ + } }}\left( {\partial_{\xi } N{}_{ + }} \right) - \partial_{\xi } \Psi ,$$

14 $$P_{T + }^{*} = \left( {\frac{{n_{ + 0} k_{B} T_{ + } }}{{P_{0} }}} \right)N_{ + } ,$$

15 $$\frac{1}{{N_{ - } }}\left( {\partial_{\xi } N_{ - } } \right) + \frac{1}{{M_{ - } }}\left( {\partial_{\xi } M_{ - } } \right) + \frac{2}{\xi } = 0,$$

16 $$M_{ - } \left( {\partial_{\xi } M_{ - } } \right) = \frac{{m_{ + } }}{{m_{ - } }}\left( {\partial_{\xi } \Phi } \right) - \frac{{m_{ + } }}{{m_{ - } }}\left( {\frac{{T_{ - } }}{{T_{e} }}} \right)\frac{1}{{N_{ - } }}\left( {\partial_{\xi } N{}_{ - }} \right) - \partial_{\xi } \Psi ,$$

17 $$P_{T - }^{*} = \left( {\frac{{n_{ - 0} k_{B} T_{ - } }}{{P_{0} }}} \right)N_{ - } ,$$

18 $$\partial_{\xi }^{2} \Phi + \frac{2}{\xi }\left( {\partial_{\xi } \Phi } \right) = \left( {\frac{{\lambda_{J} }}{{\lambda_{De} }}} \right)^{2} \left[ {\left( {1 - \delta } \right)N_{e} + \delta \,N_{ - } - N_{ + } } \right],$$

19 $$\partial_{\xi }^{2} \Psi + \frac{2}{\xi }\left( {\partial_{\xi } \Psi } \right) = \left[ {N_{ + } + \delta \,N_{ - } } \right],$$

20 $$J_{SIP} = N_{ + } \left[ { - \sqrt {2\xi \,{(}\partial_{\xi } \Psi {)}} - \sqrt {2\xi \,{(}\partial_{\xi } \Phi {)}} } \right] + \delta \,N_{ - } \left[ { - \sqrt {2\xi \left( {\partial_{\xi } \Psi } \right)} + \sqrt {2\xi \left( {\frac{{m_{ + } }}{{m_{ - } }}} \right)\left( {\partial_{\xi } \Phi } \right)} } \right] + \left( {1 - \delta } \right)N_{e} \sqrt {2\xi \left( {\frac{{m_{ + } }}{{m_{e} }}} \right)\left( {\partial_{\xi } \Phi } \right)} .$$

It is worth mentioning that time-stationary coupled Eqs. (11–20) govern the steady-state dynamics of the self-gravitating SIP and the subsequent equilibrium structure of the GES in a closed form modified due to the presence of considered negative ions. The normalized equations above are now coupled to obtain a closed set of time-stationary first-order differential equations (ODEs) for the description of the equilibrium SIP evolution. It is now seen from the above that the resulting SIP system is sensitive to the relevant parametric variations, such as the equilibrium negative-to-positive ion density ratio (*δ*), positive-to-negative ion mass ratio (*m*_*i*_*/m*_−_), positive ion-to-electron temperature ratio (*T*_*i*_*/T*_*e*_), and negative ion-to-electron temperature ratio (*T*_−_*/T*_*e*_). It is to be noted, specifically in the mathematical perspective that the *δ-*sensitivity arises from Eqs. (18–20). The *m*_*i*_*/m*_−_sensitivity originates from Eqs. (16) and (20). The *T*_*i*_*/T*_*e*_- and *T*_−_*/T*_*e*_-sensitivities appear from Eqs. (13) and (16), respectively. The fourth-order Runge–Kutta (RK-IV) method is systematically applied for the steady-state SIP analysis with the sensible initial and input values, as highlighted in Appendix C, using MATLAB numerically^5,25^. In the subsequent analysis, we replace the positive ionic symbol “ + ”, as in the equations, with “i”, as per usual convention.

### SWP formalism

It is already known that, as the bounded SIP transforms into the unbounded SWP, the Newtonian gravity changes from the self-gravity (extended source) to an external gravity (point source of mass *M*_*Θ*_) without any loss in the macroscopic non-local description of the integrated original solar plasma system^5^. Accordingly, the SWP-fluid dynamics is dictated by a similar set of governing equations as in the SIP case, except the plasma self-gravity (internal) now replaced with the inverse-square point-like Newtonian gravity (external). It hereby makes the self-gravitational Poisson equation now redundant justifiably^5^.

The SWP constitutive electrons follow the same Maxwell–Boltzmann thermo-statistical distribution law as in the SIP description expressed as

21 $$n_{e} = n_{e0} {\text{exp}}\,\left( {\frac{e\phi }{{k_{B} T_{e} }}} \right).$$

The continuity equation, momentum equation and the equation of state followed by the positive ions are given respectively in a similar manner as

22 $$\partial_{t} n_{ + } + v_{ + } \left( {\partial_{r} n_{ + } } \right) + n_{ + } \left( {\partial_{r} v_{ + } } \right) + \frac{2}{r}\left( {n_{ + } v_{ + } } \right) = 0,$$

23 $$m_{ + } n_{ + } \left[ {\partial_{t} v_{ + } + v_{ + } \left( {\partial_{r} v_{ + } } \right)} \right] = - en_{ + } \left( {\partial_{r} \phi } \right) - \partial_{r} P_{T} - m_{ + } n_{ + } \left( {\frac{{GM_{\Theta } }}{{r^{2} }}} \right),$$

24 $$P_{T + } = n_{ + } k_{B} T_{ + } .$$

Similarly, the equations dictating the dynamics of the negative ions in the SWP are cast respectively as

25 $$\partial_{t} n_{ - } + v_{ - } \left( {\partial_{r} n_{ - } } \right) + n_{ - } \left( {\partial_{r} v_{ - } } \right) + \frac{2}{r}\left( {n_{ - } v_{ - } } \right) = 0,$$

26 $$m_{ - } n_{ - } \left[ {\partial_{t} v_{ - } + v_{ - } \left( {\partial_{r} v_{ - } } \right)} \right] = en_{ - } \left( {\partial_{r} \phi } \right) - \partial_{r} P_{T} - m_{ - } n_{ - } \left( {\frac{{GM_{\Theta } }}{{r^{2} }}} \right),$$

27 $$P_{T - } = n_{ - } k_{B} T_{ - } .$$

The diverse constitutive species are coupled together with the help of the electrostatic Poisson equation and the net electric current density evolution equation in the SWP medium are respectively written in the customary symbols as

28 $$\partial_{r}^{2} \phi + \frac{2}{r}\left( {\partial_{r} \phi } \right) = \frac{e}{{\varepsilon_{0} }}\left( {n_{e} + n_{ - } - n_{ + } } \right),$$

29 $$j_{SWP} = n_{ + } e\left[ { - \sqrt {2r\left( {\frac{{GM_{\Theta } }}{{r^{2} }}} \right)} - \sqrt {\left( {\frac{2e}{{m_{ + } }}} \right)r\left( {\partial_{r} \phi } \right)} } \right] + n_{ - } e\left[ { - \sqrt {2r\left( {\frac{{GM_{\Theta } }}{{r^{2} }}} \right)} + \sqrt {\left( {\frac{2e}{{m_{ - } }}} \right)r\left( {\partial_{r} \phi } \right)} } \right] + n_{e} e\sqrt {\left( {\frac{2e}{{m_{e} }}} \right)r\left( {\partial_{r} \phi } \right)} .$$

In order for a scale-invariant steady-state SWP description, Eqs. (21–29) are transformed into the corresponding time-stationary normalized form, following the same astronomical normalization scheme as employed in the SIP portrayal, presented respectively as

30 $$N_{e} = {\text{exp}}\,\left( \Phi \right),$$

31 $$\frac{1}{{N_{ + } }}\left( {\partial_{\xi } N_{ + } } \right) + \frac{1}{{M_{ + } }}\left( {\partial_{\xi } M_{ + } } \right) + \frac{2}{\xi } = 0,$$

32 $$M_{ + } \left( {\partial_{\xi } M_{ + } } \right) = - \partial_{\xi } \Phi - \left( {\frac{{T_{ + } }}{{T_{e} }}} \right)\frac{1}{{N_{ + } }}\left( {\partial_{\xi } N{}_{ + }} \right) - \left( {\frac{1}{{c_{s}^{2} \lambda_{J} }}} \right)\frac{{GM_{\Theta } }}{{\xi^{2} }},$$

33 $$P_{T + }^{*} = \left( {\frac{{n_{ + 0} k_{B} T_{ + } }}{{P_{0} }}} \right)N_{ + } ,$$

34 $$\frac{1}{{N_{ - } }}\left( {\partial_{\xi } N_{ - } } \right) + \frac{1}{{M_{ - } }}\left( {\partial_{\xi } M_{ - } } \right) + \frac{2}{\xi } = 0,$$

35 $$M_{ - } \left( {\partial_{\xi } M_{ - } } \right) = \frac{{m_{ + } }}{{m_{ - } }}\left( {\partial_{\xi } \Phi } \right) - \frac{{m_{ + } }}{{m_{ - } }}\left( {\frac{{T_{ - } }}{{T_{e} }}} \right)\frac{1}{{N_{ - } }}\left( {\partial_{\xi } N{}_{ - }} \right) - \left( {\frac{1}{{c_{s}^{2} \lambda_{J} }}} \right)\frac{{GM_{\Theta } }}{{\xi^{2} }},$$

36 $$P_{T - }^{*} = \left( {\frac{{n_{ - 0} k_{B} T_{ - } }}{{P_{0} }}} \right)N_{ - } ,$$

37 $$\partial_{\xi }^{2} \Phi + \frac{2}{\xi }\left( {\partial_{\xi } \Phi } \right) = \left( {\frac{{\lambda_{J} }}{{\lambda_{De} }}} \right)^{2} \left[ {\left( {1 - \delta } \right)N_{e} + \delta \,N_{ - } - N_{ + } } \right],$$

38 $$J_{SWP} = N_{ + } \left[ { - \sqrt {\frac{2}{{c_{s}^{2} \lambda_{J} }}\left( {\frac{{GM_{\Theta } }}{\xi }} \right)} - \sqrt {2\xi \,{(}\partial_{\xi } \Phi {)}} } \right] + \delta \,N_{ - } \left[ { - \sqrt {\frac{2}{{c_{s}^{2} \lambda_{J} }}\left( {\frac{{GM_{\Theta } }}{\xi }} \right)} + \sqrt {2\xi \left( {\frac{{m_{ + } }}{{m_{ - } }}} \right)\left( {\partial_{\xi } \Phi } \right)} } \right] + \left( {1 - \delta } \right)N_{e} \sqrt {2\xi \left( {\frac{{m_{ + } }}{{m_{e} }}} \right)\left( {\partial_{\xi } \Phi } \right)} .$$

It is noteworthy that time-stationary Eqs. (30–38) dictate the steady-state dynamics of the non-gravitating SWP and its subsequent equilibrium flow dynamics relative to the SSB as its base in a closed analytic form, modified in the presence of considered negative ionic species. The resulting SWP system, as clearly evident from the coupled governing equations (Eqs. 30–38), is sensitive to the relevant parametric variations, such as *δ*, *m*_*i*_*/m*_-_, *T*_*i*_*/T*_*e*_, and *T*_−_*/T*_*e*_ (as in the SIP). It is to be noted that the *δ-*sensitivity of the SWP system arises from Eqs. 37 and 38. The *m*_*i*_*/m*_−_sensitivity originates from Eqs. (35) and (38). The *T*_*i*_*/T*_*e*_^−^ and *T*_−_*/T*_*e*_-sensitivities in the SWP appear from Eqs. (32) and (35), respectively. Accordingly, applying the SIP-specified initial and input values^5^, as given in Appendix C, the same RK-IV method, as in the SIP, is used herein for the SWP description numerically in a similar MATLAB computational platform^25^.

It is be marked here that the input initial values of the relevant solar plasma parameters for the numerical analysis to proceed are obtained with the help of the basic principles of nonlinear stability analysis (fixed-point treatment) judiciously^5^. The numerically obtained values of the relevant solar plasma parameters at the SSB are taken as the input initial values for the SWP and so forth. It hereby offers a continuous and smooth transition of the bounded subsonic SIP to the unbounded supersonic SWP without any violation of fundamental physical principles.

## Results and discussions

**Figure 2:**
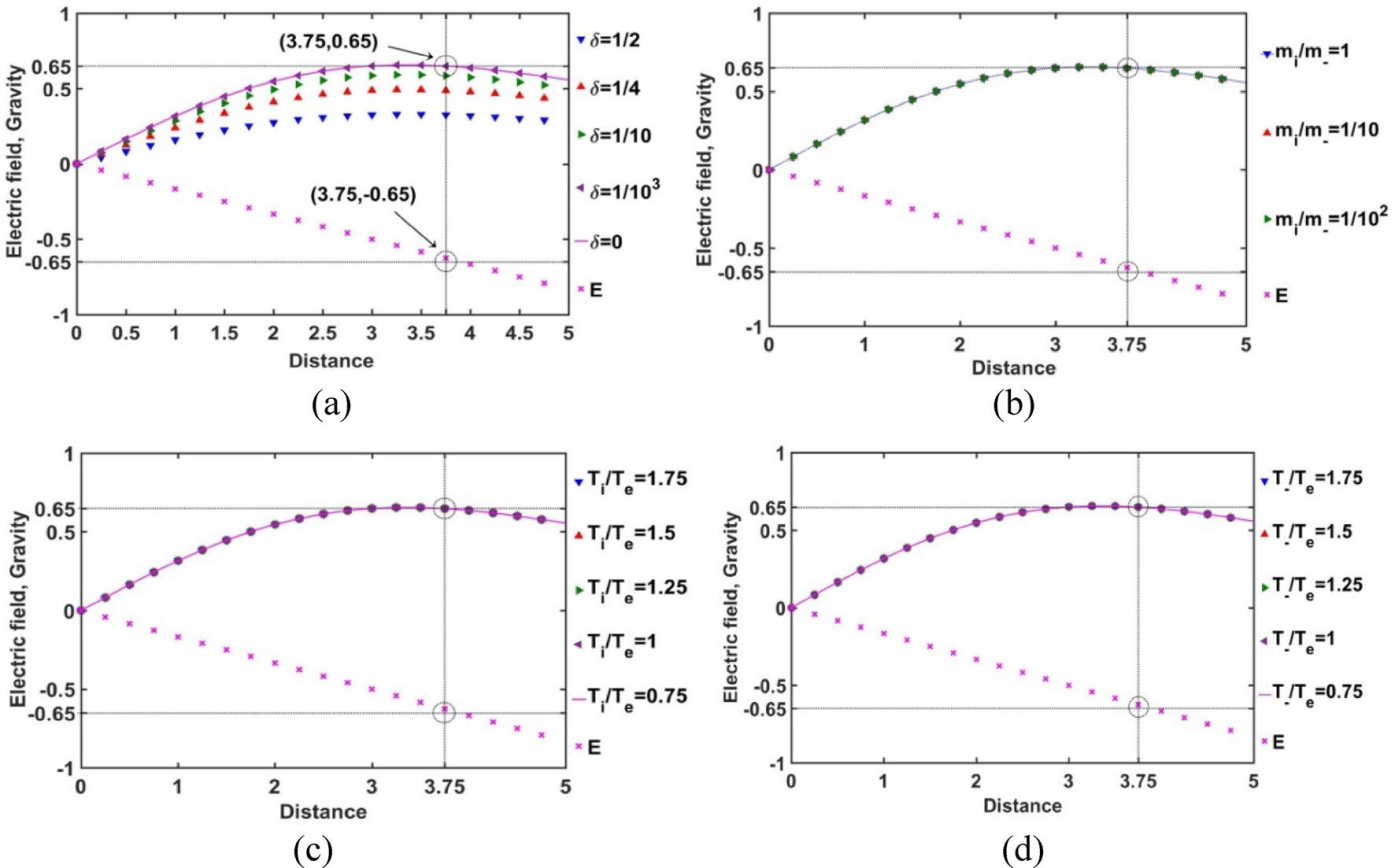
Variation of the normalized electric field (*E*) and self-gravitational field (gravity) strength with the Jeans-normalized heliocentric radial distance for different values of the (**a**) equilibrium negative-to-positive ion density ratio (*δ*) with fixed *m*_*i*_*/m*_−_ = 1, *T*_*i*_*/T*_*e*_ = 1 and *T*_−_*/T*_*e*_ = 1; (**b**) positive-to-negative ion mass ratio (*m*_*i*_*/m*_−_) with fixed *δ* = 1/1000, *T*_*i*_*/T*_*e*_ = 1 and *T*_−_*/T*_*e*_ = 1; (**c**) positive ion-to-electron temperature ratio (*T*_*i*_*/T*_*e*_) with fixed *δ* = 1/1000, *m*_*i*_*/m*_−_ = 1 and *T*_−_*/T*_*e*_ = 1; and (**d**) negative ion-to-electron temperature ratio (*T*_−_*/T*_*e*_) with fixed *δ* = 1/1000, *m*_*i*_*/m*_−_ = 1 and *T*_*i*_*/T*_*e*_ = 1.

With the aim of portraying the complete steady-state structure of the solar plasma system based on our proposed GES-based model formalism modified with diverse negative ions, at first, the location of the new SSB formation by an exact gravito-electrostatic force-balancing is investigated and characterized. Accordingly, the strength of the self-gravity and electric field is plotted with the Jeans-normalized heliocentric radial distance for different equilibrium parametric variations, such as *δ*, *m*_*i*_*/m*_−_, *T*_*i*_*/T*_*e*_, and *T*_−_*/T*_*e*_ as illustrated in Fig. 2. Here, the spatial grid size used is 0.25. It is found that the modified SSB divides the entire solar plasma volume into a bi-scaled system, bounded (SIP) and unbounded (SWP), separated by the interfacial SSB. This plasma system evolves alongside new quantitative changes parametrically introduced by the negative ions included for the first time.

It is interestingly observed that as the *δ-*value increases, the SSB and hence the SIP volume shrinks with a reduced maximum self-gravity magnitude of the bounded plasma mass. It can be well explained by the shielding nature of the plasma constituents by the opposite polarity species in the solar plasma medium. As *δ* increases, the negative ion density increases in accordance with the average solar plasma quasi-neutrality condition. The negative ions start to take part in the shielding mechanism of the positive ions together with the electrons. The electrons being negligible in size compared to the protons (positive ions), can shield the protons to a great extent. This overall high micro-scale neutrality facilitates self-gravitational condensation, resulting in high self-gravity at the SSB location. This inter-particle shielding between the negative and positive ions (protons) is not as compact as the shielding between the electrons and the positive ions. As a result, the self-gravitational condensation, as *δ* increases, is not as effective as in the lower *δ*-cases due to the presence of effective electrostatic interactions, resulting in low self-gravity in a reduced SSB location in a shrunk SIP volume.

It is noticed that the *δ-*sensitivity of the shrinking nature of the SIP volume, i.e., inward drifting nature of the SSB is high for high *δ*-values. The difference in the inward SSB-drifting nature becomes more prominent towards the high-*δ* region than that in the low-*δ* region. This behaviour indicates that the SSB-location saturates itself to its radial magnitude in the solar plasma system with *δ* = 0, as the negative ion concentration is gradually decreased. So, the influence of the presence of negative ion becomes insignificant as their concentration is lessened.

It is interestingly seen that for the *δ* = 0 case, the SSB is not forming in our case at the 3.5 on the Jeans length scale, as reported previously in the original GES-model picture without negative ions^5,11^. This shows the sensitiveness of our model to the inclusion of the negative ions. Though we impose the *δ* = 0 condition based on average solar plasma behaviours, the negative ions are still available in the solar plasma system, as being seen pictorially in Fig. 12. So, it can be inferred that the presence of the negative ions is responsible for the SSB location shifting to a new radial location *ξ* = 3.75, against the pure GES SSB location at *ξ* = 3.5 on the Jeans length scale^5^, due to the shielding behavioural physics described above. It is found that the SSB-drifting nature is independent of the *m*_*i*_*/m*_−_, *T*_*i*_*/T*_*e*_, and *T*_−_*/T*_*e*_, as clearly depicted in Fig. 2.

**Figure 3:**
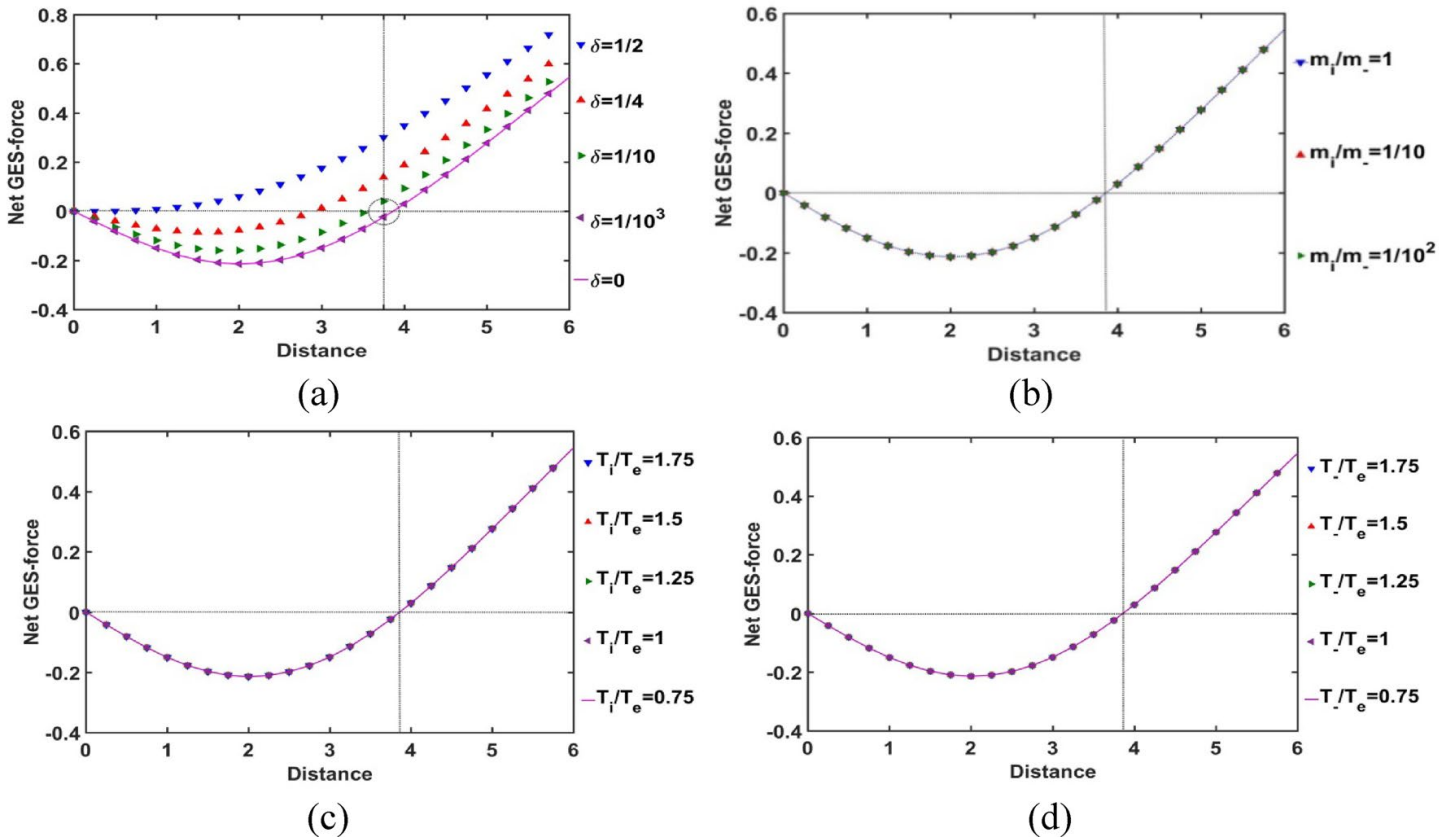
Variation of the net GES-force with the Jeans-normalized heliocentric radial distance for different values of the (**a**) equilibrium negative-to-positive ion density ratio (*δ*) with fixed *m*_*i*_*/m*_−_ = 1, *T*_*i*_*/T*_*e*_ = 1 and *T*_−_*/T*_*e*_ = 1; (**b**) positive-to-negative ion mass ratio (*m*_*i*_*/m*_−_) with fixed *δ* = 1/1000, *T*_*i*_*/T*_*e*_ = 1 and *T*_−_*/T*_*e*_ = 1; (**c**) positive ion-to-electron temperature ratio (*T*_*i*_*/T*_*e*_) with fixed *δ* = 1/1000, *m*_*i*_*/m*_*−*_ = 1 and *T*_−_*/T*_*e*_ = 1; and (**d**) negative ion-to-electron temperature ratio (*T*_−_*/T*_*e*_) with fixed *δ* = 1/100_−__−_0, *m*_*i*_*/m*_−_ = 1 and *T*_*i*_*/T*_*e*_ = 1.

As in Fig. 3, the variation of the net equilibrium GES-force (defined as the algebraic sum of self-gravity and electric field) with the Jeans-normalized heliocentric radial distance variation is depicted for the different indicated values of *δ, m*_*i*_*/m*_−_, *T*_*i*_*/T*_*e*_, and *T*_−_*/T*_*e*_. As already revealed in Fig. 2, the SSB location, here as well, sensitively depends on the variation of this *δ*-parameter only (Fig. 3a), but not so significantly on other considered input parameters (Fig. 3b–d). Interestingly, it is quite in accord with the basic rule of exact gravito-electrostatic force balancing mechanism, thereby leading to the SSB creation, as widely illustrated in the literature^5,10^.

After a methodological identification and characterization of the SSB, separating the SIP and SWP as the entire bi-scaled plasma system as above (Figs. 2 and 3), the investigated key results are described systematically in the following two separate subsections.

### SIP-illustration

The SIP behaviours are analysed by studying the various properties of the bounded solar plasma mass, obtained from the numerical analysis of the combined model equations (Eqs. 11–20), with the initial inputs as presented in Appendix C.

**Figure 4:**
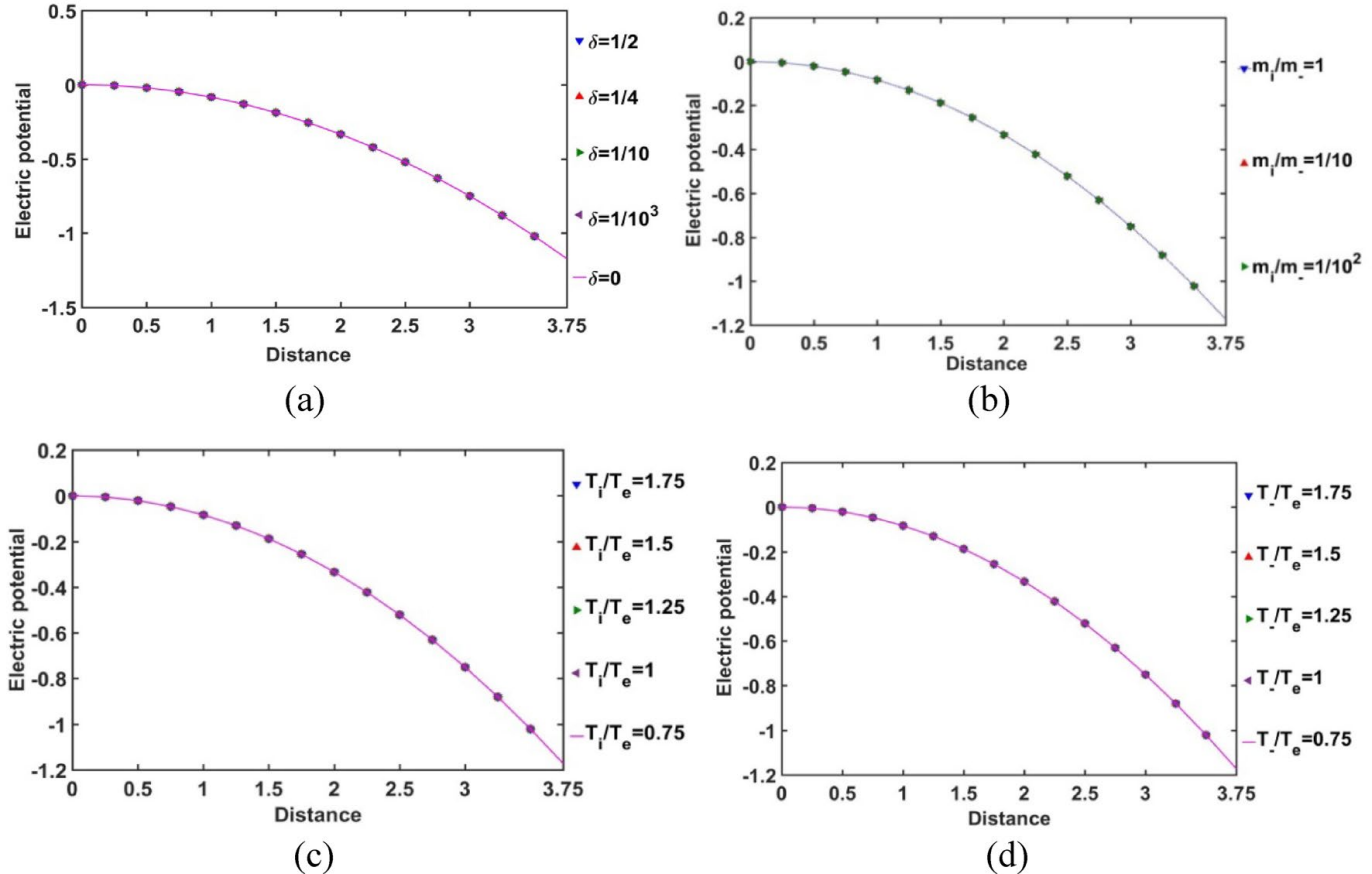
Variation of the normalized electric potential with the Jeans-normalized heliocentric radial distance for different values of the (**a**) equilibrium negative-to-positive ion density ratio (*δ*) with fixed *m*_*i*_*/m*_−_ = 1, *T*_*i*_*/T*_*e*_ = 1 and* T*_−_*/T*_*e*_ = 1; (**b**) positive-to-negative ion mass ratio (*m*_*i*_*/m*_−_) with fixed *δ* = 1/1000, *T*_*i*_*/T*_*e*_ = 1 and *T*_−_*/T*_*e*_ = 1; (**c**) positive ion-to-electron temperature ratio (*T*_*i*_*/T*_*e*_) with fixed *δ* = 1/1000, *m*_*i*_*/m*_−_ = 1 and *T*_−_*/T*_*e*_ = 1; and (**d**) negative ion-to-electron temperature ratio (*T*_−_*/T*_*e*_) with fixed *δ* = 1/1000, *m*_*i*_*/m*_−_ = 1 and *T*_*i*_*/T*_*e*_ = 1.

As depicted in Fig. 4, the profile of the normalized electric potential variation with the Jeans-normalized heliocentric radial distance is obtained for different values of *δ*, *m*_*i*_*/m*_−_, *T*_*i*_*/T*_*e*_ and *T*_−_*/T*_*e*_. It is seen here that the electric potential is independent of the equilibrium negative ion population, mass of the negative ion as compared to the proton mass, and temperature of the positive and negative ions as compared to the electron temperature. The electric potential becomes negligible as the heliocenter is approached. It indicates high material density in the heliocentric region, which causes a significant shielding between the constitutive particles with opposite polarities. Away from the heliocenter outwards, the material density decreases and particle diffusivity increases. As a consequence, the electrostatic polarization effects become more prominent, and so forth. The obtained results on the patterns of the spatial variation of electric potential are, in fact, found to be in a fair agreement with the recently reported thermo-statistically modified realistic GES model description^11^, thereby validating our current analysis.

**Figure 5:**
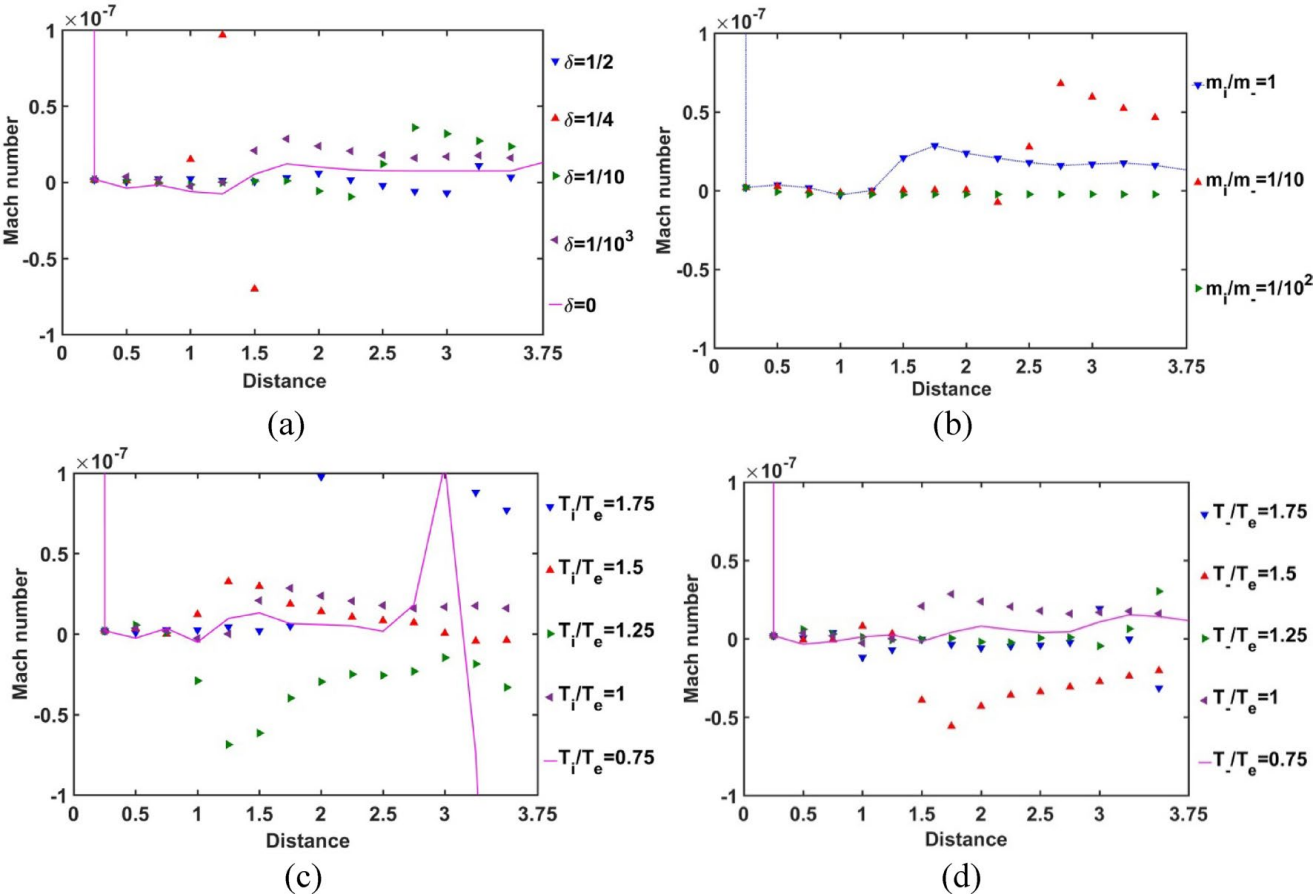
Variation of the SIP Mach number with the Jeans-normalized heliocentric radial distance for different values of the (**a**) equilibrium negative-to-positive ion density ratio (*δ*) with fixed *m*_*i*_*/m*_−_ = 1, *T*_*i*_*/T*_*e*_ = 1 and *T*_−_*/T*_*e*_ = 1; (**b**) positive-to-negative ion mass ratio (*m*_*i*_*/m*_−_) with fixed *δ* = 1/1000, *T*_*i*_*/T*_*e*_ = 1 and *T*_−_*/T*_*e*_ = 1; (**c**) positive ion-to-electron temperature ratio (*T*_*i*_*/T*_*e*_) with fixed *δ* = 1/1000, *m*_*i*_*/m*_−_ = 1 and *T*_−_*/T*_*e*_ = 1; and (**d**) negative ion-to-electron temperature ratio (*T*_−_*/T*_*e*_) with fixed *δ* = 1/1000, *m*_*i*_*/m*_−_ = 1 and *T*_*i*_*/T*_*e*_ = 1.

As depicted in Fig. 5, the spatial profile of the SIP Mach number is obtained for different indicated values of *δ*, *m*_*i*_*/m*_−_, *T*_*i*_*/T*_*e*_ and *T*_−_*/T*_*e*_. It is noticed that the Mach number is very small in the SIP region. It is due to the very high plasma density in the SIP under self-gravitational action. The unidirectional ionic flow is significantly reduced by the inter-species collisions as well as the gravito-electrostatic interactions. The significant fluctuations in the Mach number with various parameters in the SIP are dependent on the radial material density fluctuations of the surrounding SIP medium. So, we can infer from here that the regions with a relatively high Mach number value have a drop in the material density as compared to the rest of the SIP-regions. It is quite in accordance with the basic rule of flux density conservation governed by the equation of continuity (describing the usual density-flow correlation). As a result, for *δ* = 1/4, there lies a rarefied region at *ξ* = 1–1.5 (Fig. 5a); for *m*_*i*_*/m*_−_ = 1/10, a comparatively rarer region appears at *ξ* = 2.5–3.75 (Fig. 5b); for *T*_*i*_*/T*_*e*_ = 0.75, a rarer region forms at *ξ* = 3–3.75 (Fig. 5c), and for *T*_−_*/T*_*e*_ = 1.5, such a low-density region structurizes at *ξ* = 1.5–2 (Fig. 5d), and so forth. It is found throughout that the SIP Mach number at the SSB comes out to be *M*_SSB_ = 1.3 × 10^–8^ (Fig. 5).

**Figure 6:**
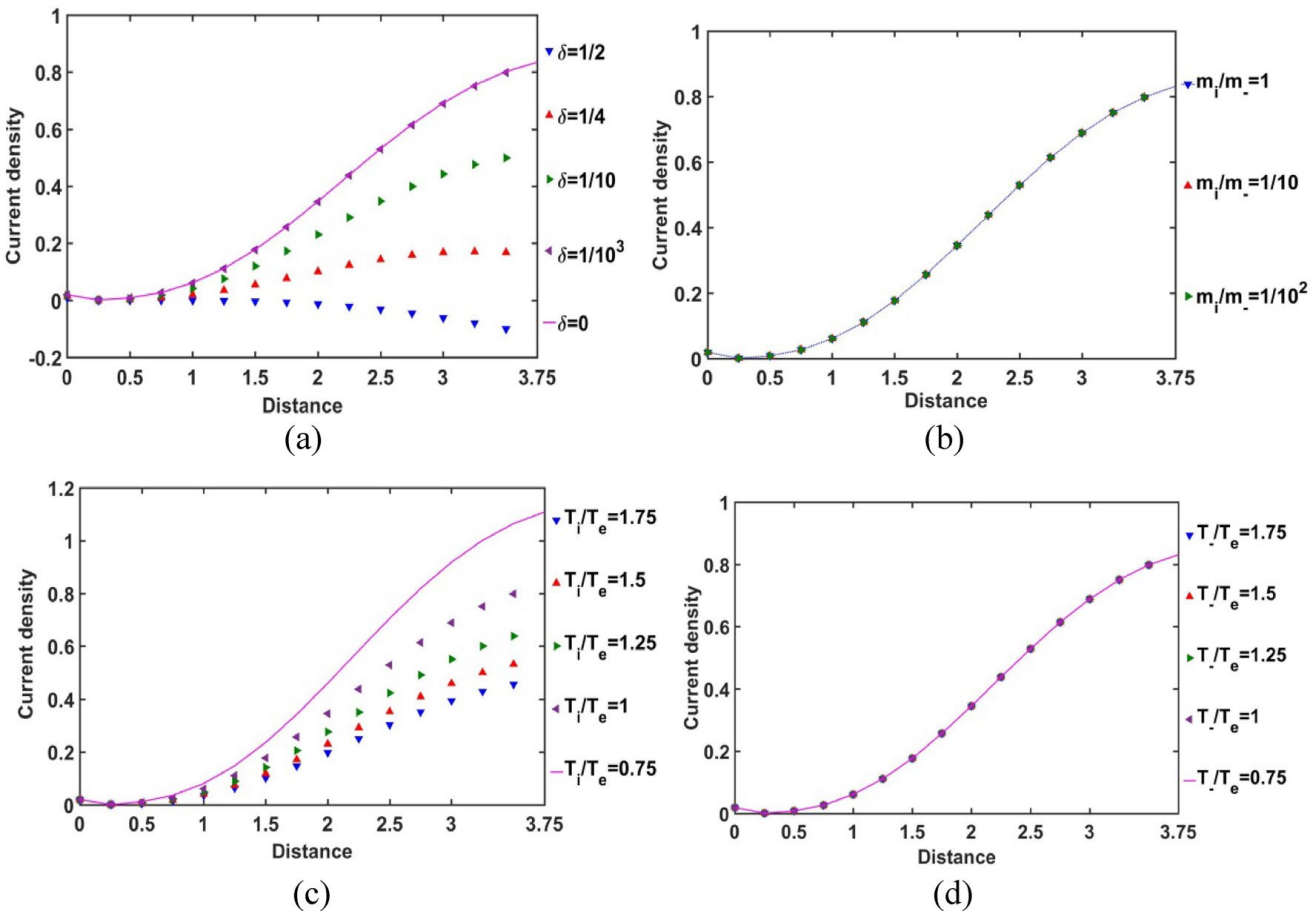
Variation of the SIP current density with the Jeans-normalized heliocentric radial distance for different values of the (**a**) equilibrium negative-to-positive ion density ratio (*δ*) with fixed *m*_*i*_*/m*_−_ = 1, *T*_*i*_*/T*_*e*_ = 1 and *T*_−_*/T*_*e*_ = 1; (**b**) positive-to-negative ion mass ratio (*m*_*i*_*/m*_−_) with fixed *δ* = 1/1000, *T*_*i*_*/T*_*e*_ = 1 and *T*_−_*/T*_*e*_ = 1; (**c**) positive ion-to-electron temperature ratio (*T*_*i*_*/T*_*e*_) with fixed *δ* = 1/1000, *m*_*i*_*/m*_−_ = 1 and *T*_−_*/T*_*e*_ = 1; and (**d**) negative ion-to-electron temperature ratio (*T*_−_*/T*_*e*_) with fixed *δ* = 1/1000, *m*_*i*_*/m*_−_ = 1 and *T*_*i*_*/T*_*e*_ = 1.

In Fig. 6, the Bohm-normalized SIP electric current density variation with the Jeans-normalized heliocentric radial distance is numerically portrayed. It is seen that the current density decreases with an increase in *δ*, and vice-versa (Fig. 6a). It is consequent upon the fact that, as *δ* increases, the negative ion density increases, and vice-versa. So, we can infer that the presence of constitutive negative ions affects the net directional electric charge movement in the SIP medium significantly. It is also seen that the difference in the *δ*-sensitivities of the net electric current density decreases with an increase in *δ*. So, it can be inferred herewith that the current density saturates itself towards the SIP with the maximum* δ*-value as and when the *δ-*value increases. The relative mass of the positive ions with respect to the negative ions does not affect the net current density in the SIP (Fig. 6b). The SIP current density is sensitive to the relative temperature of the positive ion with respect to the electron temperature. As the ionic temperature increases, the current density decreases, and vice-versa. It implicates that the high thermo-mechanical energy of the protons affects the net charge directional flow; the relatively cold positive ions result in a relatively high electric current. The difference in *T*_*i*_*/T*_*e*_-sensitivities of the net SIP current density decreases with an increase in the *T*_*i*_*/T*_*e*_-value (Fig. 6c). So, the net electric current density saturates itself to its saturation value in the plasma medium of high ionic temperature with respect to the electronic temperature as the *T*_*i*_*/T*_*e*_-value increases. However, interestingly, the temperature and hence, the kinetic energy of the considered negative ions does not influence the net charge directional flow contributed by the background existing positive ions on the SIP scale (Fig. 6d). It enables us to infer here that, due to the low concentration of the negative ionic species (in corroboration with Fig. 12), the negative ionic species are unable to affect the net electric current density as either their mass (Fig. 6b) or their temperature (Fig. 6d) are varied in the judicious ranges as considered herein.

**Figure 7:**
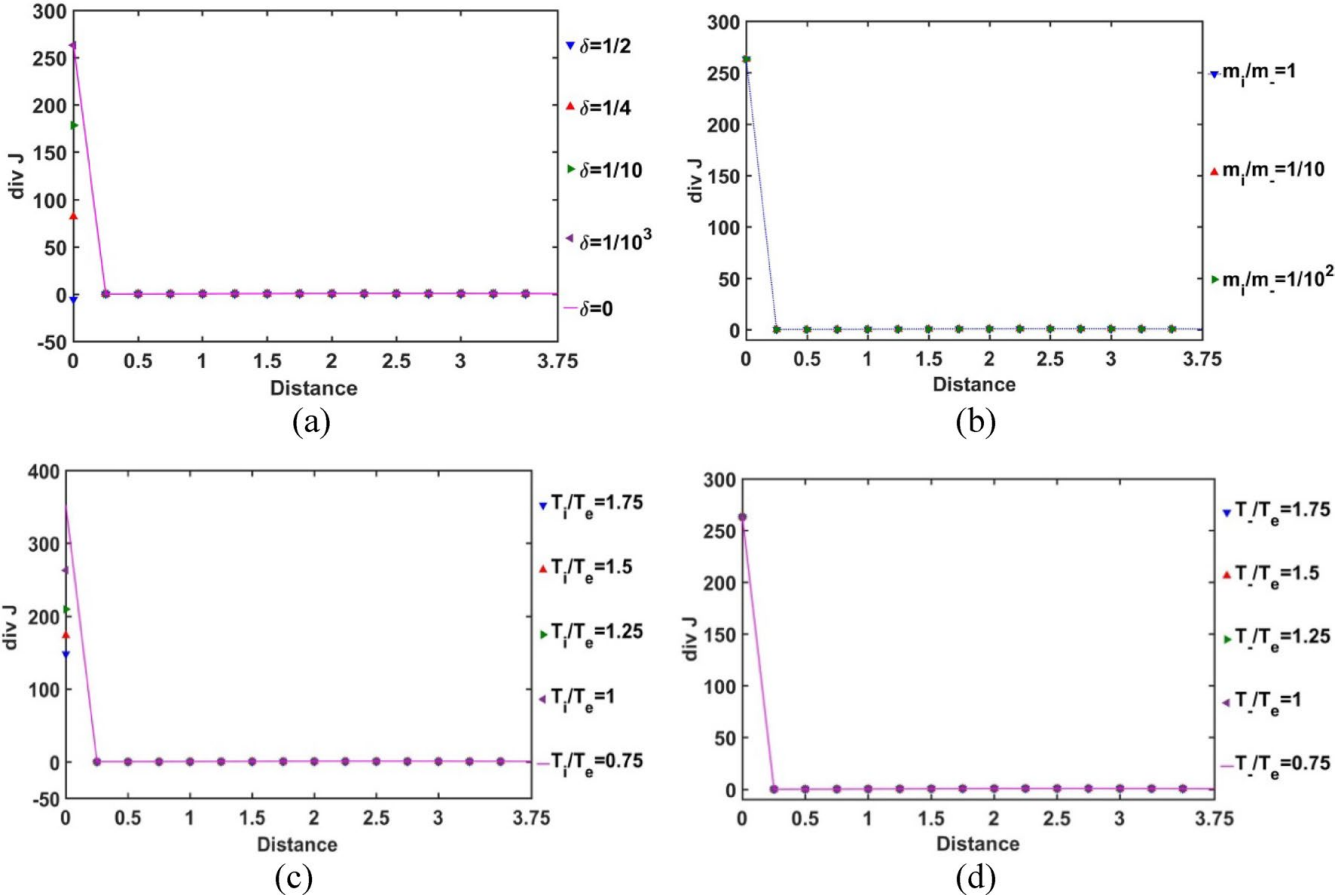
Variation of the divergence of the SIP electric current density (div *J*) with the Jeans-normalized heliocentric radial distance for different values of the (**a**) equilibrium negative-to-positive ion density ratio (*δ*) with fixed *m*_*i*_*/m*_−_ = 1, *T*_*i*_*/T*_*e*_ = 1 and *T*_−_*/T*_*e*_ = 1; (**b**) positive-to-negative ion mass ratio (*m*_*i*_*/m*_−_) with fixed *δ* = 1/1000, *T*_*i*_*/T*_*e*_ = 1 and *T*_−_*/T*_*e*_ = 1; (**c**) positive ion-to-electron temperature ratio (*T*_*i*_*/T*_*e*_) with fixed *δ* = 1/1000, *m*_*i*_*/m*_−_ = 1 and *T*_−_*/T*_*e*_ = 1; and (**d**) negative ion-to-electron temperature ratio (*T*_−_*/T*_*e*_) with fixed *δ* = 1/1000, *m*_*i*_*/m*_−_ = 1 and *T*_*i*_*/T*_*e*_ = 1.

In order for exploring the conservative nature of the SIP electric current density, the divergence of the Bohm-normalized SIP current density variation with the Jeans-normalized heliocentric radial distance is depicted in Fig. 7. It is found that the electric current is well conserved throughout the equilibrium SIP, except near the heliocentric region (up to *ξ *≈ 0.25); in particular, no asymptotic variation is noted beyond it (Fig. 7). There exists no local source or sink to affect the net charge production and its directional flow in the SIP, except in the near-heliocentric regions (*ξ* = 0–0.25). The finite non-zero positive divergence of the net electric current density in the near-heliocentric regions is ascribable to the intense self-gravity action localized in the dense regions, unlike that found in the far-heliocentric regions (*ξ* > 0.25). The conservative nature of the effective electric current density is interestingly found to be independent of the any of the parametric variations, such as *δ*, *m*_*i*_*/m*_−_, *T*_*i*_*/T*_*e*_, and *T*_−_*/T*_*e*_ (as evident in Fig. 7).

**Figure 8:**
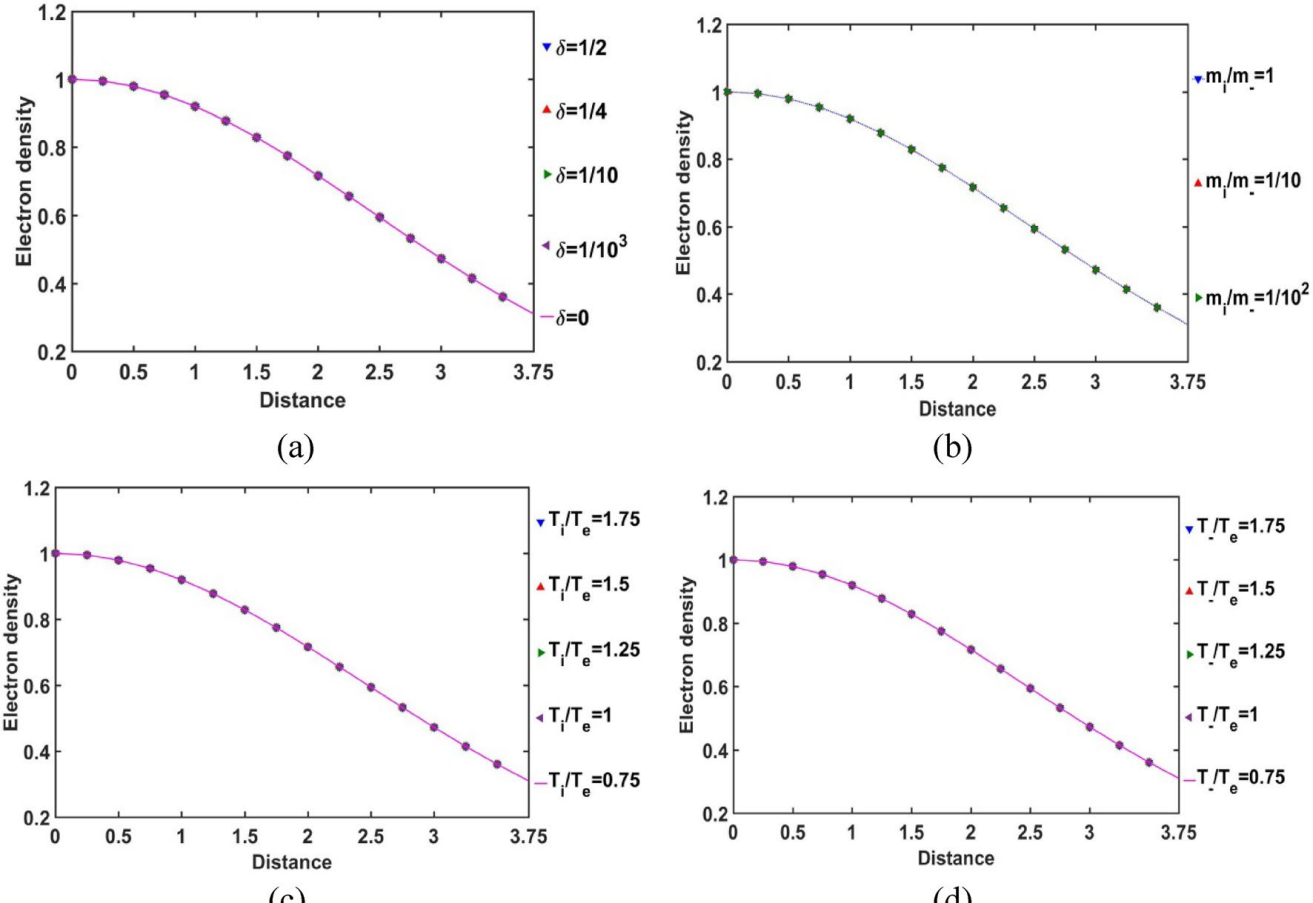
Variation of the normalized SIP electron population density with the Jeans-normalized heliocentric radial distance for different values of the (**a**) equilibrium negative-to-positive ion density ratio (*δ*) with fixed *m*_*i*_*/m*_−_ = 1, *T*_*i*_*/T*_*e*_ = 1 and *T*_−_*/T*_*e*_ = 1; (**b**) positive-to-negative ion mass ratio (*m*_*i*_*/m*_−_) with fixed *δ* = 1/1000, *T*_*i*_*/T*_*e*_ = 1 and *T*_−_*/T*_*e*_ = 1; (**c**) positive ion-to-electron temperature ratio (*T*_*i*_*/T*_*e*_) with fixed *δ* = 1/1000, *m*_*i*_*/m*_−_ = 1 and *T*_−_*/T*_*e*_ = 1; and (**d**) negative ion-to-electron temperature ratio (*T*_−_*/T*_*e*_) with fixed *δ* = 1/1000, *m*_*i*_*/m*_−_ = 1 and *T*_*i*_*/T*_*e*_ = 1.

The radial variation of the normalized electron population density in the SIP according to the current GES-model equations is portrayed in Fig. 8. It is found that the electron population density is independent of any of the parameters: *δ*, *m*_*i*_*/m*_−_, *T*_*i*_*/T*_*e*_ and *T*_−_*/T*_*e*_. The electrons reside mostly in the core and their population density gradually decreases away from the heliocenter. This electron population behaviour is in accordance with the electric potential variation in the SIP (Fig. 4). Away from the heliocenter outwards, the magnitude of the electric potential increases (negative in sense), and hence, the electron population density decreases towards the SSB accordingly. This is quite in accordance with the previous GES-based model predictions^5,11^.

**Figure 9:**
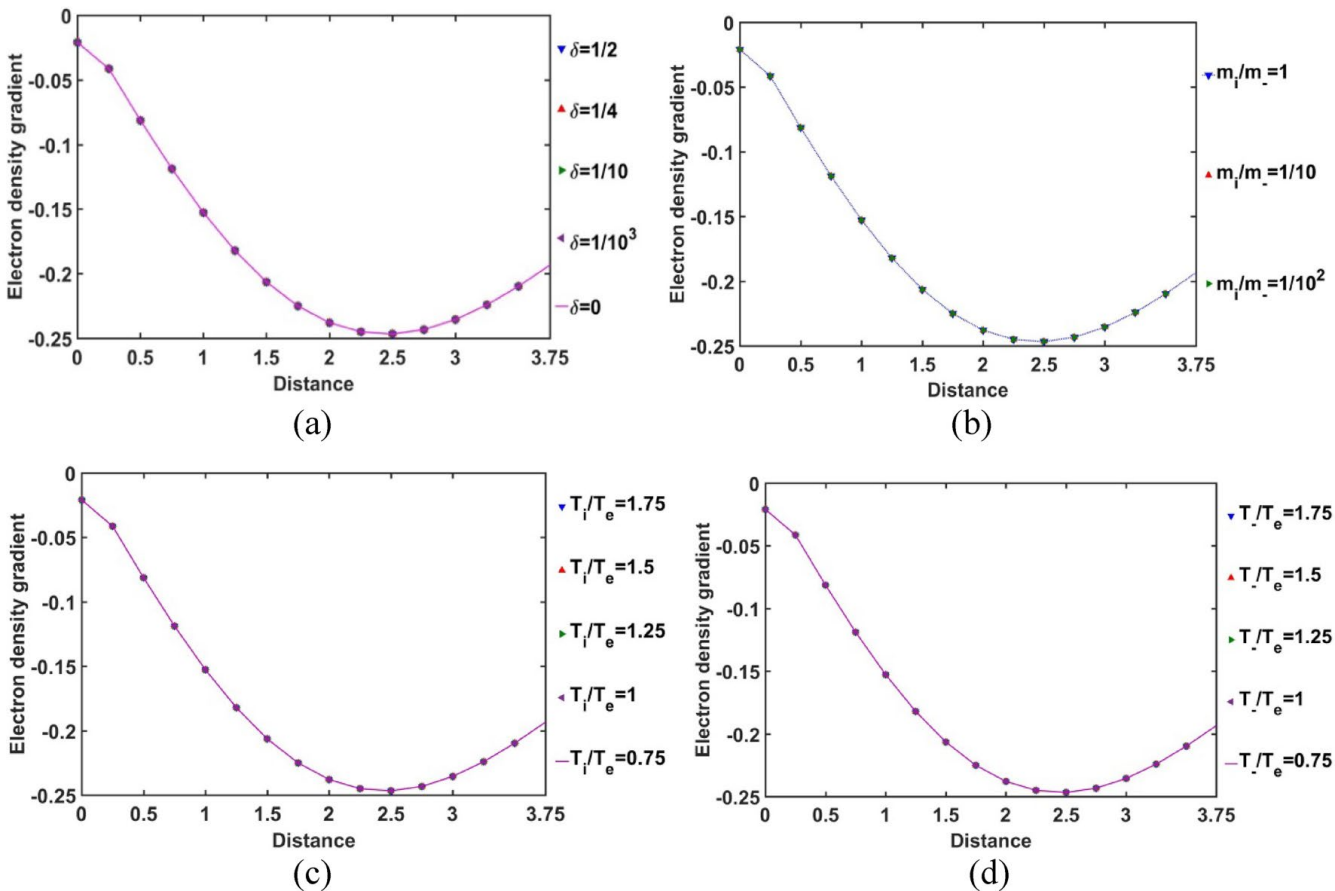
Variation of the normalized SIP electron population density gradient with the Jeans-normalized heliocentric radial distance for different values of the (**a**) equilibrium negative-to-positive ion density ratio (*δ*) with fixed *m*_*i*_*/m*_−_ = 1, *T*_*i*_*/T*_*e*_ = 1 and *T*_−_*/T*_*e*_ = 1; (**b**) positive-to-negative ion mass ratio (*m*_*i*_*/m*_−_) with fixed *δ* = 1/1000, *T*_*i*_*/T*_*e*_ = 1 and *T*_−_*/T*_*e*_ = 1; (**c**) positive ion-to-electron temperature ratio (*T*_*i*_*/T*_*e*_) with fixed *δ* = 1/1000, *m*_*i*_*/m*_−_ = 1 and *T*_−_*/T*_*e*_ = 1; and (**d**) negative ion-to-electron temperature ratio (*T*_−_*/T*_*e*_) with fixed *δ* = 1/1000, *m*_*i*_*/m*_−_ = 1 and *T*_*i*_*/T*_*e*_ = 1.

In Fig. 9, the radial variation of the gradient of the normalized SIP electron population density is illustrated for different considered values of *δ* (Fig. 9a), *m*_*i*_*/m*_−_ (Fig. 9b), *T*_*i*_*/T*_*e*_ (Fig. 9c) and *T*_−_*/T*_*e*_ (Fig. 9d). The electron density gradient is found to be negative throughout the entire SIP region. But, it reaches to its minimum at *ξ* = 2.5 and then, keeps on increasing slightly towards the SSB. This density gradient behaviour implicates that the electron population density goes on decreasing very sharply (i.e., highly non-uniform radial distribution) away from the heliocenter up to *ξ* = 2.5. This electronic distribution non-uniformity slightly decreases thereafter towards the SSB. Clearly, it has revealed a unique electronic population re-structurization of the SIP modified by the constitutive negative ion distribution for the first time in the GES picture.

**Figure 10:**
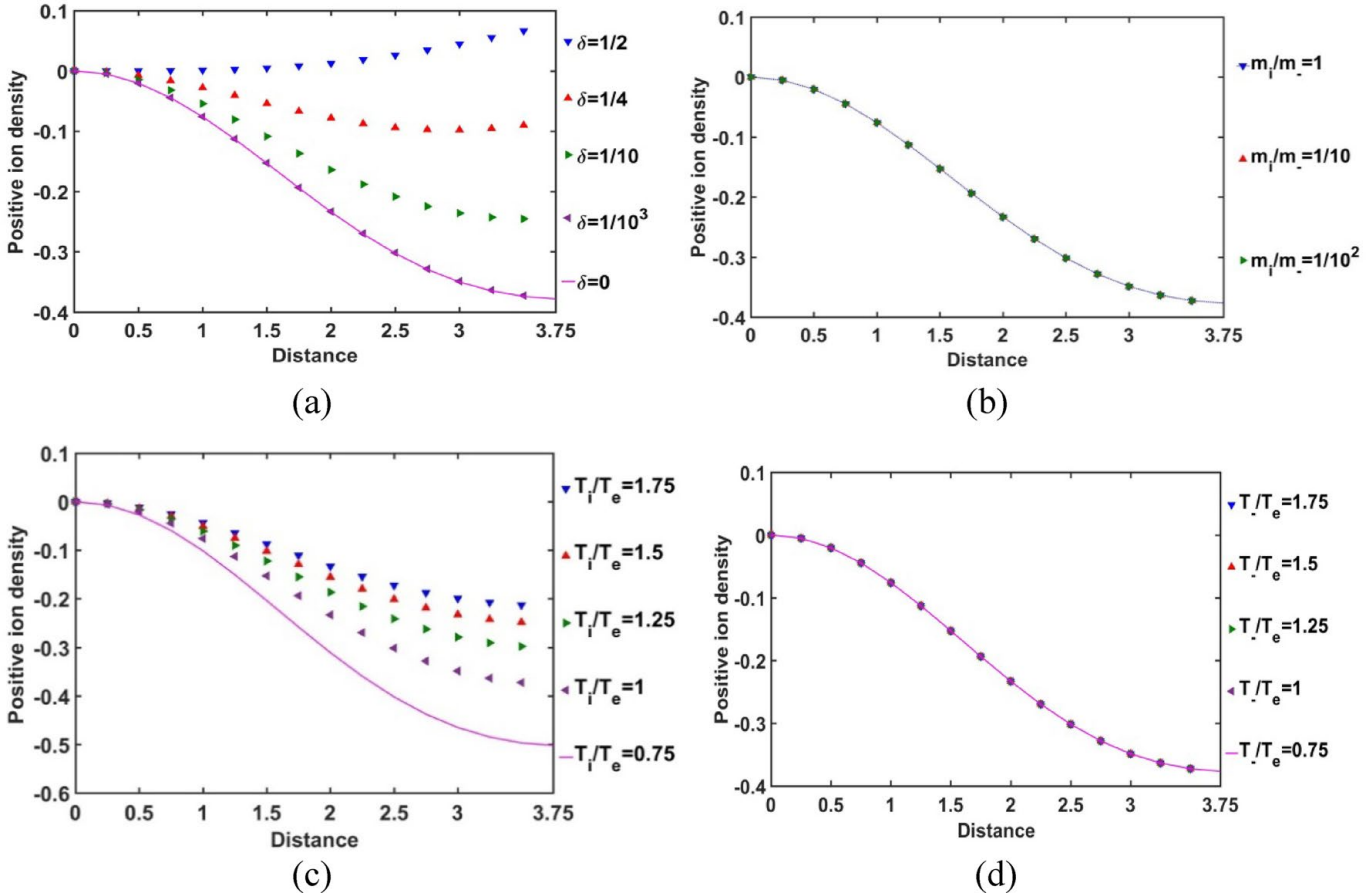
Variation of the SIP positive ion population density with the Jeans-normalized heliocentric radial distance for different values of the (**a**) equilibrium negative-to-positive ion density ratio (*δ*) with fixed *m*_*i*_*/m*_−_ = 1, *T*_*i*_*/T*_*e*_ = 1 and *T*_−_*/T*_*e*_ = 1; (**b**) positive-to-negative ion mass ratio (*m*_*i*_*/m*_−_) with fixed *δ* = 1/1000, *T*_*i*_*/T*_*e*_ = 1 and *T*_−_*/T*_*e*_ = 1; (**c**) positive ion-to-electron temperature ratio (*T*_*i*_*/T*_*e*_) with fixed *δ* = 1/1000, *m*_*i*_*/m*_−_ = 1 and *T*_−_*/T*_*e*_ = 1; and (**d**) negative ion-to-electron temperature ratio (*T*_−_*/T*_*e*_) with fixed *δ* = 1/1000, *m*_*i*_*/m*_−_ = 1 and *T*_*i*_*/T*_*e*_ = 1.

In Fig. 10, the spatial variation of the normalized positive ion population density in the SIP in the radial direction is presented for different relevant parametric variations. It is revealed herein that the normalized positive ion density is negative in the SIP, except for high *δ*-values. The negative value of the normalized positive ion population density, however, actually indicates the deficit of positive ion from the average solar plasma density in the equilibrium configuration. As a result, we can infer that the pre-existing quasi-neutrality of the SIP deviates significantly from the normal GES-based solar plasma configuration because of the perturbative negative ions considered afresh. It is further speculating that this positive ionic density variation is sensitive to the equilibrium negative ion concentration (Fig. 10a). As the *δ-*value increases, the negative ion density increases and vice-versa. But the electron density remains the same (Fig. 8a). We further see that, with an increase in *δ*, the normalized positive ion population density deviation in the negative direction decreases from the average plasma density at the equilibrium, which shows that the positive ion density increases in the SIP. The difference in the *δ*-sensitivities of the positive ion population increases with a decrease in* δ* and vice-versa. So, the SIP positive ion population saturates itself towards the SIP with the maximum* δ*-value with an increase in *δ*.

The SIP positive ion density is insensitive to their relative mass with respect to the negative ions (Fig. 10b). The relative positive ion temperature with respect to the electron temperature influences the positive ion density commensurably (Fig. 10c). It implies that high collision rate and kinetic energy of the positive ions help in their production in the SIP medium. This behaviour saturates itself to high positive ionic temperature SIP-scenario as the difference in the *T*_*i*_*/T*_*e*_-sensitivities goes on decreasing with increasing *T*_*i*_*/T*_*e*_-value. But the negative ion collisional rate and their kinetic energy do not affect the positive ion population (Fig. 10d).

**Figure 11:**
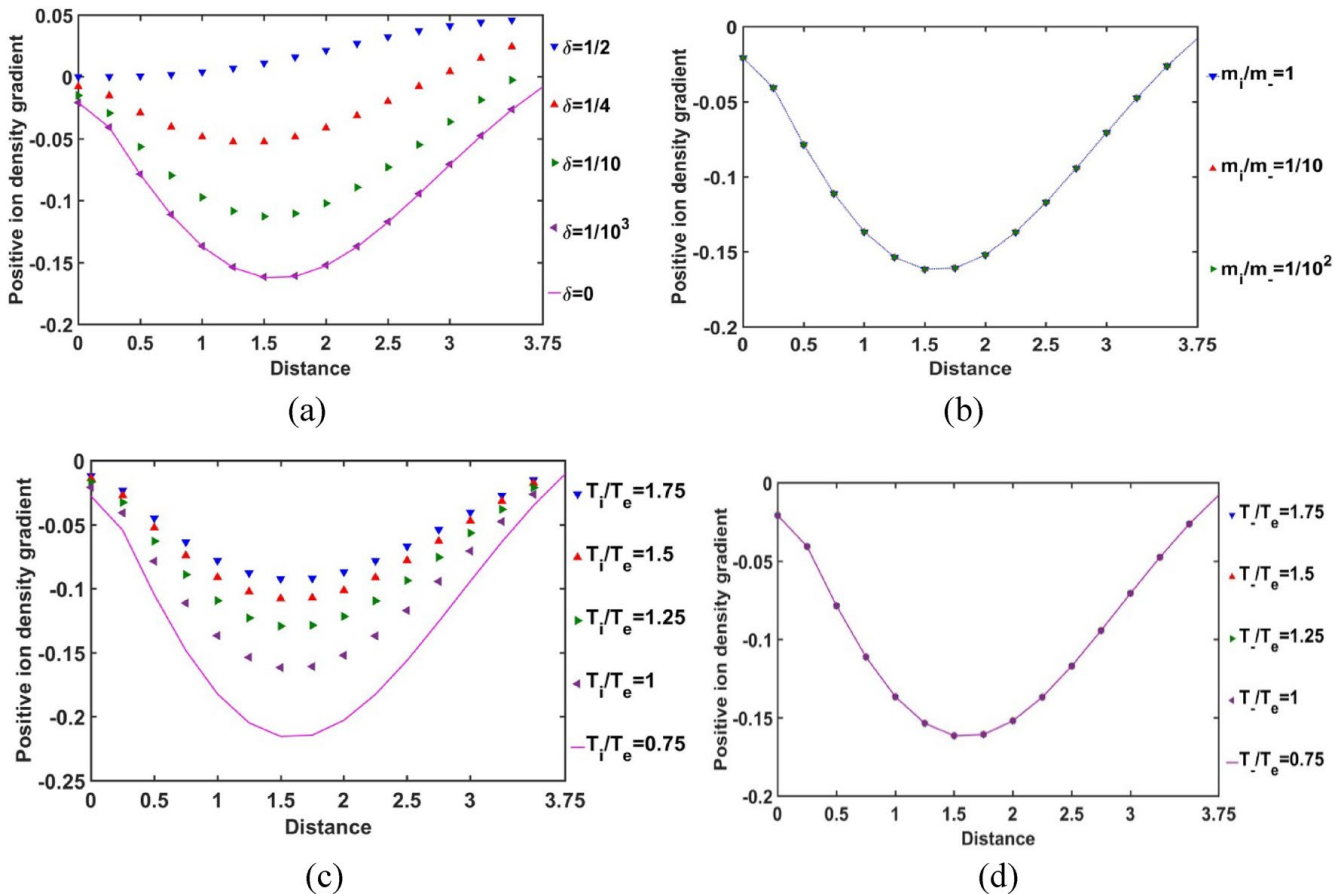
Variation of the normalized SIP positive ion population density gradient with the Jeans-normalized heliocentric radial distance for different values of the (**a**) equilibrium negative-to-positive ion density ratio (*δ*) with fixed *m*_*i*_*/m*_−_ = 1, *T*_*i*_*/T*_*e*_ = 1 and *T*_−_*/T*_*e*_ = 1; (**b**) positive-to-negative ion mass ratio (*m*_*i*_*/m*_−_) with fixed *δ* = 1/1000, *T*_*i*_*/T*_*e*_ = 1 and *T*_−_*/T*_*e*_ = 1; (**c**) positive ion-to-electron temperature ratio (*T*_*i*_*/T*_*e*_) with fixed *δ* = 1/1000, *m*_*i*_*/m*_−_ = 1 and *T*_−_*/T*_*e*_ = 1; and (**d**) negative ion-to-electron temperature ratio (*T*_−_*/T*_*e*_) with fixed *δ* = 1/1000, *m*_*i*_*/m*_−_ = 1 and *T*_*i*_*/T*_*e*_ = 1.

In Fig. 11, the radial variation of the normalized SIP positive ion density gradient is shown for various *δ*, *m*_*i*_*/m*_−_, *T*_*i*_*/T*_*e*_ and *T*_−_*/T*_*e*_. It is seen that the positive ion density gradient decreases with a decrease in* δ* and vice-versa (Fig. 11a). It depicts that, with a decrease in *δ*, the positive ion density non-uniformity increases and vice-versa. This gradient sharply falls from the heliocenter to *ξ* = 1.5, which is the location of the maximum non-uniformity; and then keeps on increasing steeply towards the SSB. It is also interestingly found that, the sensitiveness of this non-uniformity on *δ* becomes more prominent in the radial mid-SIP region than that in the heliocentric and near-SSB regions in the SIP. The difference in the *δ*-sensitivities of the positive ion density gradient decreases with an increase in* δ* and vice-versa*.* It shows that the increasing negative ion concentration saturates the positive ion density gradient towards the maximum* δ*-value in the SIP picture (Fig. 11a). However, no such sensitive variations are speculated in the case of variation in the positive-to-negative ion mass ratio (Fig. 11b). This density gradient behaviour is quite in correlation and consistency with Fig. 10b as already explained above.

As depicted in Fig. 11c, we see that the positive ion density gradient decreases with a decrease in the positive ion temperature with respect to the electronic temperature and vice-versa. For a high positive ionic temperature, their population density uniformity increases in the SIP medium. This density behaviour may be attributable to the high kinetic energy and collision rate of the positive ions that may facilitate in their rapid production in the SIP medium causing high population density uniformity (Fig. 10c). The positive ion density gradient saturates itself towards the SIP configuration with a high positive ion temperature, as the difference in the *T*_*i*_*/T*_*e*_-sensitivities goes on decreasing with an increase in the *T*_*i*_*/T*_*e*_-value. It is quite in accordance with the basic physical insights as already discussed in case of Fig. 10c; and so forth. This spatial variation of the positive ionic density gradient is quite insensitive to the negative ion-to-electron temperature ratio, as clearly evident from Fig. 11d, and so forth. This behaviour is again quite in corroboration with that found in Fig. 10d.

**Figure 12:**
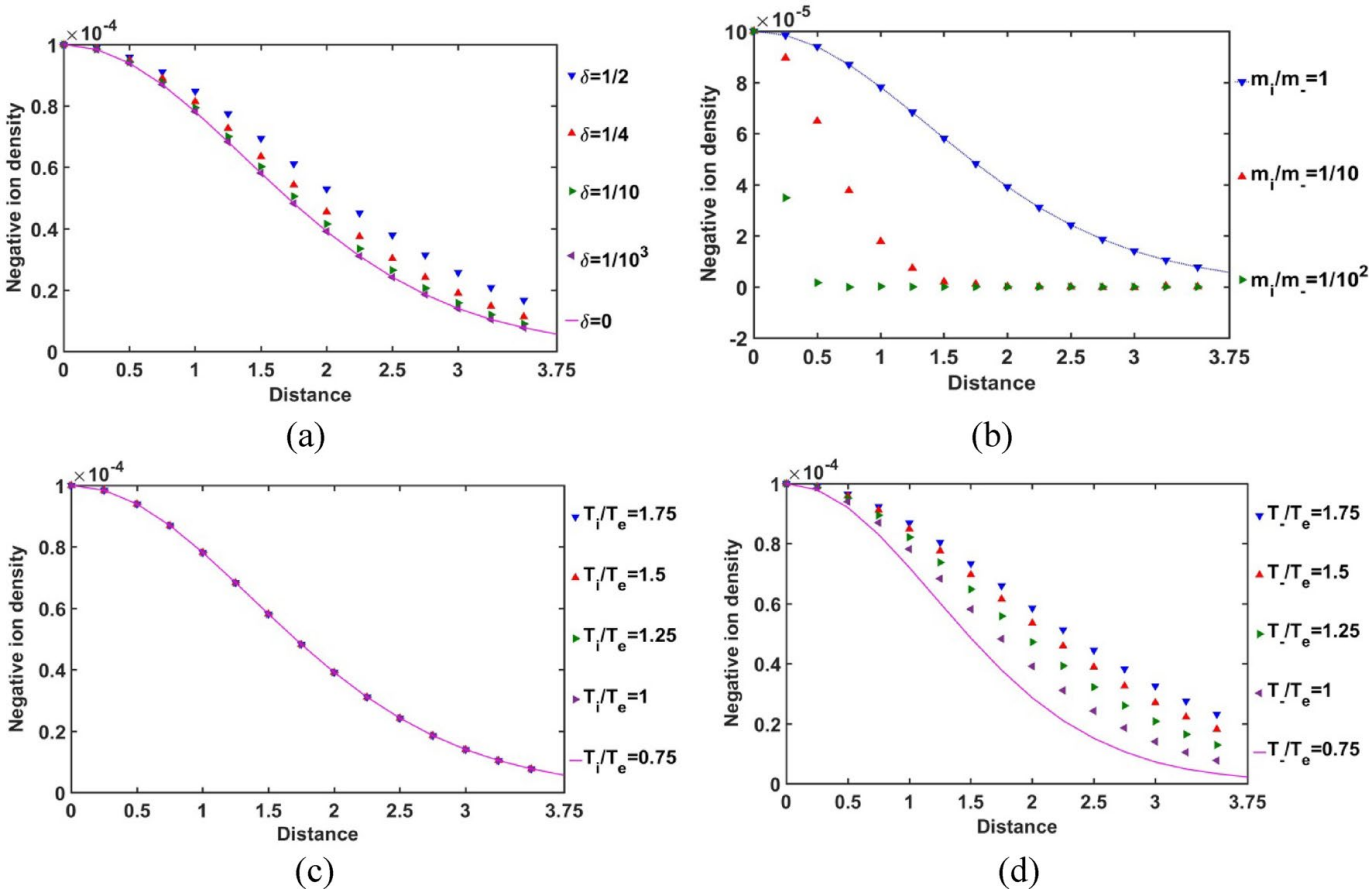
Variation of the SIP negative ion population density with the Jeans-normalized heliocentric radial distance for different values of the (**a**) equilibrium negative-to-positive ion density ratio (*δ*) with fixed *m*_*i*_*/m*_−_ = 1, *T*_*i*_*/T*_*e*_ = 1 and *T*_−_*/T*_*e*_ = 1; (**b**) positive-to-negative ion mass ratio (*m*_*i*_*/m*_−_) with fixed *δ* = 1/1000, *T*_*i*_*/T*_*e*_ = 1 and *T*_−_*/T*_*e*_ = 1; (**c**) positive ion-to-electron temperature ratio (*T*_*i*_*/T*_*e*_) with fixed *δ* = 1/1000, *m*_*i*_*/m*_−_ = 1 and *T*_−_*/T*_*e*_ = 1; and (**d**) negative ion-to-electron temperature ratio (*T*_−_*/T*_*e*_) with fixed *δ* = 1/1000, *m*_*i*_*/m*_−_ = 1 and *T*_*i*_*/T*_*e*_ = 1.

The radial variations of the normalized SIP negative ion population density for *δ*, *m*_*i*_*/m*_−_, *T*_*i*_*/T*_*e*_ and *T*_−_*/T*_*e*_ –variations are graphically depicted in Fig. 12. It is found that the negative ion density in the SIP medium increases with an increase in *δ* and vice-versa (Fig. 12a). We interestingly notice that some residual negative ions are still present in the SIP even for the *δ* = 0 case. These residual negative ionic effects may be ascribable to the diverse cosmic non-ideality influences causing local ionization, recombination, etc.^23^ The difference in the *δ*-sensitivities of the negative ion population density decreases with a decrease in the *δ*-value. So, the negative ion population saturates itself in the SIP with decrease in *δ* to the *δ* = 0 SIP scenarios.

It is furthermore found that the negative ion population is highly sensitive to the positive-to-negative ion mass ratio (Fig. 12b). With an increase in the negative ionic mass, their population density falls rapidly in the SIP. Hence, it is hereby revealed that the SIP medium is not favourable for the heavy clustered negative ion formation, for the first time. It is worth mentioning here that this negative ion population behaviour is following the observational evidence that the hydrogen ion (H^-^) accounts for the large part of the continuous absorption of the solar atmosphere. However, other heavier negative ions have been detected later with advancement in the spectrophotometric analytical techniques, as already mentioned earlier^14,15^. Hence, this match between the current theoretical findings by us and the previous observational scenarios by others enhances the relevance and reliability of our present investigation forward.

The negative ion population density is insensitive to the positive ion-to-electron temperature ratio (Fig. 12c). So, the high positive ionic temperature and hence, collision does not influence in the production of negative ions in the SIP.

Besides, the negative ion population density is influenced significantly by the negative ion-to-electron temperature ratio effectively (Fig. 12d). So, high negative ionic temperature and hence, high kinetic energy and collision help in the generation of the negative ionic species in the SIP. It is noticed that the difference in the* T*_−_*/T*_*e*_-sensitivities decreases with an increase in the *T*_−_*/T*_*e*_-value and vice-versa. The negative ion density saturates itself towards high temperature SIP-scenarios with an increase in their temperature with respect to the electronic temperature. This *T*_−_*/T*_*e*_-sensitivity becomes more prominent away from the heliocenter towards the SSB (Fig. 12d).

**Figure 13:**
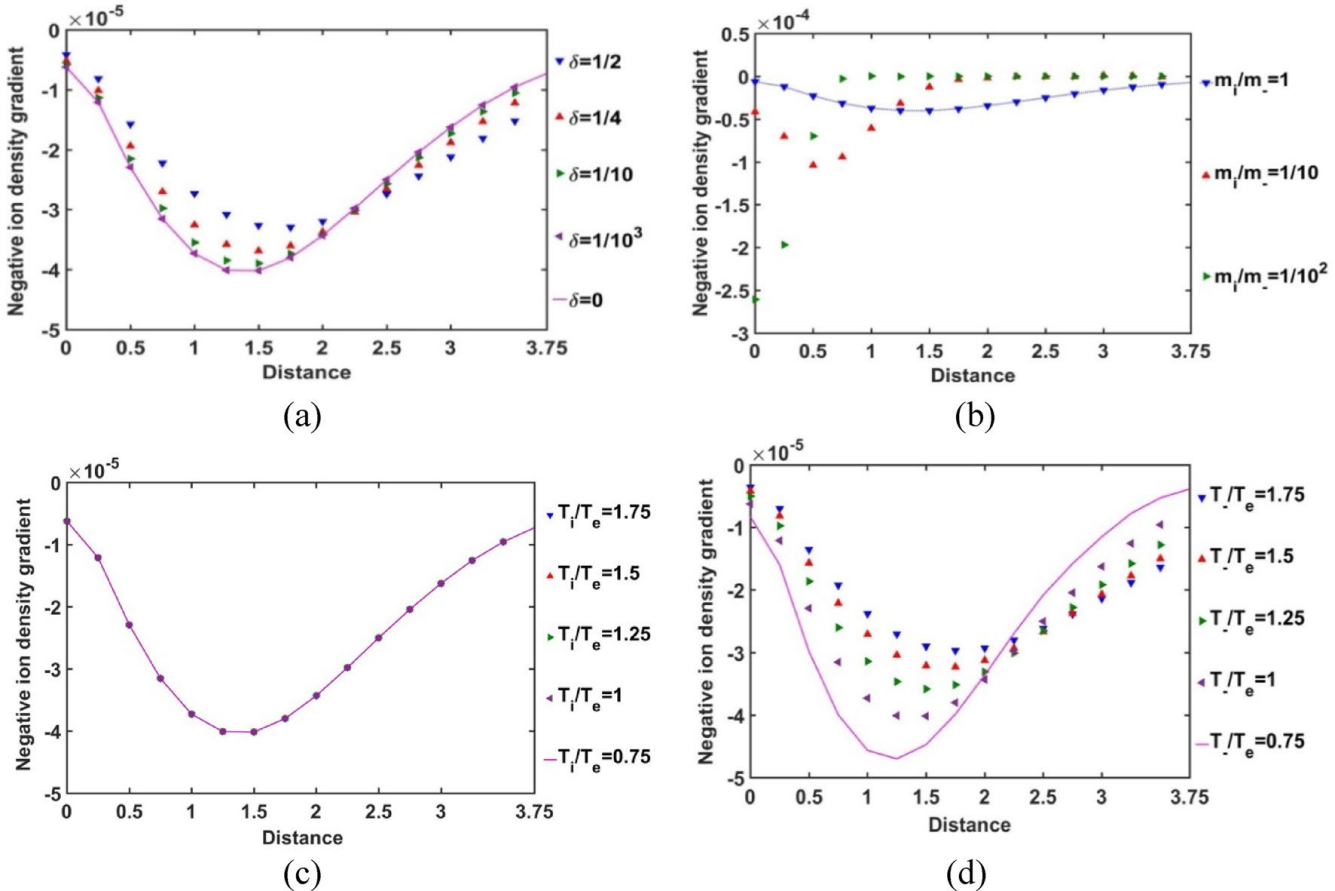
Variation of the normalized SIP negative ion population density gradient with the Jeans-normalized heliocentric radial distance for different values of the (**a**) equilibrium negative-to-positive ion density ratio (*δ*) with fixed *m*_*i*_*/m*_−_ = 1, *T*_*i*_*/T*_*e*_ = 1 and *T*_−_*/T*_*e*_ = 1; (**b**) positive-to-negative ion mass ratio (*m*_*i*_*/m*_−_) with fixed *δ* = 1/1000, *T*_*i*_*/T*_*e*_ = 1 and *T*_−_*/T*_*e*_ = 1; (**c**) positive ion-to-electron temperature ratio (*T*_*i*_*/T*_*e*_) with fixed *δ* = 1/1000, *m*_*i*_*/m*_−_ = 1 and *T*_−_*/T*_*e*_ = 1; and (**d**) negative ion-to-electron temperature ratio (*T*_−_*/T*_*e*_) with fixed *δ* = 1/1000, *m*_*i*_*/m*_−_ = 1 and *T*_*i*_*/T*_*e*_ = 1.

In Fig. 13, the variation of the normalized SIP negative ion density gradient with the Jeans-normalized heliocentric radial distance is shown for different values of *δ*, *m*_*i*_*/m*_−_, *T*_*i*_*/T*_*e*_ and *T*_−_*/T*_*e*_. It is seen that the uniformity in the negative ion population density decreases with an increase in the radial distance up to *ξ* ≈ 1.5 and then keeps on increasing subsequently towards the SSB for various *δ*-values (Fig. 13a). It is interestingly seen that there lies a radial location between *ξ* = 2–2.5, where this density gradient becomes the same irrespective of the *δ-*values, and the variation trend becomes reverse afterwards to the SSB. So, there appears a *δ-*insensitive location, which may be termed as a trans-critical point, for the negative ion population non-uniformity in the SIP. The *δ-*sensitivity of the negative ion density gradient goes on decreasing with a decrease in the *δ-*value. Therefore, this density gradient saturates itself towards the *δ* = 0 case as *δ* decreases in the SIP configuration.

It is furthermore seen that the non-uniformity in the negative ion population density in the SIP is higher for the heavier negative ions; particularly, in the near-heliocentric region, and becomes insignificant away from the heliocenter (Fig. 13b). This is because of the fact that the negative ion population in such off-centric region becomes considerably negligible as seen previously in Fig. 12b. Thus, the negative ion density gradient evolves in accordance with the usual plasma collective interaction processes as already discussed above. This spatial variation of the density gradient is insensitive to the positive ion-to-electron temperature ratio, as evident from Fig. 13c. This behaviour is again quite in fair conformity with Fig. 12c.

It is speculated from Fig. 13d that the negative ion density gradient decreases with a decrease in the negative ion temperature with respect to the electronic temperature. For high negative ionic temperature, their population density uniformity increases in the SIP medium. This behaviour may be attributable to the kinetic energy and high collision rate of the negative ions that may help in their production in the SIP medium. As a consequence, it causes high population uniformities in the SIP (Fig. 12d). The negative ion density gradient saturates itself towards the SIP-picture with high negative ion temperature, as the difference in the *T*_−_*/T*_*e*_-sensitivities goes on decreasing with an increase in the *T*_−_*/T*_*e*_ – value. This trend is evidently in accordance with the basic physical mechanisms already stated in Fig. 12d. It is noteworthy that the trans-critical point on the *T*_−_*/T*_*e*_-sensitivity here (Fig. 13d), similar to that on the *δ*-sensitivity (Fig. 13a), lies in the same region bounded between the radial points at about *ξ* = 2–2.5.

**Figure 14:**
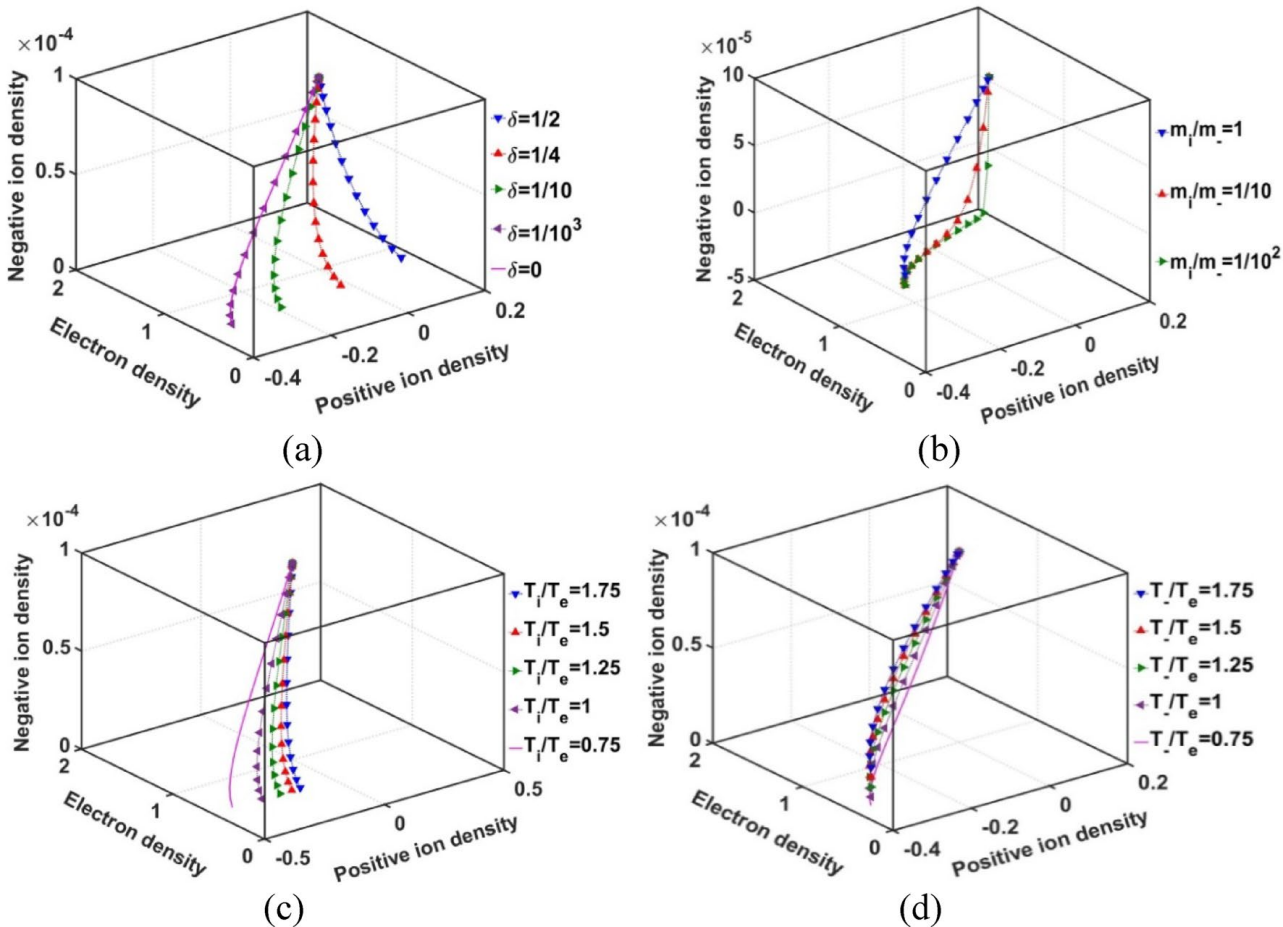
Variation of the negative ion population density with the positive ion and electron population densities in the SIP for different values of the (**a**) equilibrium negative-to-positive ion density ratio (*δ*) with fixed *m*_*i*_*/m*_−_ = 1, *T*_*i*_*/T*_*e*_ = 1 and *T*_−_*/T*_*e*_ = 1; (**b**) positive-to-negative ion mass ratio (*m*_*i*_*/m*_−_) with fixed *δ* = 1/1000, *T*_*i*_*/T*_*e*_ = 1 and *T*_−_*/T*_*e*_ = 1; (**c**) positive ion-to-electron temperature ratio (*T*_*i*_*/T*_*e*_) with fixed *δ* = 1/1000, *m*_*i*_*/m*_−_ = 1 and *T*_−_*/T*_*e*_ = 1; and (**d**) negative ion-to-electron temperature ratio (*T*_−_*/T*_*e*_) with fixed *δ* = 1/1000, *m*_*i*_*/m*_−_ = 1 and *T*_*i*_*/T*_*e*_ = 1.

In Fig. 14, we depict the normalized population density profile of the SIP constituent species in a conjoint pattern. It is interestingly noticed that the profile of the negative ion population density with variation of the electronic and positive ionic population density follows a particular trail for each* δ* to meet the maximum value of the negative ionic density. This maximum density value is found to be the same irrespective of the *δ*-values (Fig. 14a). As already explained before (Fig. 12a), we can identify that the vertex of the trails meaning the negative ion population density at the heliocenter. The density declining trend along the trails shows the population of the constituents as seen by an observer from the heliocenter towards the SSB. The difference in the *δ*-sensitivities of the trailing patterns becomes more prominent towards the lower *δ*-values than that seen in the higher *δ*-corners. This happens as a result of saturation of the plasma constituents in the SIP towards the maximum *δ*-SIP scenarios with an increase in the *δ*-value, and so forth.

As in Fig. 14b, we find the appearance of a common vertex of the negative ion density trails irrespective of the positive-to-negative ionic mass ratio. This vertex region represents the negative ion density near the *ξ* ≈ 0 regions. The difference in the *m*_*i*_*/m*_*–*_sensitivities of the trail becomes less prominent with an increase in the negative ionic mass as the formation of the heavy negative ions is not favoured in the SIP medium (Fig. 12b). The downward movement along the trail corresponds to the constituents density encountered in moving away from the heliocenter towards the SSB. This off-centric declining trend of the SIP constituents is fairly in accord with the basic physical insights already portrayed in Fig. 12b.

In a similar way, from Fig. 14c, we see the appearance of a common vertex of the negative ion density trail irrespective of the positive ion-to-electron temperature ratio. The vertex corresponds to the negative ion population density near the ξ ≈ 0 regions. The difference in the *T*_*i*_*/T*_*e*_-sensitivities of the trails becomes less prominent with higher *T*_*i*_*/T*_*e*_-values. This behaviour of the trail is attributable to the property discussed in case of studying Fig. 10c. The downward movement along the trails corresponds to the plasma constituent density as encountered by an observer in moving away from the heliocenter towards the SSB (in agreement with Fig. 12c).

We see further, as in Fig. 14d, the appearance of a common vertex of the negative ion density trails irrespective of the negative ion-to-electron temperature ratio, which corresponds to the population density near the *ξ* ≈ 0 regions, as already found in the previous cases as well (Figs. 14a–c). The difference in the *T*_−_*/T*_*e*_-sensitivities of the trails becomes less prominent with higher *T*_−_*/T*_*e*_-values. This behaviour of the trail is attributable to the property discussed in studying Fig. 12d. The downward movement along the trails corresponds to the constituents population density encountered in moving away from the heliocenter towards the SSB (as clearly depicted in Fig. 12d as well). As a consequence, we can draw a common conclusive remark from the above discussion that the heliocentric density of the constituent species is absolutely the same irrespective of the variations in the input constitutive characteristic parameters, such as *δ*, *m*_*i*_*/m*_−_, *T*_*i*_*/T*_*e*_ and *T*_−_*/T*_*e*_ (Fig. 14a–d).

It is to be noted here after observing Figs. 10a and 14a that the positive ion density approaches negligible value towards the heliocenter. So, the medium deviates from the equilibrium plasma quasi-neutrality in the heliocenter. This deviation increases with a decrease in the *δ*-value. It is also noticed from Fig. 10a that as one moves away from the heliocenter towards the SSB, and also with a decrease in the *δ*-value, the positive ion population density takes increasingly negative magnitude. So, it can be inferred, interestingly, that the SIP medium structurizes itself in such a way that its deviation from the equilibrium solar plasma quasi-neutrality increases with an increase in the radial distance as well as a decrease in the *δ*-value.

**Figure 15:**
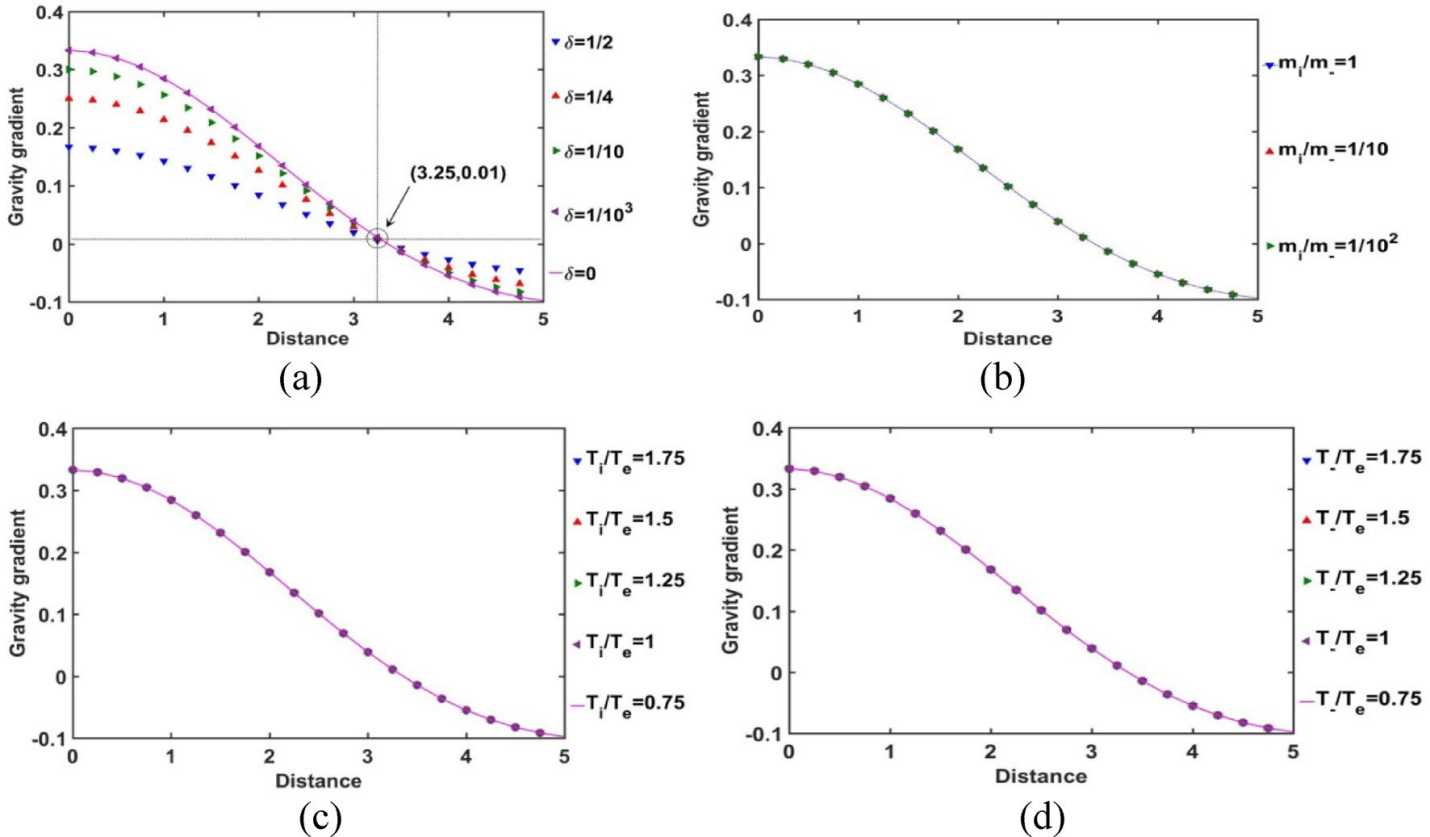
Variation of the gradient of the SIP self-gravitational field strength (gravity) with the Jeans-normalized heliocentric radial distance for different values of the (**a**) equilibrium negative-to-positive ion density ratio (*δ*) with fixed *m*_*i*_*/m*_−_ = 1, *T*_*i*_*/T*_*e*_ = 1 and *T*_−_*/T*_*e*_ = 1; (**b**) positive-to-negative ion mass ratio (*m*_*i*_*/m*_−_) with fixed *δ* = 1/1000, *T*_*i*_*/T*_*e*_ = 1 and *T*_−_*/T*_*e*_ = 1; (**c**) positive ion-to-electron temperature ratio (*T*_*i*_*/T*_*e*_) with fixed *δ* = 1/1000, *m*_*i*_*/m*_−_ = 1 and *T*_−_*/T*_*e*_ = 1; and (**d**) negative ion-to-electron temperature ratio (*T*_−_*/T*_*e*_) with fixed *δ* = 1/1000, *m*_*i*_*/m*_−_ = 1 and *T*_*i*_*/T*_*e*_ = 1.

In Fig. 15, the spatial variation of the normalized SIP self-gravitational field strength gradient is portrayed. It is seen from Fig. 15a that the non-uniformity in the self-gravity of the SIP is sensitive to the *δ*-variation. This self-gravity non-uniformity is higher towards the heliocentric region, which is due to higher inhomogeneity in non-local gravitating material distribution towards the heliocentric region. It is noteworthy that the appearance of a *δ*-insensitive location for the self-gravity non-uniformity (i.e., a trans-critical point) is prominent at *ξ ≈* 3.25. After that location, the self-gravity gradient follows the opposite trend to that from the heliocenter to *ξ ≈* 3.25. We further speculate that the self-gravity gradient is independent of the *m*_*i*_*/m*_−_, *T*_*i*_*/T*_*e*_, and* T*_−_*/T*_*e*_-variation cases of the SIP medium as seen from Fig. 15b–d, respectively. This behaviour signifies that the radial inhomogeneity of the net gravitating matter population distribution in the SIP is insensitive to these three parameters (*m*_*i*_*/m*_−_, *T*_*i*_*/T*_*e*_, and* T*_−_*/T*_*e*_) above against the *δ*-variation scenarios.

**Figure 16:**
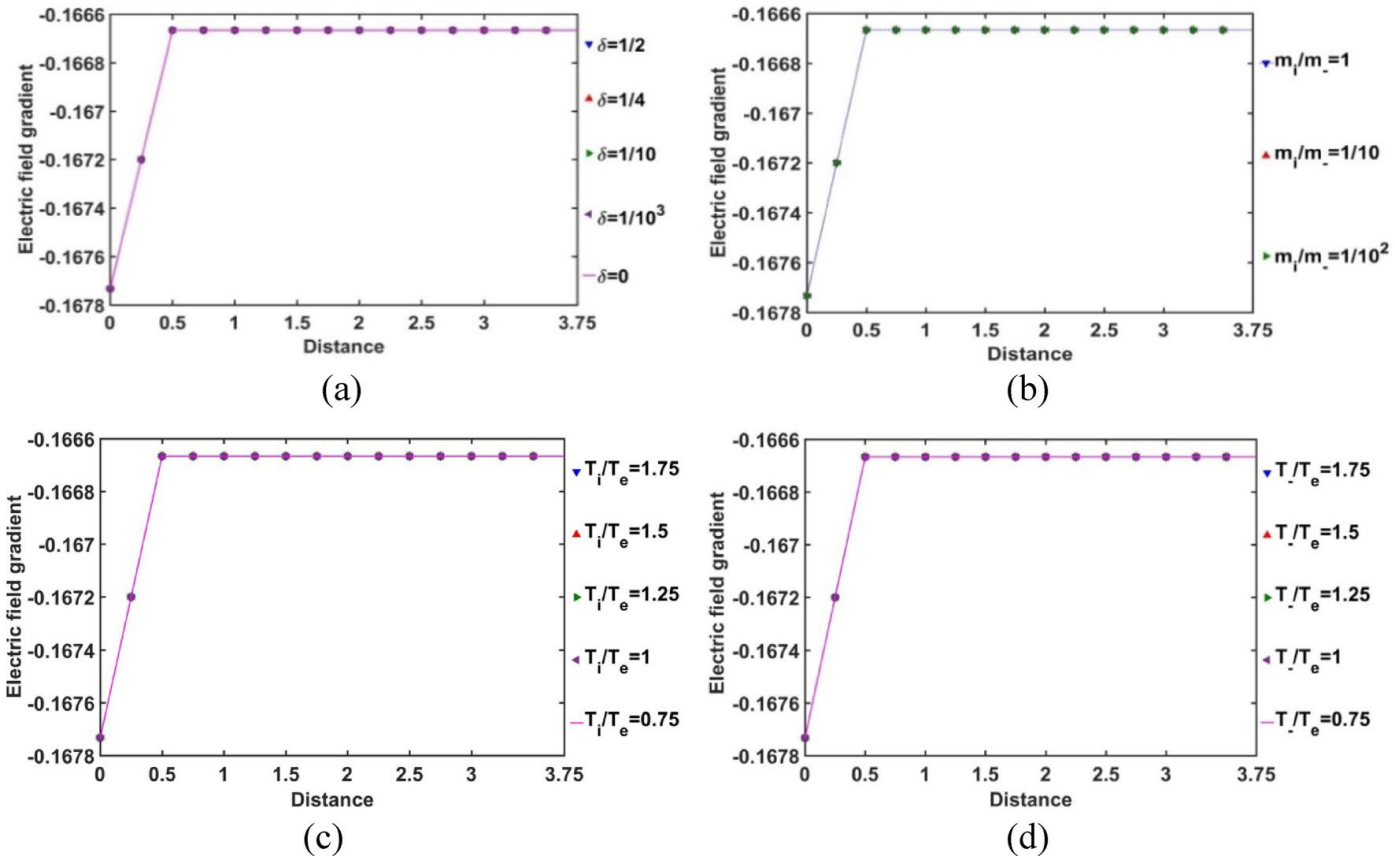
Variation of the gradient of the SIP electric field strength with the Jeans-normalized heliocentric radial distance for different values of the (**a**) equilibrium negative-to-positive ion density ratio (*δ*) with fixed *m*_*i*_*/m*_−_ = 1, *T*_*i*_*/T*_*e*_ = 1 and *T*_−_*/T*_*e*_ = 1; (**b**) positive-to-negative ion mass ratio (*m*_*i*_*/m*_−_) with fixed *δ* = 1/1000, *T*_*i*_*/T*_*e*_ = 1 and *T*_−_*/T*_*e*_ = 1; (**c**) positive ion-to-electron temperature ratio (*T*_*i*_*/T*_*e*_) with fixed *δ* = 1/1000, *m*_*i*_*/m*_−_ = 1 and *T*_−_*/T*_*e*_ = 1; and (**d**) negative ion-to-electron temperature ratio (*T*_−_*/T*_*e*_) with fixed *δ* = 1/1000, *m*_*i*_*/m*_−_ = 1 and *T*_*i*_*/T*_*e*_ = 1.

In Fig. 16, the gradient of the SIP electric field strength is plotted with the Jeans-normalized heliocentric radial distance for different values of *δ* (Fig. 16a), *m*_*i*_*/m*_−_ (Fig. 16b), *T*_*i*_*/T*_*e*_ (Fig. 16c) and *T*_−_*/T*_*e*_ (Fig. 16d). The electric field increment is uniform throughout the SIP scale, but except near the heliocentric region. It is seen further that, from the heliocenter to *ξ ≈* 0.5, a relative decrease in material concentration causes an increase in the electric field gradient and hence, an increase in the electric field strength; and vice-versa.

**Figure 17:**
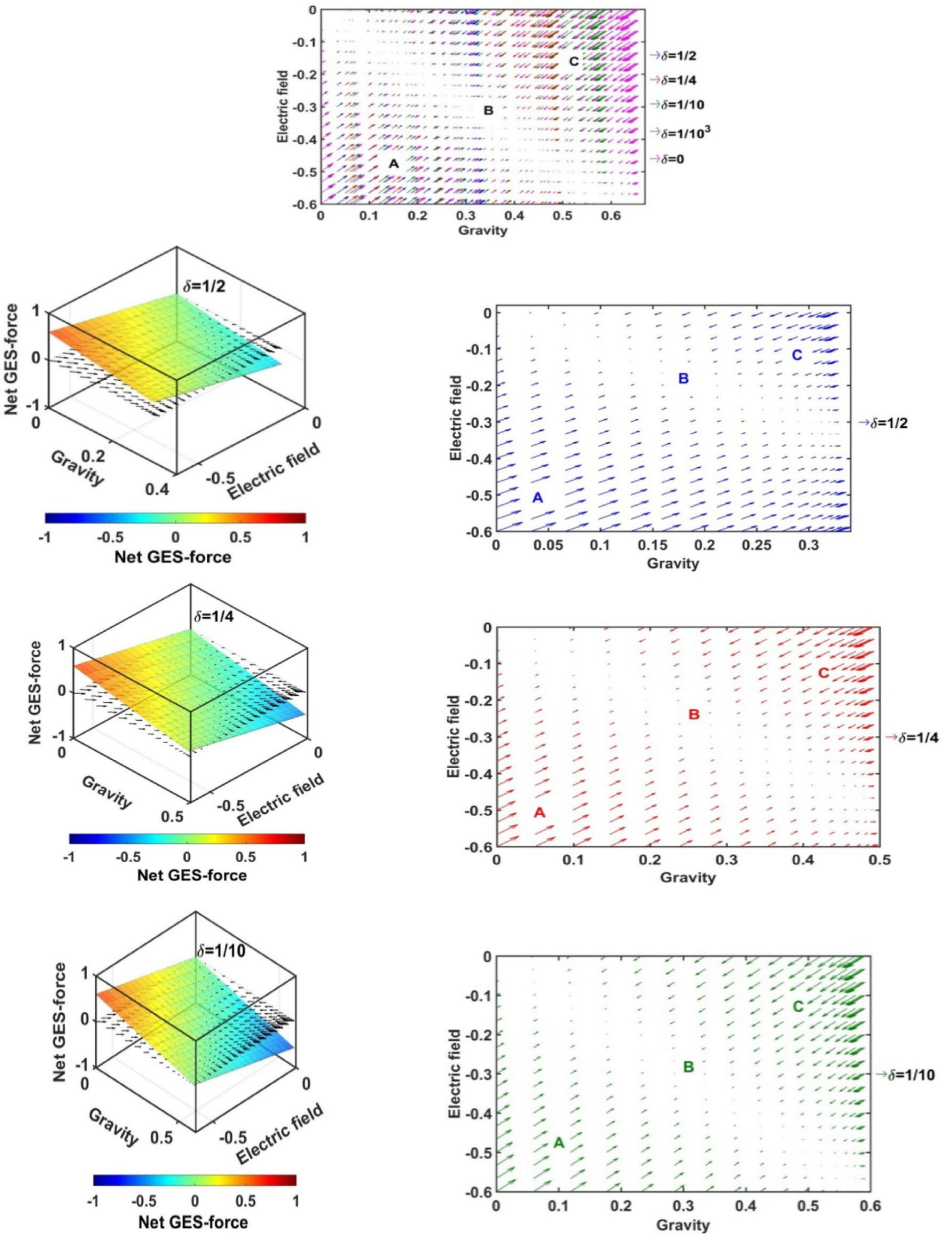

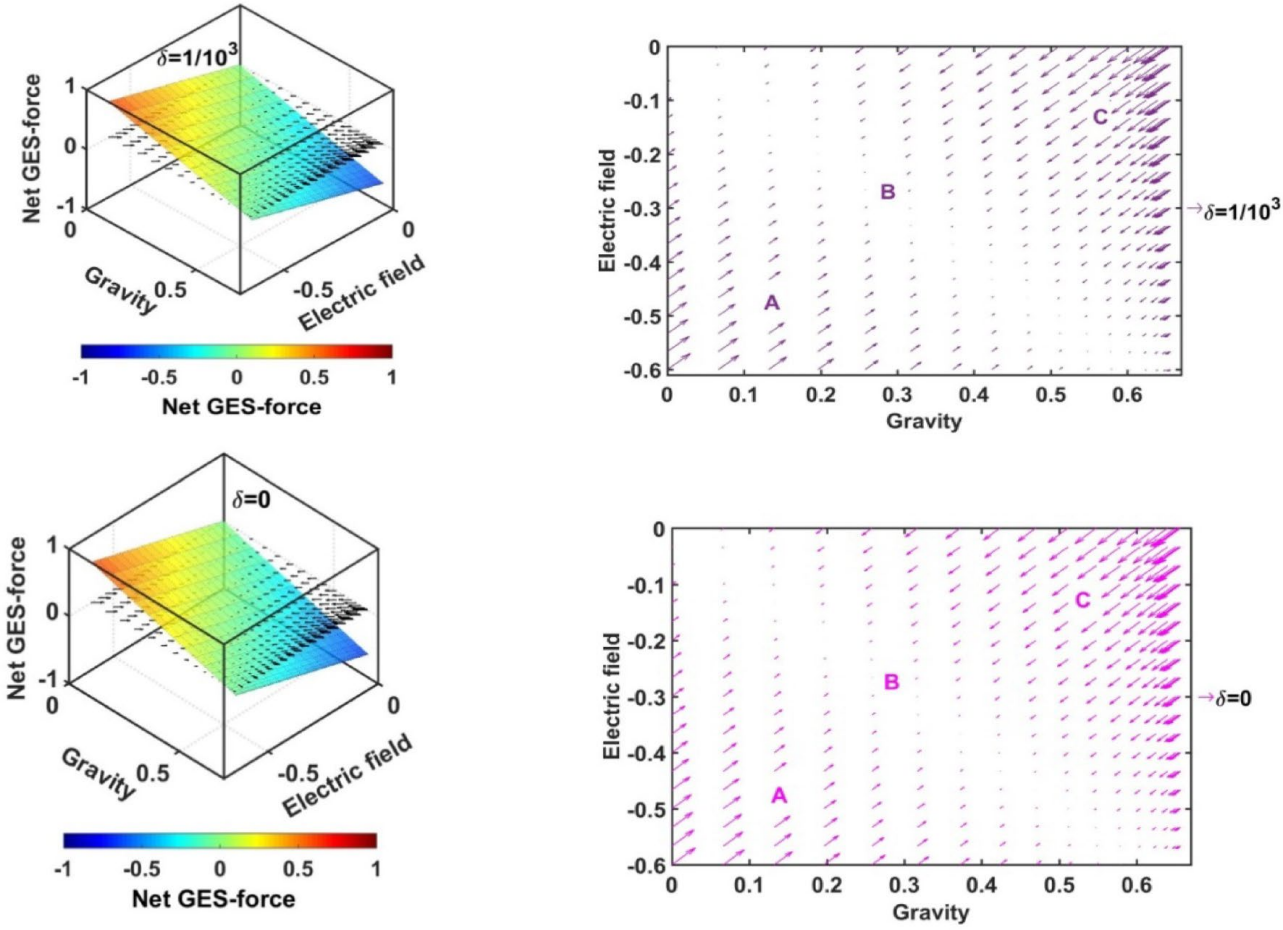
Profile of the net GES-force variation in the SIP with the electric field and self-gravity strength for different values of the equilibrium negative-to-positive ion density ratio (*δ*) with fixed *m*_*i*_*/m*_−_ = 1, *T*_*i*_*/T*_*e*_ = 1 and *T*_−_*/T*_*e*_ = 1.

**Figure 18:**
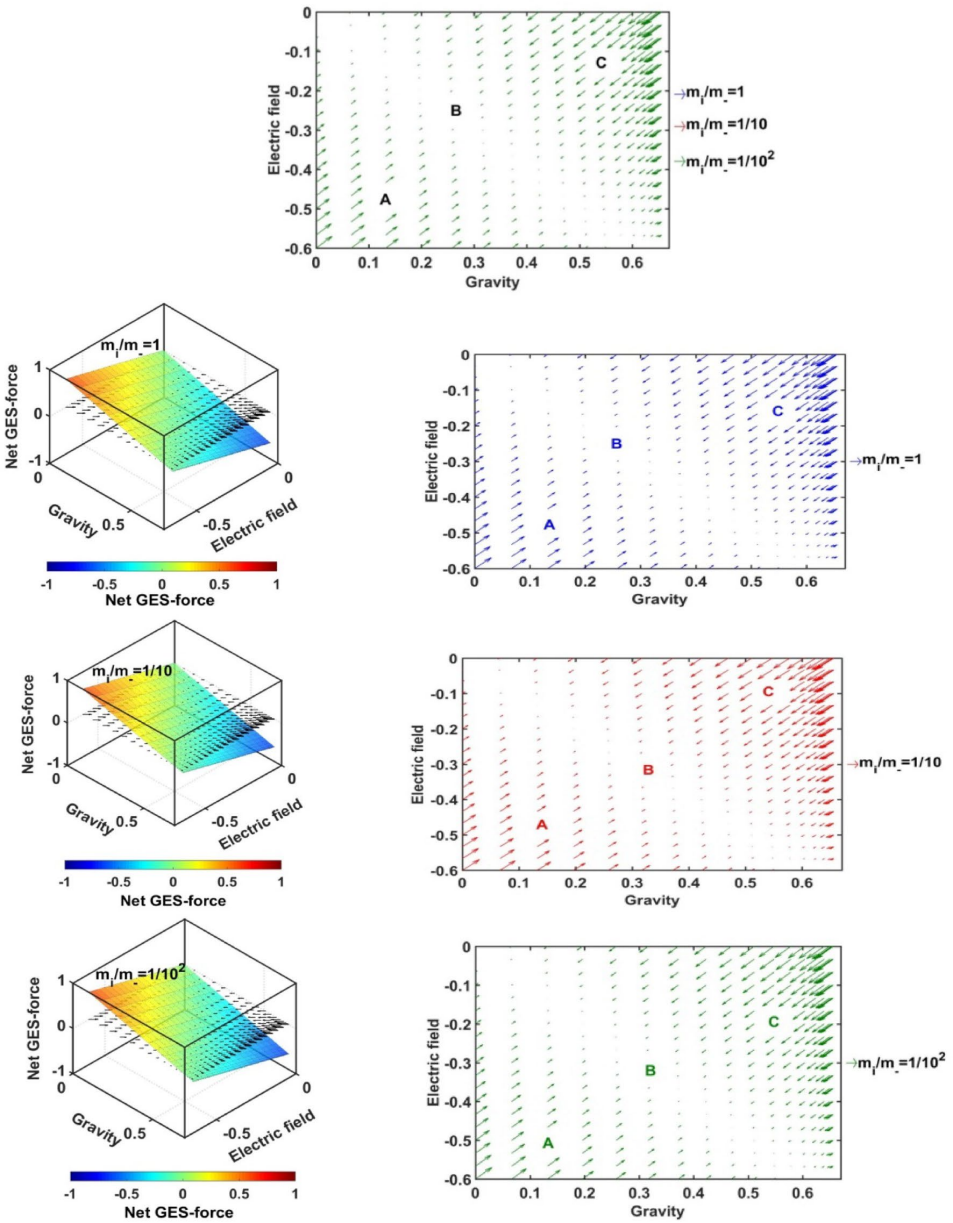
Profile of the net GES-force variation in the SIP with the electric field and self-gravity strength for different values of the positive-to-negative ion mass ratio (*m*_*i*_*/m*_−_) with fixed *δ* = 1/1000, *T*_*i*_*/T*_*e*_ = 1 and *T*_−_*/T*_*e*_ = 1.

**Figure 19:**
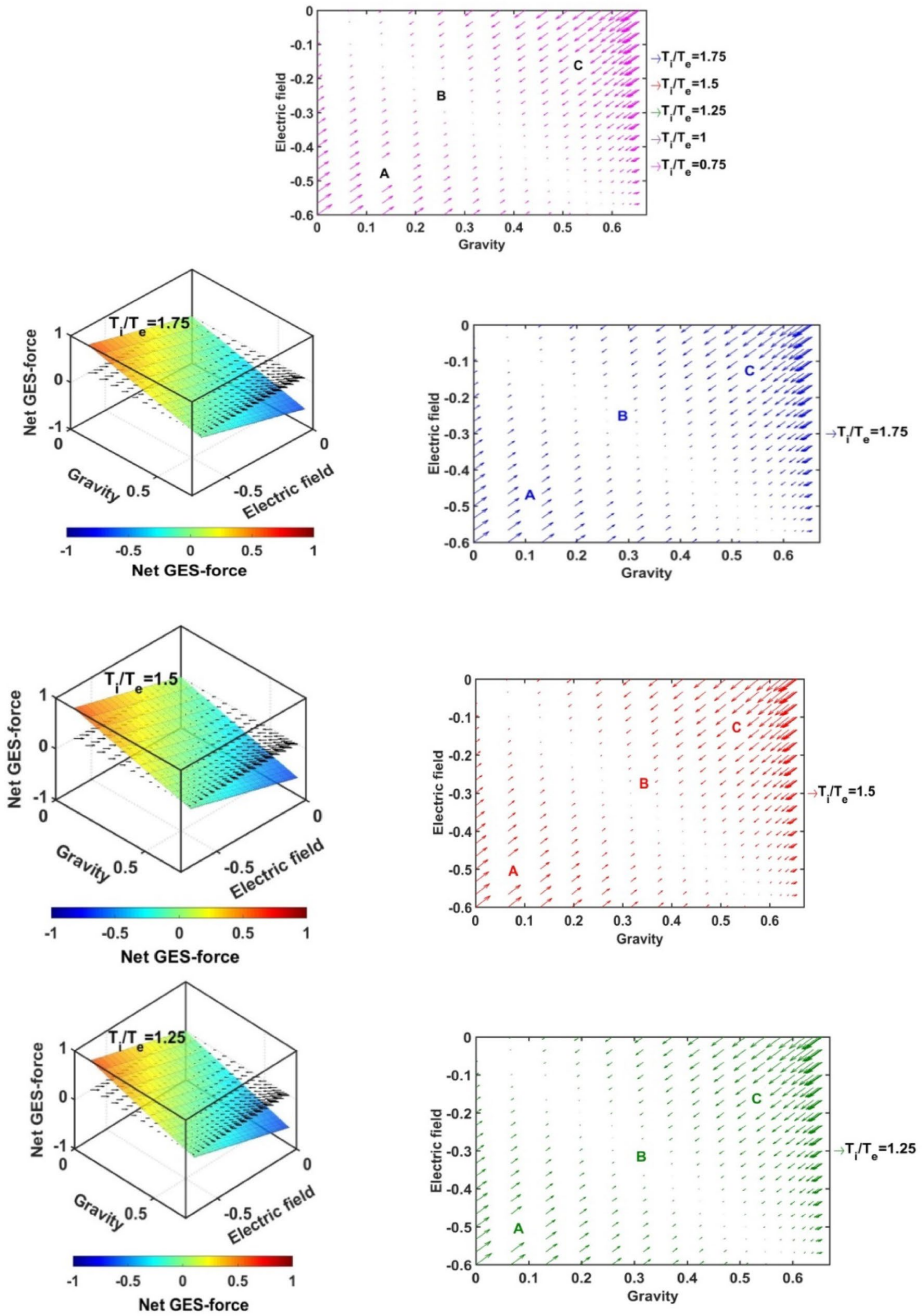

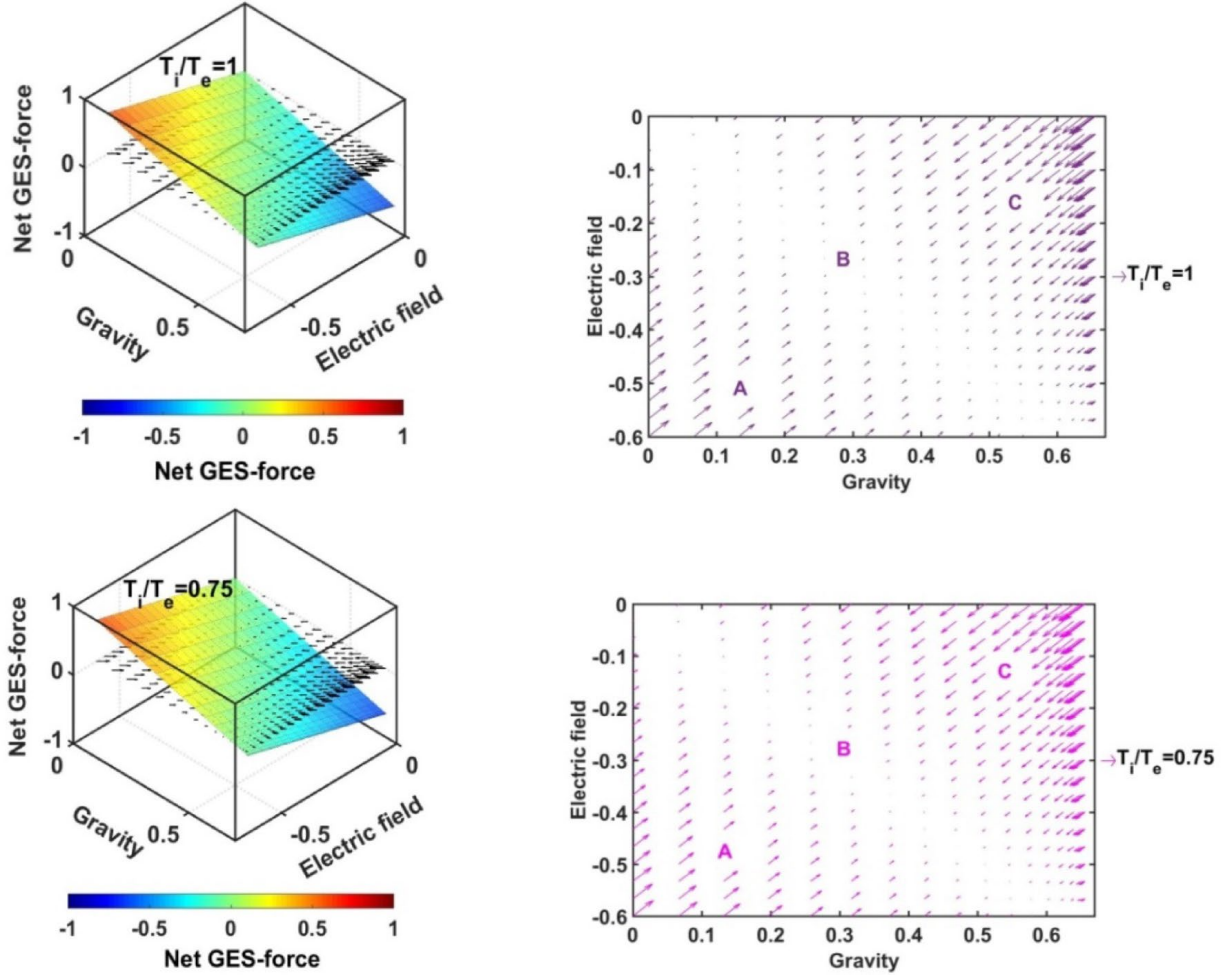
Profile of the net GES-force variation in the SIP with the electric field and self-gravity strength for different values of the positive ion-to-electron temperature ratio (*T*_*i*_*/T*_*e*_) with fixed *δ* = 1/1000, *m*_*i*_*/m*_−_ = 1 and *T*_−_*/T*_*e*_ = 1.

**Figure 20:**
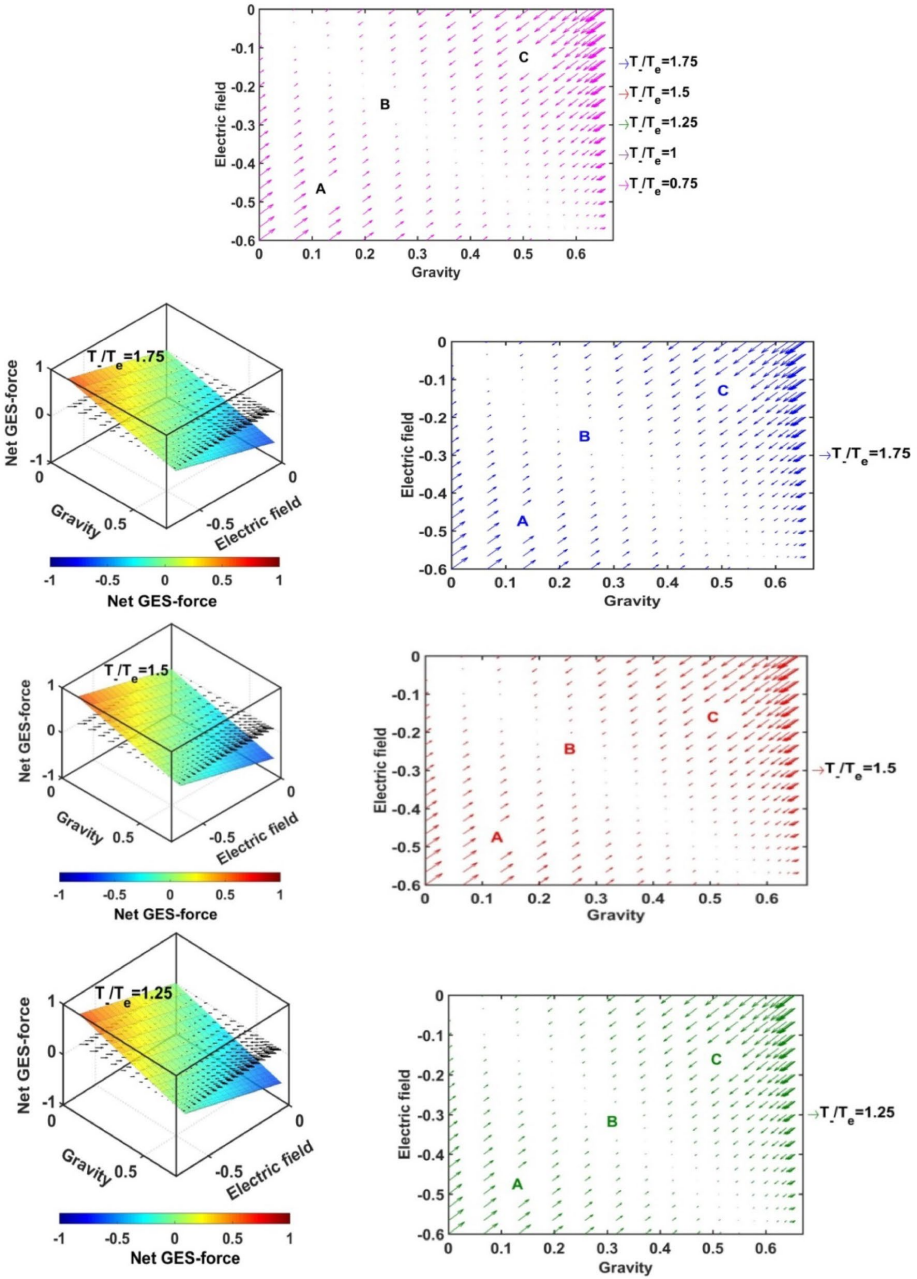

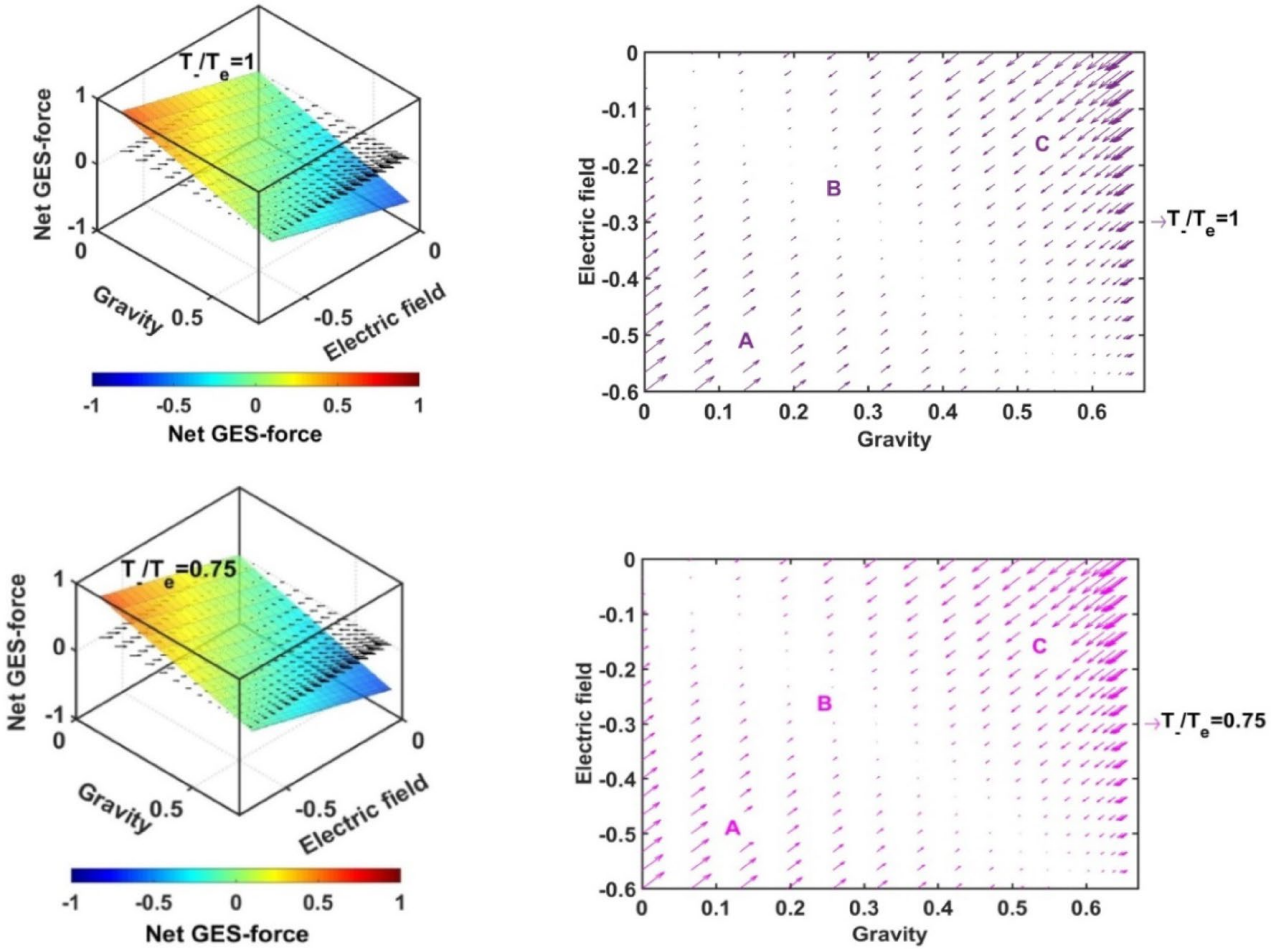
Profile of the net GES-force variation in the SIP with the electric field and self-gravity strength for different values of the negative ion-to-electron temperature ratio (*T*_−_*/T*_*e*_) with fixed *δ* = 1/1000, *m*_*i*_*/m*_−_ = 1 and *T*_*i*_*/T*_*e*_ = 1.

In Figs. 17, 18, 19 and 20, we portray the profile of the net SIP GES-force together with its conjugated gravito-electrostatic components (gravito-electrostatic phase space) for different values of *δ* (Fig. 17), *m*_*i*_*/m*_−_ (Fig. 18), *T*_*i*_*/T*_*e*_ (Fig. 19) and *T*_−_*/T*_*e*_ (Fig. 20). It illustrates the 2-D flow vectors with their length and direction uniquely specifying the net GES-force vectors. The combined pattern of such vectorial flow variations in the form of 3-D graphical structures is also illustrated therein. It is interestingly seen that the dynamical behaviours of the various constituent species composing the entire SIP system with the following characteristic properties can be mapped to the distinct regions of the 2-D vector plots (Figs. 17, 18, 19 and 20) categorized as follows:

- Very light but highly charged particle dynamics (the lower left region);
- Very light neutral particle dynamics (the upper left region);
- Very heavy and highly charged particle dynamics (the lower right region);
- Very heavy neutral particle dynamics (the upper right region).

In a similar way, the dynamical behaviours of the particles with variation in the above-mentioned properties can also be explored by moving along the different directions on the plots as discussed below in the four distinctly classified cases as mentioned above.

Case (a): The very light but highly charged positive particles will face high electric but negligible self-gravitational force in the SIP. Such particles will be driven away from there (i.e. region A) towards the region where it will face low electric and high gravitational force (i.e. towards the region B). The high gravity and low electrostatic effects mean condensation of the single particles to form material lumps. For such material lumps, the net-GES force tends to be zero. So, it can be inferred that the very light but highly charged positive particles will follow a way to the near-SSB region in the SIP and form material lumps to balance gravity and electrostatic effects eventually. It is also noticed that such drifting nature of the SIP material decreases towards the SSB due to increasingly well balancing nature of the self-gravity and electrostatic forces. However, a negative ionic particle will follow the just opposite behaviour. They will travel from the region B (i.e., near-SSB location) to the region A. Such particles will be unable to form material lumps due to low self-gravity environment in the region A. This conclusion again matches with the result already discussed in case of Fig. 12b. It shows that the presence of very massive negative ions (*m*_*i*_*/m*_−_ ~ 10^–2^) is not a favourable factor in the SIP towards the formation of the idealized GES equilibrium structure explained earlier (Fig. 12b).

Case (b): It is further found that very light and neutral particles are almost in a steady state because of the exact gravito-electrostatic force-balancing. Such a situation always appears in the near-heliocentric region. But, with an increase in the positive charge of the lighter particles, they drift more and more towards the near-SSB region and vice-versa (as explored in Case (a)).

Case (c): The very heavy and also highly charged positive particles are almost in a steady-state. Such a situation again appears in the near-SSB region. But with a decrease either in charge or mass, they start drifting more and more towards the region B. This movement of positive ions is opposite to that of negative ions in the SIP.

Case (d): The very heavy but neutral particles fall freely under self-gravity action (region C). Such a situation appears in the near-SSB region. But with either increase in positive charge or decrease in mass, they are less drifted towards the region B and vice-versa.

From Fig. 17, it is seen that the magnitudes of the self-gravitational field along the gravity axis increase with a decrease in the *δ*-value and vice-versa. This behavioural trend is in accord with the SSB formation (with the maximum self-gravity) behaviour with the *δ*-variation (as in Figs. 2a and 3a). The region of gravito-electrostatic equilibrium (i.e. region B) signifies the scenarios of the near-SSB region. This SSB region appears within the diagonal zone, characterizing the zero-value of the net GES-force, when all *δ*-specific plots are combined together simultaneously.

Again, as evident from Fig. 18, it is inferred that the net GES-force pattern is insensitive to the increasing negative ion mass (as previously found in Fig. 3b). It may be due to their very less population compared to the positive ion population. As a result, the overall particle drifting behaviour, and hence SIP-material structurization is independent of the negative ion mass.

From Fig. 19, it is clear again that the net GES-force variation behaviour is insensitive to the variation in the positive ion-to-electron temperature ratio (as previously found in Fig. 3c). So, the degree of inter-particle collision, that is induced by the high positive ionic temperature, does not affect the particle drifting and hence structurization in the SIP.

It is seen in Fig. 20 that the net GES-force variation with its electric and gravitational field is insensitive to the negative ion-to-electron temperature ratio (as previously found in Fig. 3d). So, the degree of inter-particle collision, which is induced by the high negative ionic temperature, does not affect the particle drifting and hence structurization in the SIP.

### SWP-illustration

**Figure 21:**
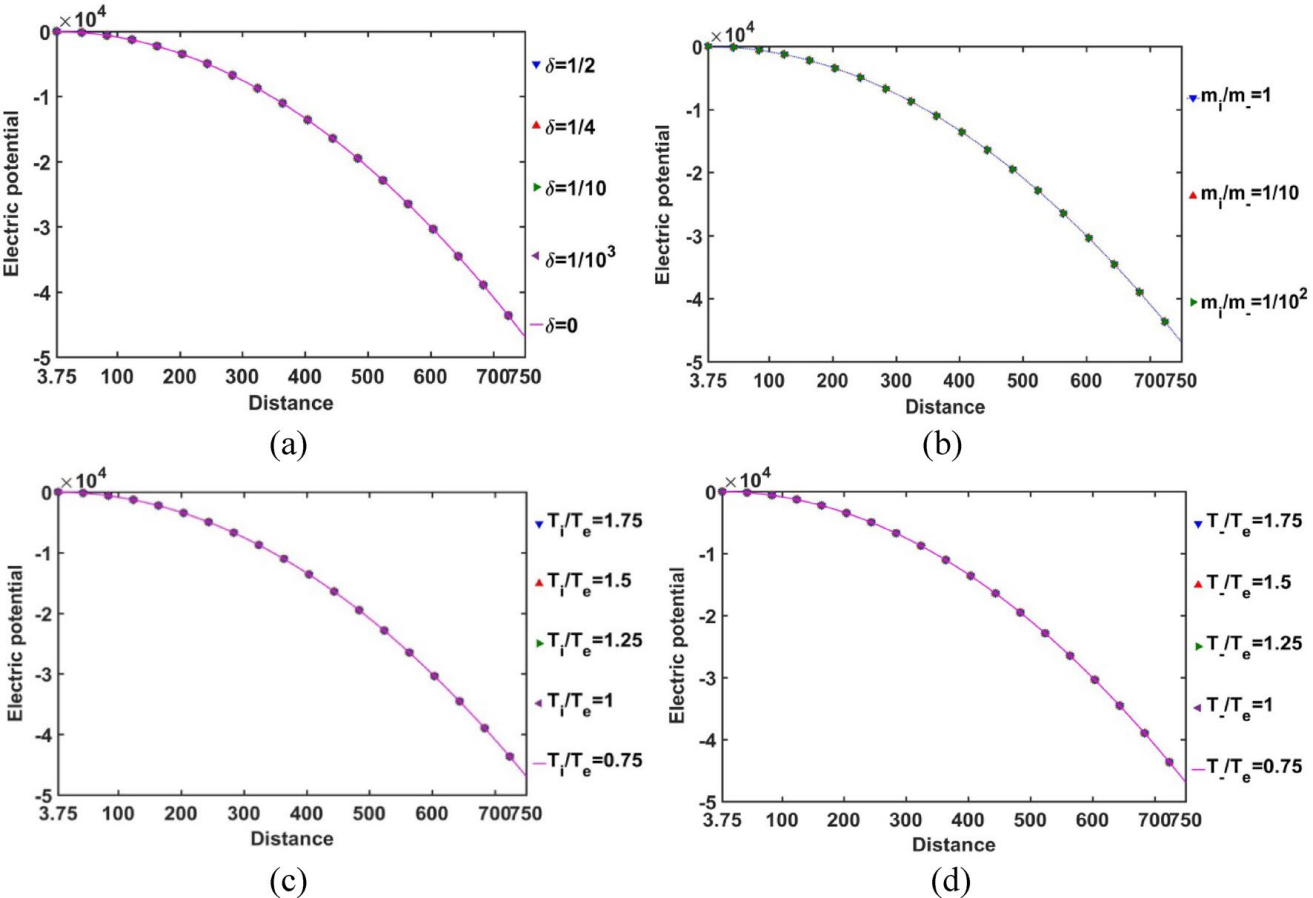
Variation of the normalized SWP electric potential with the Jeans-normalized heliocentric radial distance for different values of the (**a**) equilibrium negative-to-positive ion density ratio (*δ*) with fixed *m*_*i*_*/m*_−_ = 1, *T*_*i*_*/T*_*e*_ = 1.25 and *T*_−_*/T*_*e*_ = 1.25; (**b**) positive-to-negative ion mass ratio (*m*_*i*_*/m*_−_) with fixed *δ* = 1/1000, *T*_*i*_*/T*_*e*_ = 1.25 and *T*_−_*/T*_*e*_ = 1.25; (**c**) positive ion-to-electron temperature ratio (*T*_*i*_*/T*_*e*_) with fixed *δ* = 1/1000, *m*_*i*_*/m*_−_ = 1 and *T*_−_*/T*_*e*_ = 1.25; and (**d**) negative ion-to-electron temperature ratio (*T*_−_*/T*_*e*_) with fixed *δ* = 1/1000, *m*_*i*_*/m*_−_ = 1 and *T*_*i*_*/T*_*e*_ = 1.25 as per the recent solar observational reports.

**Figure 22:**
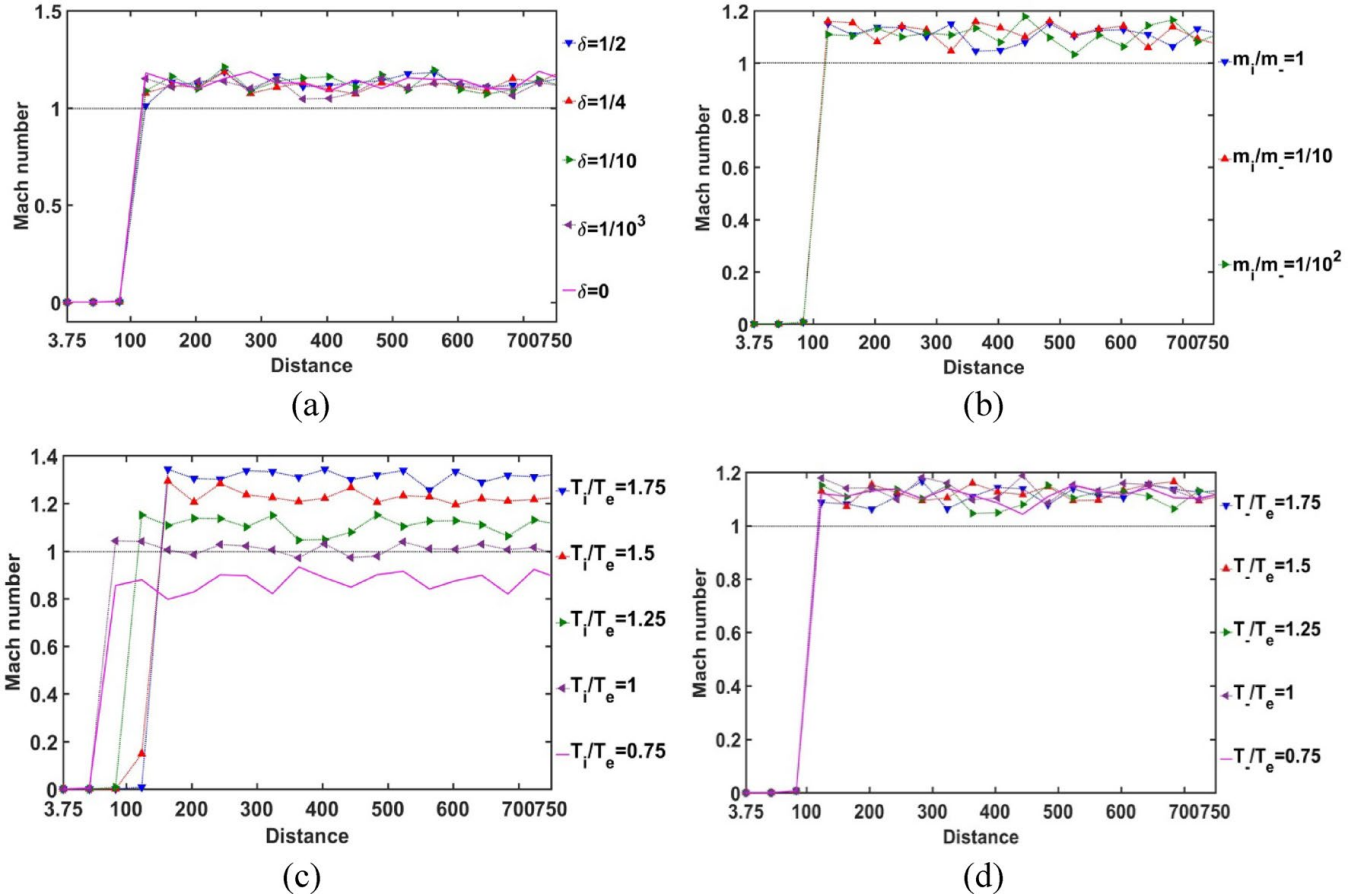
Variation of the SWP Mach number with the Jeans-normalized heliocentric radial distance for different values of the (**a**) equilibrium negative-to-positive ion density ratio (*δ*) with fixed *m*_*i*_*/m*_−_ = 1, *T*_*i*_*/T*_*e*_ = 1.25 and *T*_−_*/T*_*e*_ = 1.25; (**b**) positive-to-negative ion mass ratio (*m*_*i*_*/m*_−_) with fixed *δ* = 1/1000, *T*_*i*_*/T*_*e*_ = 1.25 and *T*_−_*/T*_*e*_ = 1.25; (**c**) positive ion-to-electron temperature ratio (*T*_*i*_*/T*_*e*_) with fixed *δ* = 1/1000, *m*_*i*_*/m*_−_ = 1 and *T*_−_*/T*_*e*_ = 1.25; and (**d**) negative ion-to-electron temperature ratio (*T*_−_*/T*_*e*_) with fixed *δ* = 1/1000, *m*_*i*_*/m*_−_ = 1 and *T*_*i*_*/T*_*e*_ = 1.25.

To explore the equilibrium SWP behaviours, its various relevant properties are studied numerically with the help of the normalized SWP governing equations (Eqs. 30–38). Here, the radial grid size used is 40. For plotting the SWP profiles, we have considered *T*_*i(-)*_*/T*_*e*_ = 1.25. The reason behind is that the SWP Mach number turns supersonic for *T*_*i*_*/T*_*e*_ > 1 (Fig. 22). So, keeping in mind the observed supersonic nature of the solar wind particles, the rest of the profiles here are structured for *T*_*i*_*/T*_*e*_ = 1.25, which is the considered smallest value in our study, for which the supersonic Mach number is seen. This assumption of *T*_*i(-)*_*/T*_*e*_ > 1 is quite fair in line with diverse solar observational reports, as clearly depicted in S No 5 in Appendix E. In Fig. 21, the radial variation of the normalized SWP electric potential from the SSB to 1 au for *δ*, *m*_*i*_*/m*_−_, *T*_*i*_*/T*_*e*_ and *T*_−_*/T*_*e*_-variations is shown. It is found that the SWP electric potential is insensitive to any of the above-mentioned parametric variations. The SWP electric potential is insignificant in the near-SSB region as compared to the far-SSB region in the SWP medium. It signifies high material concentration in the near SSB-zone. It results in a high degree of shielding effect between the opposite-polarity plasma species. Away from the SSB, the material concentration decreases and due to increase in diffusivity of the SWP constituents, the electrostatic effects become more significant. This result on the constitutive electrostatic response characteristics is fairly in accord with the recently reported thermo-statistically modified solar-picture based on the realistic GES model formalism^11^.

As portrayed in Fig. 22, the spatial variation of the SWP positive ion Mach number from the SSB up to 1 au is depicted for different values of *δ* (Fig. 22a), *m*_*i*_*/m*_−_ (Fig. 22b), *T*_*i*_*/T*_*e*_ (Fig. 22c) and *T*_−_*/T*_*e*_ (Fig. 22d). It is interestingly found that there is an abrupt subsonic-to-supersonic transition of the positive ion Mach number for *T*_*i*_*/T*_*e*_ ≥ 1 just outside the SSB (Fig. 22c). The low density of the SWP medium facilitates the high positive ionic velocity in contrast to the dense SIP medium case as already seen in Fig. 5. This is fairly in agreement with the basic principle of the well-known Newtonian acoustics, as seen extensively in the earlier GES-scenarios as well. It is because of the fact that the bulk plasma flow occurs at the phase speed of the bulk (ion) acoustic mode, and so forth^5^.

The Mach number is highly sensitive to the positive ion-to-electron temperature ratio (*T*_*i*_*/T*_*e*_), as clearly evident in Fig. 22c. It is interestingly noticed that the sonic transition location on the heliocentric radial space shifts away from the SSB outwards with an increase in the *T*_*i*_*/T*_*e*_-value and vice-versa. The Mach number is of the order of unity for *T*_*i*_*/T*_*e*_ = 1. In other words, the positive ion velocity reaches the order of magnitude of the average sound speed in the SIP. With an increase in *T*_*i*_*/T*_*e*_-value, the Mach number increases and vice-versa. So, high positive ionic temperature helps the ions to attain a high velocity in the rare SWP medium.

It is noteworthy here that the magnitude of the solar wind speed, as already obtained from various solar observational missions, is supersonic in nature^16^. So, for such a high-speed scenario, a non-isothermal plasma medium is clearly suggested by our presented model formalism, in accordance with the realistic picture^11,26^. The consistency of this SWP Mach number behaviour with previously reported observations further strengthens the reliability of our proposed calculation scheme. We find throughout that the SWP Mach number at a distance of 1 au comes out approximately to be *M*_1au_ = 1.13 (Fig. 22). This supersonic SWP flow dynamics is quite in accordance with previously reported results^5^.

**Figure 23:**
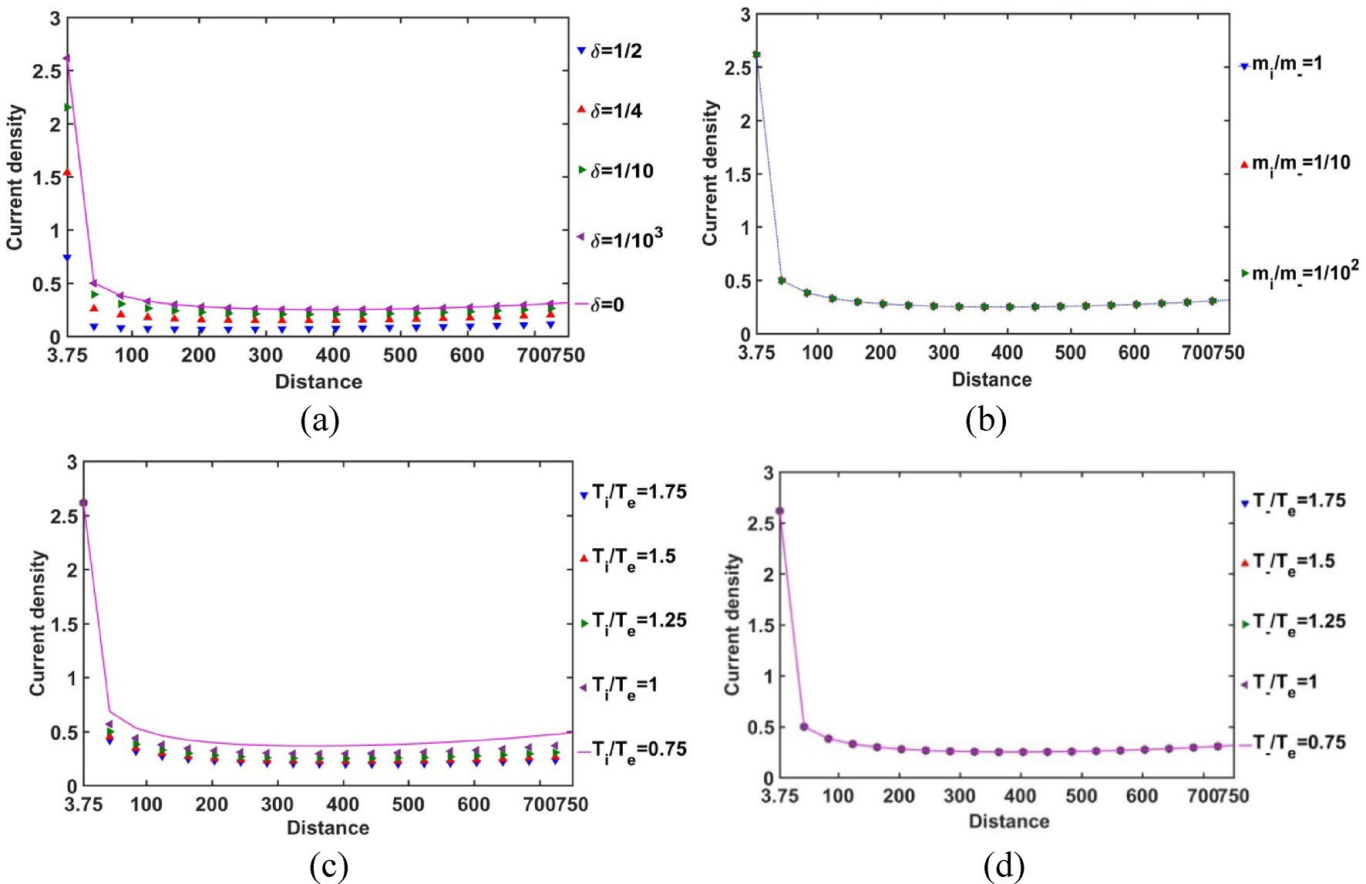
Variation of the SWP electric current density with the Jeans-normalized heliocentric radial distance for different values of the (**a**) equilibrium negative-to-positive ion density ratio (*δ*) with fixed *m*_*i*_*/m*_−_ = 1, *T*_*i*_*/T*_*e*_ = 1.25 and *T*_−_*/T*_*e*_ = 1.25; (**b**) positive-to-negative ion mass ratio (*m*_*i*_*/m*_−_) with fixed *δ* = 1/1000, *T*_*i*_*/T*_*e*_ = 1.25 and *T*_−_*/T*_*e*_ = 1.25; (**c**) positive ion-to-electron temperature ratio (*T*_*i*_*/T*_*e*_) with fixed *δ* = 1/1000, *m*_*i*_*/m*_−_ = 1 and *T*_−_*/T*_*e*_ = 1.25; and (**d**) negative ion-to-electron temperature ratio (*T*_−_*/T*_*e*_) with fixed *δ* = 1/1000, *m*_*i*_*/m*_−_ = 1 and *T*_*i*_*/T*_*e*_ = 1.25.

As shown in Fig. 23, we speculate the spatial variation of the Bohm-normalized SWP electric current density portrayed for different values of *δ*, *m*_*i*_*/m*_−_, *T*_*i*_*/T*_*e*_ and *T*_−_*/T*_*e*_. It is seen that the current density decreases with an increase in *δ* and vice-versa (Fig. 23a), like the corresponding SIP case (Fig. 6a). The *m*_*i*_*/m*_*–*_variation does not affect the net SWP current density (Fig. 23b), as in the SIP (Fig. 6b). It is seen from Fig. 23c that the SWP current density is dependent on the *T*_*i*_*/T*_*e*_-value in the same manner, and hence governed by the same physical principles as the SIP current density (Fig. 6c). However, the *T*_−_*/T*_*e*_-variations do not influence the net SWP electric current density, as seen from Fig. 23d, like the SIP (Fig. 6d).

**Figure 24:**
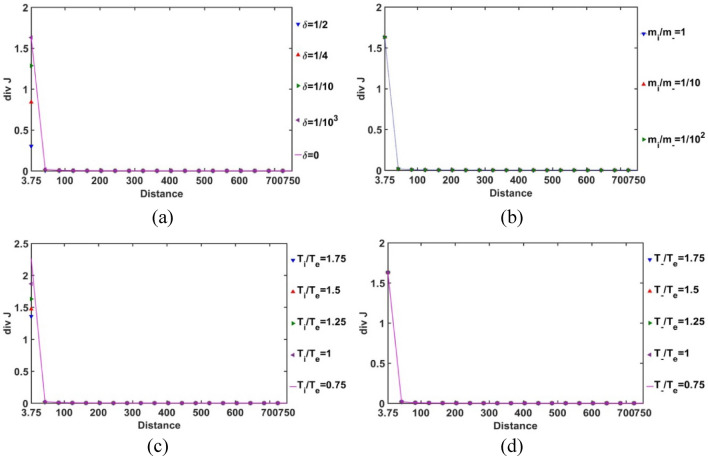
Variation of the divergence of the SWP electric current density (div *J*) with the Jeans-normalized heliocentric radial distance for different values of the (**a**) equilibrium negative-to-positive ion density ratio (*δ*) with fixed *m*_*i*_*/m*_−_ = 1, *T*_*i*_*/T*_*e*_ = 1.25 and *T*_−_*/T*_*e*_ = 1.25; (**b**) positive-to-negative ion mass ratio (*m*_*i*_*/m*_−_) with fixed *δ* = 1/1000, *T*_*i*_*/T*_*e*_ = 1.25 and *T*_−_*/T*_*e*_ = 1.25; (**c**) positive ion-to-electron temperature ratio (*T*_*i*_*/T*_*e*_) with fixed *δ* = 1/1000, *m*_*i*_*/m*_−_ = 1 and *T*_−_*/T*_*e*_ = 1.25; and (**d**) negative ion-to-electron temperature ratio (*T*_−_*/T*_*e*_) with fixed *δ* = 1/1000, *m*_*i*_*/m*_−_ = 1 and *T*_*i*_*/T*_*e*_ = 1.25.

In Fig. 24, we depict the radial variation of the divergence of the Bohm-normalized SWP electric current density for different values of *δ*, *m*_*i*_*/m*_−_, *T*_*i*_*/T*_*e*_ and *T*_−_*/T*_*e*_. It is found that the current density is fairly conserved throughout the entire SWP medium, except in the near-SSB regions. There appears no source or sink, as in the SIP case as well, to affect the net charge production and its directional flow in the SWP, except in the near-SSB regions (*ξ* ≈ 3.75–30). The finite non-zero positive divergence of the net electric current density in the near-SSB regions is attributable to the high charge density of these regions, unlike the diffuse far-SSB regions (*ξ* > 30). The conservative nature of the electric current density is independent of any parametric variations, such as *δ* (Fig. 24a), *m*_*i*_*/m*_−_ (Fig. 24b), *T*_*i*_*/T*_*e*_ (Fig. 24c) and *T*_−_*/T*_*e*_ (Fig. 24d) as explored.

## Conclusions

The presented theoretical exploration reveals various equilibrium solar properties founded on the modified plasma-wall interaction-based gravito-electrostatic sheath (GES) model formalism refined methodically with the help of a proper inclusion of the realistic negative ionic effects for the first time. The considered spherical solar plasma volume consists of the Boltzmann-distributed inertialess electrons, gravito-electrostatically coupled with the positive–negative ionic inertial fluids, via the Poisson formalism, on the relevant astrophysical scales. The zeroth-order equilibrium configuration of the bounded solar plasma system is considered to be quasi-neutral hydrostatic homogeneous in nature. The relevant basic governing equations for the tri-component plasmas are systematically developed for describing both the SIP- and SWP- scaled dynamics moderated by the long-range non-local self-gravity and external gravity, respectively.

It is noteworthy in the context of applicability of the fluid treatment here in our study that the mean density of the SWP near the coronal region is ~ 10^14^ m^-3^. The modelling of such a coronal plasma configuration is justifiably consistent with an isothermal hydrostatic equilibrium configuration^49^. To be more precise with mathematical rigor, we consider the expression of the collisional mean free path (*λ*) between constitutive particles as: *λ* = 1*/*(*nσ*), where *n* and *σ* stands respectively for the number density of colliding particles and the corresponding collisional cross-section^50^. In the microscopic scale, one finds *σ* ~ 10^–19^ m^2^. Now, with *n* ~ 10^14^ m^−3^, one gets *λ* ~ 10^5^ m. This mean free path is much smaller than the critical Jeans scale length (S. No. 23 in Appendix A). This Jeans length is taken as the unit of the measuring spatial scale of both the SIP (*r* = 3.5 *λ*_*J*_) and SWP (*r* = 750 *λ*_*J*_). Hence, the applied fluid treatment is well justified because of the smallness of the mean free path in comparison with all the characteristic plasma scale lengths^50^. In addition, it is well known that the kinetic theory gives a microscopic individualistic picture of the ongoing physical phenomena; whereas, the fluid theory offers a macroscopic mean pictorial counterpart. Our proposed work is primarily motivated with the latter instead of the former for the sake of analytic simplicity in formulating the composite GES-model structure.

An exact numerical analysis of the equilibrium GES-model (Fig. 1) governing equations reveals an interesting property of the bounded solar plasma volume showing its shrinking nature with an increase in the negative ion concentration in the constitutive SIP medium for the first time. However, this GES-shrinking behaviour is not affected by the mass of the negative ions and temperature of the plasma constituents (Figs. 2 and 3). Such SIP features can be well explicated by the shielding nature of the opposite polarity plasma constituents.

It is to be noted in the above context that, since the plasma sheath is a separate non-neutral region from the primarily quasi-neutral SIP; hence, the SIP shrinking mechanism is distinct from that of the sheath-broadening, as usually encountered on the laboratory scales, with the inclusion of more negative ionic species^51,52^. As the sheath structure evolves in equal horizons on both the bounded SIP (gravitational) and unbounded SWP (electrostatic) scales, any dimensional change in the sheath width does not affect the SIP structure significantly. As a consequence, it is noteworthy that our presented semi-analytical study showing the SIP-shrinking with an increase in the negative ionic concentration on the astronomical scales, against that already found on the laboratory scales, is a unique result reported for the first time.

The spatial variation of the electric potential is found to be insensitive to the negative ion concentration, their mass and plasma constituent temperature in both the SIP and SWP media (Figs. 4 and 21). The solar plasma flow dynamics is analysed with the Mach number and current density profiles for various relevant physical parametric variations (Figs. 5, 6 and 7 and Figs. 22, 23 and 24). In the SWP, the sonic transition of the Mach number is distinctly ruled by the positive ion-to-electron temperature ratio. Hence, it is concluded that for the observed supersonic solar wind particles, the plasma medium must move away from thermal equilibrium. This prediction on the temperature matches with the observation of non-thermal plasma species, as reported in the literature^11^. The current density is sensitive to the negative ion density as well as the positive ion-to-electron temperature ratio in both the SIP and SWP. The self-structurization of the SIP constituents is explored with their radial density variation along with their spatial gradient behaviours (Figs. 8, 9, 10, 11, 12, 13 and 14). The inhomogeneity in the SIP mass and the net electric charge distribution is studied with the radial gradient variation of the self-gravity and electric field strengths (Figs. 15 and 16). Interestingly, a location with *δ*-insensitive gravity gradient is revealed in the SIP. The flow behaviors of the constitutive plasma elements are thoroughly investigated in a defined gravito-electrostatic interaction phase space. It, indeed, clearly portrays the solar material clumping nature in the SIP. This atypical clumping behaviour is reported here for the first time (Figs. 17, 18, 19 and 20). It has been revealed herewith that the SIP does not favor the formation of heavy negative ions. This result well matches with the observation that the hydrogen ion (H^-^) accounts for the largest part of the continuous absorption of the solar atmosphere^14,15^. Thus, our analysis is well strengthened with the astronomical results originating from different solar missions.

It may be noteworthy here that the presented GES-based study throws light on various equilibrium properties of the negative ion-modified GES model-based Sun, sun-like stars, and their surrounding atmospheres. We admit here that, in our proposed model formulation, the realistic magnetic field-induced effects, viscosity, and effective rotational effects are ignored for analytic simplicities^5,18^. The complications, originating from plasma fluid turbulence and thermo-statistical distribution laws of the constitutive non-thermal species^2,11,27^, are also ignored. The temperature anisotropy, originated in the presence of magnetic field^28–30^, is also not taken into account. The basic model formalism ignores the non-radial flow effects in the SIP caused by the complex solar interior magnetic field structures^31^, solar wind atomic particle acceleration caused by the radiation pressure at the cost of Doppler effect^32^, and so forth. In addition to the above, the equation of state here neglects the effects of relativistic electron dynamics, population of excited plasma constituent species, degeneracy pressure, and so forth. These are however verified by varied observational findings and theoretical predictions^33^. Consequently, a proper inclusion of the above-mentioned solar plasma characteristics should open a new scope of refined investigations founded on the current GES-based model scenarios.

It is pertinent to add here that the recent data acquired by the Heavy Ion Sensor (HIS) onboard the Solar Orbiter has confirmed the presence of various heavy elemental species, ranging from He to Fe, with their respective broad range of possible charged states. Such measurements have well supported the investigations of local physical processes occurring in the solar atmosphere. It is also well known that such heavy ionic species can be utilized as tracers of the solar wind origin and their acceleration mechanism within the corona^34^. In the present investigation, we omit such diverse positive ionic species for the sake of analytic simplicity. As a consequence, analysis of the effects of such positive ionic heavy elemental species in the solar plasma flow dynamics in the GES model fabric will hopefully open a new window for the future research in the solar and like stellar plasma systems in more realistic physical configurations. It is noteworthy in the present context that, as the convection-driven solar-like oscillations have recently been reported to exist in the cool K-dwarf stars^53^, the scope of the solar GES model could also be extended to see the asteroseismology of the K-type dwarfs and similar stellar remnant structures about a well-defined GES-equilibrium.

The present model regards the bi-scaled solar plasma medium to be composed of the Boltzmann electrons and fluidic ions (positive and negative). The negative ion population effects in different ratios (in experimentally judicious ranges) compared to the positive ions are studied here for the first time^12,13^. Various explored properties here are well compared with the previously reported GES-based results as well as astronomical observations reported in the literature for a reliability assessment. So, this GES-based study stands well in the contemporary solar astronomic context.

It is noteworthy in the current solar plasma context, that the presented GES model is remarkably successful in explaining the equilibrium solar plasma properties. It focuses mainly on the subsonic origin of solar wind plasma, its subsequent supersonic flow dynamics, and associated relevant characteristic physical parameters even without any vivid magneto-activity here. However, it is now well reported from diverse observations that the fast solar wind originates from a temporarily appearing rarer and cooler regions of the coronal plasma medium, known as the coronal holes, in the solar terminology^35^. Here, the solar magnetic field becomes open and extends into the interplanetary space. But, as in the literature ^35,36^, the origin of the slow component of the solar wind is still an open challenge^37^, although there are evidences for its development in closed field regions or at the boundaries between the open and closed fields.

It has been reported recently in the literature that the jets or jetlets driven by the interchange magnetic reconnection near the coronal base region could be the source of particle heating and hence, acceleration of the solar wind particles to supersonic speed^35,38^. The future solar observations, yet to be performed by the Parker Solar Probe (PSP) along with the Solar Orbiter (SolO) missions^1,28^, are expected to shed more light on the link between magneto-activities and solar wind driving mechanisms. Therefore, such onsite experiments could hopefully pose another venture in the reliability and validation of our proposed investigation in the real solar astronomic scenarios with the judicious incorporation of active negative constitutive species and other highlighted realistic factors.

Apart from all the above, it is noteworthy, in the context of the present solar astronomic scenarios, that a proper utilization of machine learning carries enough capability for amplifying our comprehension of the complex plasma processes occurring in the Sun and its atmosphere. With the help of techniques, such as deep learning, it now seems to be possible to scrutinize extensive quanta of data from the solar observations. It should enable us to see previously unknown patterns and processes that might have caused an illusion in the detection processes through conventional approaches. This technological advancement hopefully holds the potential to illuminate our physical insights into various important dynamical events, like solar flares, CME-driven instabilities, etc., that cause substantial impacts on the Earth and its atmosphere^39^.

### Supplementary Information

Supplementary Information 1.

Supplementary Information 2.

Supplementary Information 3.

Supplementary Information 4.

Supplementary Information 5.

Supplementary Information 6.

## Data Availability

All data generated or analyzed during this study are included in this published article.
