# Supplementary material for: Effects of negative ions on equilibrium solar plasmas in the fabric of gravito-electrostatic sheath model
Source: Sci Rep. 2024 Jul 12;14:16087. doi: 10.1038/s41598-024-66774-8 (PMC11245523; doi:10.1038/s41598-024-66774-8)
Supplement: Supplementary file 1 — Supplementary Information 1. [file 41598_2024_66774_MOESM1_ESM.doc]

**APPENDIX A: SYMBOL AND SIGNIFICANCE**

| **S No** | **Symbol** | **Mathematical significance** | **Physical significance** |
| --- | --- | --- | --- |
| 1 | *r* | Radial distance (heliocentric) | Distance of an observation point from the reference heliocenter in spherical polar coordinates |
| 2 | *t* | Time coordinate | Time between two events as measured by a static observer with respect to heliocenter |
| 3 | *n_e_* | Electron number density | Number of electrons per unit volume in the plasma system |
| 4 | *n_e0_* | Equilibrium electron number density | Equilibrium number of electrons per unit quasi-neutral plasma volume |
| 5 | *T_e_* | Electron temperature | Electronic thermal (kinetic) potential causing directive heat flow |
| 6 | *m_e_* | Electron mass | Measure of electronic resistance to change of its state due to a force field (*m_e_* =9.10 × 10^-31^ kg) |
| 7 | *e* | Electronic (protonic) charge unit | Property of an electron (proton) that causes it to experience a force when placed in an electromagnetic field (1.60 × 10^-19^ C) |
| 8 | *n_+_*(-) | Positive (negative) ion number density | Number of positive (negative) ions per unit plasma volume |
| 9 | *n*_+(-)_*_0_* | Equilibrium positive (negative) ion number density | Equilibrium number of positive (negative) ions per unit quasi-neutral plasma volume |
| 10 | *n_0_* | Equilibrium (mean) solar plasma density | Equilibrium particle number per unit quasi-neutral solar plasma volume |
| 11 | *T_+_*(-) | Positive (negative) ion temperature | Ionic (positive/negative) thermal (kinetic) potential causing bulk plasma heat flow |
| 12 | *m_+_*(-) | Positive (negative) ion mass | Resistive measure of positive (negative) ions against a net force field causing its change of state (*m_+_*(-) ~ 1.67 × 10^-27^ kg) |
| 13 | *v_+_*(-) | Positive (negative) ion velocity | Directional speed of positive (negative) ion seen by a static observer with respect to heliocenter |
| 14 | *u_+_*(-) | Bohm velocity | Directional speed of positive (negative) ion to the plasma sheath for compensating their loss from the sheath towards a floating condition |
| 15 | *φ* | Electric potential | Amount of work done to move a unit charge from a reference point (*φ=*0) to a specific point against the electric field. |
| 16 | *ψ* | Gravitational potential | Amount of work done to move a unit mass from a reference point (*ψ=*0) to a specific point against the gravitational field. |
| 17 | *P_T+_*(-) | Positive (negative) ion partial pressure | Normal force per unit area applied by only a positive (negative) ionic fluid element to an object over which that force is distributed. |
| 18 | *P_T_* | Total pressure | Total normal force per unit area applied by a plasma fluid element to an object over which that force is distributed. |
| 19 | *j*_SIP (SWP)_ | SIP (SWP) electric current density | Amount of charge that flows through a unit area of SIP (SWP) elemental cross section per unit time. |
| 20 | *k_B_* | Boltzmann constant | It is a universal coupling constant between temperature and kinetic energy. (*k_B_*=1.38×10^-23^ J K^-1^) |
| 21 | *ε_0_* | Vacuum permittivity | A measure of the ability of electrical fields to pass through vacuum (*ε_0_=*8.85 × 10^−12^ C^2^ N^-1^ m^-2^) |
| 22 | *λ_De_* | Solar electron Debye length | A physical scale characterizing the minimum electrostatic polarization length (*λ_De_*=2×10^-12^ m) |
| 23 | *λ_J_* | Jeans scale length | Critical scale size representing a stable self-gravitating cloud (in hydrostatic equilibrium, *λ_J_* ~ 10^8^ m) |
| 24 | *G* | Universal gravitational constant | A coupling constant signifying Newton’s gravitational force field between two gravitating bodies (*G*=6.67×10^-11^ N m^2^ kg^-2^) |
