# Supplementary material for: Effects of negative ions on equilibrium solar plasmas in the fabric of gravito-electrostatic sheath model
Source: Sci Rep. 2024 Jul 12;14:16087. doi: 10.1038/s41598-024-66774-8 (PMC11245523; doi:10.1038/s41598-024-66774-8)
Supplement: Supplementary file 2 — Supplementary Information 2. [file 41598_2024_66774_MOESM2_ESM.doc]

**APPENDIX B: ASTRONOMICAL NORMALIZATION SCHEME**

| **S No** | **Normalized symbol** | **Normalizing parameter** | **Typical value^11^** |
| --- | --- | --- | --- |
| 1 | $\xi=r/\lambda_{J}$ | Jeans scale ($\lambda_{J}$) | 2×10^8^ m |
| 2 | $N_{e}=n_{e}/n_{e0}$ | Equilibrium quasi-neutral electron number density (*n_e0_=n_0_-n_-0_*) | Conditional (~10^30^ m^-3^ ) |
| 3 | $N_{+\left(-\right)}=n_{+\left(-\right)}/n_{+\left(-\right)0}$ | Equilibrium quasi-neutral positive (negative) ion number density ($n_{+\left(-\right)0}$, where, *n_+0_=n_0_=n_e0_+n_-0_*) | *n_0_*=10^30^ m^-3^ |
| 4 | $M_{+\left(-\right)}=v_{+\left(-\right)}/c_{s}$ | SIP sound speed (*c_s_=*$\sqrt{k_{B}T_{e}/m_{+}}$) | 3×10^5^ m s^-1^ |
| 5 | $\Phi=e\phi/k_{B}T_{e}$ | SIP electron thermal potential $\left(k_{B}T_{e}/e\right)$ | 10^3^ V |
| 6 | $\Psi=\psi/c_{s}^{2}$ | Square of the SIP sound speed (*c_s_*^2^, kinetic acoustic potential) | 9×10^10^ m^2^ s^-2^ |
| 7 | $P_{T+\left(-\right)}^{*}=P_{T+\left(-\right)}/P_{0}$ | Mean SIP pressure (*P_0_*) | 10^14^ N m^−2^ |
| 8 | $J_{SIP\left(SWP\right)}=j_{SIP\left(SWP\right)}/j_{B}$ | SIP Bohm electric current density (*j_B_*) | 4.8×10^16^ A m^−2^ |
