# Supplementary material for: Effects of negative ions on equilibrium solar plasmas in the fabric of gravito-electrostatic sheath model
Source: Sci Rep. 2024 Jul 12;14:16087. doi: 10.1038/s41598-024-66774-8 (PMC11245523; doi:10.1038/s41598-024-66774-8)
Supplement: Supplementary file 3 — Supplementary Information 3. [file 41598_2024_66774_MOESM3_ESM.doc]

**APPENDIX C: INITIAL AND INPUT VALUE**

| **S No** | **Physical parameter** | **SIP input** | **SWP input** |
| --- | --- | --- | --- |
| 1 | Radial distance | 10^-3^ | 3.75 |
| 2 | Gravitational field strength | 10^-5^ | NA |
| 3 | Electric potential | 10^-4^ | -1.172 |
| 4 | Electric field strength | 10^-4^ | -0.65 |
| 5 | Positive ion number density | 10^-4^ | Conditional |
| 6 | Positive ion Mach number | 10^-4^ | Conditional |
| 7 | Negative ion number density | 10^-4^ | Conditional |
| 8 | Negative ion Mach number | 10^-4^ | Conditional |
