# Supplementary material for: Effects of negative ions on equilibrium solar plasmas in the fabric of gravito-electrostatic sheath model
Source: Sci Rep. 2024 Jul 12;14:16087. doi: 10.1038/s41598-024-66774-8 (PMC11245523; doi:10.1038/s41598-024-66774-8)
Supplement: Supplementary file 4 — Supplementary Information 4. [file 41598_2024_66774_MOESM4_ESM.doc]

**APPENDIX D: BOHM CRITERION FOR GES-STRUCTURE WITH NEGATIVE IONS**

The presence of diverse negative ions in the solar and other stellar atmospheres has been well revealed by various observational methods in the past^14, 15^. The photoionization of the metal atoms in the cool stellar environments yields the electrons, which in turn, result in the formation of the negative ions, like H^-^, Cl^-^, C^-^, S^-^, OH^-^, C_2_^-^, CN^-^, SH^-^, H_2_O^-^, etc.^12^ The consideration of such negative ionic species is inevitable in the study of stellar plasma phenomena. Therefore, a tactical inclusion of the negative ion population in the GES-based solar plasma formalism is quite judicious and realistic. As a consequence, the corresponding Bohm sheath criterion for the modified GES-structure formation and subsequently modified solar plasma flow phenomena in the active presence of diverse negative ions is quite worth investigating.

In order to methodically derive the equivalent Bohm sheath criterion in the current formalism with negative ions, the standard approach of energy conservation principle along with the functional monotonicity rules is applied^40^. Accordingly, the positive ion speed at the sheath-exit location in the radially outward direction relative to the solar centre in the usual solar plasma symbolism^11^ can be written as

$u_{+}=\left[u_{0+}^{2}+\frac{2e}{m_{+}}\left(\phi_{0}-\phi\right)-2\left(\psi_{0}-\psi\right)\right]^{1/2}$. (A1)

The ion continuity equation in the sheath forming region can be expressed as

$n_{0+}u_{0+}=n_{+}u_{+}$. (A2)

The combination of equations (A1)-(A2) gives the positive ion number density as

$n_{+}=n_{0+}\left[1+\frac{2e}{m_{+}u_{0+}^{2}}\left(\phi_{0}-\phi\right)-\frac{2}{u_{0+}^{2}}\left(\psi_{0}-\psi\right)\right]^{-1/2}$. (A3)

Similarly, the negative ion number density as per the energy principle is obtained as

$n_{-}=n_{0-}\left[1+\frac{2e}{m_{-}u_{0-}^{2}}\left(\phi-\phi_{0}\right)-\frac{2}{u_{0-}^{2}}\left(\psi_{0}-\psi\right)\right]^{-1/2}$. (A4)

The equivalent electrostatic Poisson equation near the sheath-edge can be expressed after inclusion of the above derived densities of the plasma species as

$\partial_{r}^{2}\phi+\left(\frac{2}{r}\right)\partial_{r}\phi=\frac{e}{\varepsilon_{0}}\left[n_{e0} \text{exp} \left(\frac{e\phi}{k_{B}T_{e}}\right)+n_{0-}\left\{1+\frac{2e}{m_{-}u_{0-}^{2}}\left(\phi-\phi_{0}\right)-\frac{2}{u_{0-}^{2}}\left(\psi_{0}-\psi\right)\right\}^{-1/2}-n_{0+}\left\{1+\frac{2e}{m_{+}u_{0+}^{2}}\left(\phi_{0}-\phi\right)-\frac{2}{u_{0+}^{2}}\left(\psi_{0}-\psi\right)\right\}^{-1/2}\right]$. (A5)

After application of the same normalization scheme (Appendix B), the above equation can be expressed as

$\partial_{\xi}^{2}\Phi+\left(\frac{2}{\xi}\right)\partial_{\xi}\Phi=\left(\frac{\lambda_{J}}{\lambda_{De}}\right)^{2}\left[\left(\text{1-}\delta\right) \text{exp} \left(\Phi\right)+\delta N_{0-}\left\{1+2\left(\frac{m_{+}}{m_{-}}\right)\frac{1}{M_{0-}^{2}}\left(\Phi-\Phi_{0}\right)-\frac{2}{M_{0-}^{2}}\left(\Psi_{0}-\Psi\right)\right\}^{-1/2}-N_{0+}\left\{1+\frac{2}{M_{0+}^{2}}\left(\Phi_{0}-\Phi\right)-\frac{2}{M_{0+}^{2}}\left(\Psi_{0}-\Psi\right)\right\}^{-1/2}\right]$. (A6)

Equation (A6) is the nonlinear sheath evolution equation in the present GES-formalism. Here, equation (A6) is now multiplied by $\partial_{\xi}\Phi$ and integrated with respect to *ξ* from the illustrated sheath-entrance ($\xi_{0}=3.5$) to the sheath-exit ($\xi=4$) in the radially outward direction (Figs. 1-2) for analytical simplicity. The above operations can be mathematically written as

$\int_{\xi_{0}}^{\xi} \left\{\left(\partial_{\xi}^{2}\Phi+\frac{2}{\xi}\partial_{\xi}\Phi\right)\partial_{\xi}\Phi\right\}\partial\xi=\left(\frac{\lambda_{J}}{\lambda_{De}}\right)^{2}\left(1-\delta\right)\int_{\xi_{0}}^{\xi} \left\{\left(\text{exp}\Phi\right) \partial_{\xi}\Phi\right\} \partial\xi+$ $\left(\frac{\lambda_{J}}{\lambda_{De}}\right)^{2}\delta N_{0-}\int_{\xi_{0}}^{\xi} \left[\left\{1+2\left(\frac{m_{+}}{m_{-}}\right)\frac{1}{M_{0-}^{2}}\left(\Phi-\Phi_{0}\right)-\frac{2}{M_{0-}^{2}}\left(\Psi_{0}-\Psi\right)\right\}^{-1/2}\partial_{\xi}\Phi\right]\partial\xi-$ $\left(\frac{\lambda_{J}}{\lambda_{De}}\right)^{2}N_{0+}\int_{\xi_{0}}^{\xi} \left[\left\{1+\frac{2}{M_{0+}^{2}}\left(\Phi_{0}-\Phi\right)-\frac{2}{M_{0+}^{2}}\left(\Psi_{0}-\Psi\right)\right\}^{-1/2}\partial_{\xi}\Phi\right]\partial\xi$. (A7)

The LHS of the above equation for the development of a uniform monotonic potential structure (as confirmed by Fig. 4) yields the following parametric inequality

$\frac{1}{2}\left[\left(\partial_{\xi}\Phi\right)^{2}-\left(\partial_{\xi}\Phi\right)_{0}^{2}\right]+0.784 \text{ln}\left(\frac{\xi}{\xi_{0}}\right)>0$. (A8)

A methodical simplification of the RHS of equation (A7), considering the results obtained from equation (A8), yields the following analytic inequality

$\left(\text{1-}\delta\right) \text{exp} \left(\Phi\right)>N_{0+}\left\{1+\frac{2}{M_{0+}^{2}}\left(\Phi_{0}-\Phi\right)-\frac{2}{M_{0+}^{2}}\left(\Psi_{0}-\Psi\right)\right\}^{-1/2}$. (A9)

Now, from equation (A9), one gets the Bohm sheath criterion in the present context given as

$M_{0+}>\sqrt{\frac{0.626-2\left(\Psi_{0}-\Psi\right)}{\left(1-\delta\right)^{-2}N_{0+}^{2}\text{exp} \text{(2.344)} -1}}$, for $0\leq\delta<1/15$. (A10)

The above inequality is recognized as the equivalent Bohm sheath criterion for the modified GES formation in the presented GES-formalism. In the above derivation, the negative ion concentration in the sheath region is neglected, as supported by Fig. 12. It may be restated here that the symbols with “0” subscript denote the corresponding quantity at the sheath-entrance location in the SIP. The symbols without any subscript indicate the respective quantities in the sheath-exit location along the radially outward direction. It is seen that the Bohm threshold velocity is *δ*-sensitive, and is applicable for the practical range of $0\leq\delta<1/15$.


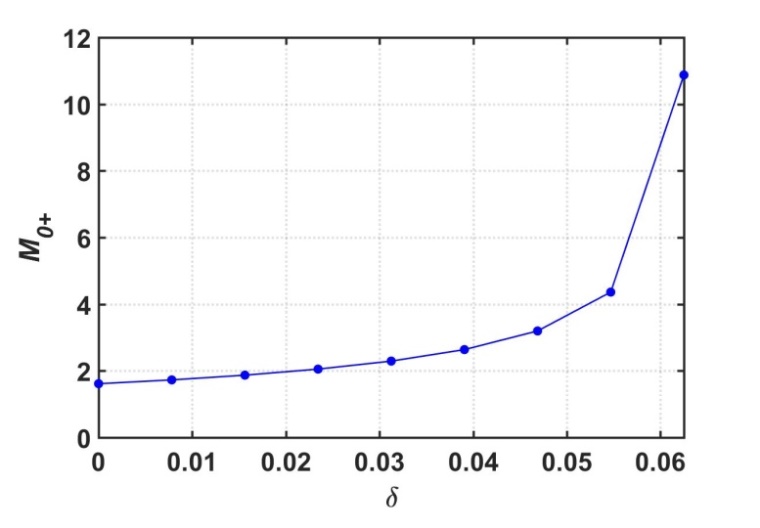


**Fig. A1:** Profile of the *δ-*modified Bohm sheath criterion for positive ion (*M_0+_*) in the GES fabric with practically allowed *δ*-variation (with fixed *m_i_/m-=*1, *T_i_/T_e_*=1 and *T-/T_e_=*1).

As shown in Fig. A1, the profile of the Bohm Mach number with varying negative ion concentration is portrayed according to equation (A10). It is seen that the realistic Bohm Mach values are supersonic and it increases in magnitude with an increase in the *δ*-value and vice-versa. In other words, an increase in the negative ion concentration causes higher positive ion drifting speed to account for the loss of ions in the sheath. Interestingly, it is found further that the *δ*-modified Bohm threshold ion-speed value for *δ*=0 (*M_0+_* > 1.61) obtained here is in good agreement with the basic GES-picture without negative ions (*M_0_* >
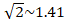
) previously reported elsewhere^6^. Consequently, it pronounces a fair reliability of the presented GES-based analysis with negative ions against the previously reported basic GES formalism in a simplified form without negative ions for the first time.

A comparative analysis of the equivalent Bohm sheath criterion in terms of the Bohm threshold velocity values derived previously without^6^ and presently with negative ions in the current study can be given as: $\Delta M_{0+}/M_{0}\times100\%=$ $\left[\left(M_{0+}-M_{0}\right)/M_{0}\right]\times100\%$ $=14 .18\%$, where *M_0+_* ≈ 1.61 and *M_0_*
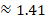
, as already shown above. Thus, it is found herein that there is a noticeable increment in the Bohm threshold velocity value of the plasma flow in the sheath-entrance point in the presence of the negative ions. Therefore, it can be inferred, herein, that the presence of negative ions (even if in minority) can enhance the loss process of the positive ions in the sheath region. So, the positive ions drift with higher speed into the sheath region to compensate the positive ion loss for endurance of the bounded GES structure in a steady-state form. This process is highly prominent towards the higher *δ*-values (Fig. A1). It shows how the negative ion inclusion in our semi-analytic study of the modified GES formation condition (Bohm threshold criterion) is well justified and reliably validated.

It is to be mentioned here in the present context that the Bohm threshold criterion in the laboratory scaled electronegative plasmas can be expressed as

$M_{0+}\geq\left[\frac{k_{B}T_{e}\left(1+\alpha_{0}\right)}{m_{+}\left(1+\alpha_{0}\gamma\right)}\right]^{1/2}$, (A11)

considering the local plasma quasi-neutrality in the sheath edge regions^41^. Here, $\alpha_{0}\equiv n_{0-}/n_{0e}$ and $\gamma\equiv T_{e}/T_{i}$. So, it can clearly be interpreted that, if $\alpha_{0}$ is not too small, and $\gamma$ is large (i.e., for cold ions), the negative ions highly reduce the Bohm (threshold) Mach value^41^. On the contrary, in our model, an opposite behavior in terms of the Bohm threshold value is noticed (Fig. A1). Interestingly, the physical reason behind it is attributable to the significant deviation from the local quasi-neutrality condition of the SIP, as already discussed above in the main text.

It is to be noted here that the Mach number in a plasma fluid element (normalized value of *v*_+_) near the sheath region is subsonic in nature (Fig. 5). This subsonic ionic flow value is accountable for the origin of the supersonic solar wind outside the SSB, via the gravito-electrostatic force field action. However, the Bohm Mach number value (normalized value of *u*_0+_) is supersonic in nature independent of *δ* (Fig. A1). This supersonic ionic flow is responsible for the formation and existence of the solar plasma sheath as a modified bounded GES structure. These two distinct positive ionic processes of subsonic and supersonic flow behaviours are clearly depicted in Fig. 1.
