# Supplementary material for: Effects of negative ions on equilibrium solar plasmas in the fabric of gravito-electrostatic sheath model
Source: Sci Rep. 2024 Jul 12;14:16087. doi: 10.1038/s41598-024-66774-8 (PMC11245523; doi:10.1038/s41598-024-66774-8)
Supplement: Supplementary file 5 — Supplementary Information 5. [file 41598_2024_66774_MOESM5_ESM.doc]

**APPENDIX E: OUR MODEL VERSUS EXISTENT MODELS**

| **S No** | **Item** | **Prediction (by us)** | **Prediction (by others)** |
| --- | --- | --- | --- |
| 1 | Negative ion density (*δ*) | Broader *δ*-values (0, 0.001, 0.1, 0.25, 0.5) with the observed one (*δ*=0.001) included. | Experimentally confirmed for sun-like stars, *δ* ≈ 0.001^12^, where every metal atom contributes one electron to form negative ions. |
| 2 | Mach number at SSB | For *δ*=0.001, *M*_SSB_=1.3×10^-8^; supersonic transition occurs at *r* ≈ 84 *λ_J_* (1.68×10^10^ m). So, our model creates observed corona-like regions for supersonic solar wind origin as per SSM. | Solar wind particles originate from the corona, the outermost part of the solar atmosphere. This region is extended to 10-20 times solar radius from the solar surface, i.e., *r* ~ (40-75) *λ_J_* ^23, 28^. |
| 3 | Mach number at 1 au | *M*_1 au_ ~ 1.13 for *δ*=0.001 and *m_i_/m-*=1, *T_i_/T_e_*=*T-/T_e_*=1.25, i.e. solar wind particles travel with speed ~ 3.39×10^5^ m s^-1^, fairly matching observations. | 1. Slow solar wind speed 2.5×10^5^–4×10^5^ m s^−1^ at 1 au^16, 18^; 2. Fast solar wind speed 4×10^5^–8×10^5^ m s^−1^ at 1 au^16, 18^. |
| 4 | Solar boundary | For *δ*=0.001, the SSB forms at 3.75 *λ_J_* (=7.5×10^8^ m). | Radius of the Sun (photospheric radius) is determined as 6.96×10^8^ m^42^. |
| 5 | Plasma constitutive temperature ratio | In our model, *T_i_/T_e_*= 0.75, 1, 1.25, 1.5, 1.75. | Observed values of solar wind constituent temperature ratios from various missions at 1 au:   1. Cluster^17, 43^: *T_i_/T_e_* < 0.1 to *T_i_/T_e_* > 10; 2. Explorer 34^17, 44^: *T_i_/T_e_* ∼ 0.2-0.67; 3. Imp 6, 7, and 8^17, 45^: *T_i_/T_e_* < 0.2 to *T_i_/T_e_* > 2; 4. ISEE 3^17, 46^: *T_i_/T_e_* ∼ 0.12-10; 5. Vela 3 & 4^17, 47^: *T_i_/T_e_* ∼ less than 0.1 to 2.5 for slow solar wind and 0.17-5.0 for fast solar wind; 6. Wind^17, 48^: *T_i_/T_e_* ∼ 0.62-3.33. |
| 6 | Internal structure | Highly populated dense regions lie in *ξ*=0-0.5, i.e. heliocenter to 10^8^ m in SIP, matching with the SSM value. | In SSM, the highly dense core lies from centre to 2.09×10^8^ m^42^. |
| 7 | Negative ion population | SIP is not favourable to heavy negative ion formation; lower the negative ion mass, higher is its population density and vice-versa. | Observationally, the H^-^ ion (lightest) accounts for the major part of the continuous absorption of the solar atmosphere due to high population density; other negative ions (rare) have been identified later with advanced spectrophotometry^14, 15^. |
