# Supplementary material for: Effects of negative ions on equilibrium solar plasmas in the fabric of gravito-electrostatic sheath model
Source: Sci Rep. 2024 Jul 12;14:16087. doi: 10.1038/s41598-024-66774-8 (PMC11245523; doi:10.1038/s41598-024-66774-8)
Supplement: Supplementary file 6 — Supplementary Information 6. [file 41598_2024_66774_MOESM6_ESM.doc]

**APPENDIX F: RELIABIITY COMPARISON AMONG DIVERSE GES FORMALISMS**

| **S No** | **Item** | **GES-1** | **GES-2** | **Deviation (GES:1-2)** | **GES-3** | **Deviation (GES:1-3)** | **Physics (mechanism)** |
| --- | --- | --- | --- | --- | --- | --- | --- |
| 1 | SSB | 3.50 (Boltzmann electrons) | 3.50 (specially for Boltzmann electrons) | 0.00 | 3.75 (Boltzmann electrons) | -0.25 | Shielding by negative ionic species |
| 2 | Electric potential at SSB | -1.00 | -1.02 | 0.02 | -1.17 | 0.17 | High density action |
| 3 | Potential at 1 au | -30.00 | -4.68×10^4^ | 4.68×10^4^ | -4.80×10^4^ | 4.80×10^4^ | Low density action |
| 4 | Mach number at SSB | 1.00×10^-7^ | 1.66 (for *κ*=2) | -1.66 | 1.30×10^-8^ | 8.70×10^-8^ | High collisions |
| 5 | Mach number at 1 au | 3.50 | 0.54 (for *κ*=2) | 2.96 | 1.13 | 2.37 | Less collisions due to diffusivity |
| 6 | Current density at SSB | Silent | −0.33 (for *κ*=2) | NA | 0.83 (for *δ*=0.001) | NA | Same as *M*_SSB_ |
| 7 | Current density at 1 au | Silent | −1.91×10 ^−8^ (for *T_e_/T_i_* =7.5) | NA | 0.30 (for *δ*=0.001) | NA | Same as *M*_1 au_ |
| 8 | Electron occupancy | Silent | Near the heliocenter | NA | Near the heliocenter | NA | Low net electrostatic potential due to high effective shielding |
| 9 | Positive ion occupancy | Silent | Silent | NA | Near the heliocenter for *δ*=0.001 | NA | Same as electron occupancy |
| 10 | Negative ion occupancy | Silent | Silent | NA | Near the heliocenter | NA | Same as electron occupancy |
| 11 | *E*-gradient structure | Silent | Significant within *r*= 0 - 0.2 *λ_J_* | NA | Significant within *r*=0-0.5 *λ_J_* | NA | High density of the heliocore |
| 12 | *g*-gradient structure | Silent | Significant within *r*=0-0.2 *λ_J_* | NA | Uniform and high within *r*=0-0.5 *λ_J_* | NA | High density of the heliocore |
| 13 | Polytropicity | Silent | *κ*-modified | NA | Silent | NA | Hydrostatic equilibrium |
| 14 | Magneto-activity | Silent | Included | NA | Silent | NA | Local dynamo action |
| 15 | Gradient scales | Silent | Depicted | NA | Well depicted | NA | Global gravito-electrostatic interplay |
| 16 | Turbulence | Silent | Included | NA | Silent | NA | Equilibrium local gradient forces |
| 17 | Deviation from plasma quasi-neutrality | Silent | Silent | NA | Well explored | NA | Self-structurization |
| 18 | Reliability | Moderate | Extensive | NA | Extensive | NA | Realistic negative ions considered |
